# Local Temperature Increments and Induced Cell Death in Intracellular Magnetic Hyperthermia

**DOI:** 10.1021/acsnano.3c00388

**Published:** 2023-03-20

**Authors:** Yuanyu Gu, Rafael Piñol, Raquel Moreno-Loshuertos, Carlos D. S. Brites, Justyna Zeler, Abelardo Martínez, Guillaume Maurin-Pasturel, Patricio Fernández-Silva, Joaquín Marco-Brualla, Pedro Téllez, Rafael Cases, Rafael Navarro Belsué, Debora Bonvin, Luís D. Carlos, Angel Millán

**Affiliations:** † INMA, Institute of Nanoscience and Materials of Aragon, CSIC-University of Zaragoza, C/Pedro Cerbuna 12, 50009 Zaragoza, Spain; ‡ School of Materials Science and Engineering, Nanjing Tech University, 210009, Nanjing People’s Republic of China; § Department of Biochemistry and Molecular and Cellular Biology, and Institute for Biocomputation and Physics of Complex Systems, University of Zaragoza, C/Pedro Cerbuna 12, 50009 Zaragoza, Spain; ∥ Phantom-g, CICECO-Aveiro Institute of Materials, Department of Physics, University of Aveiro, Campus de Santiago, 3810-193 Aveiro, Portugal; ⊥ Faculty of Chemistry, University of Wroclaw, 14. F. Joliot-Curie Street, 50-383 Wroclaw, Poland; # Department of Power Electronics, I3A, University of Zaragoza, 50018 Zaragoza, Spain; ▽ Powder Technology Laboratory, Institute of Materials, Ecole Polytechnique Fédérale de Lausanne, 1015 Lausanne, Switzerland

**Keywords:** trivalent lanthanide ions, magnetic hyperthermia, luminescence thermometry, local hyperthermia, intracellular thermometry

## Abstract

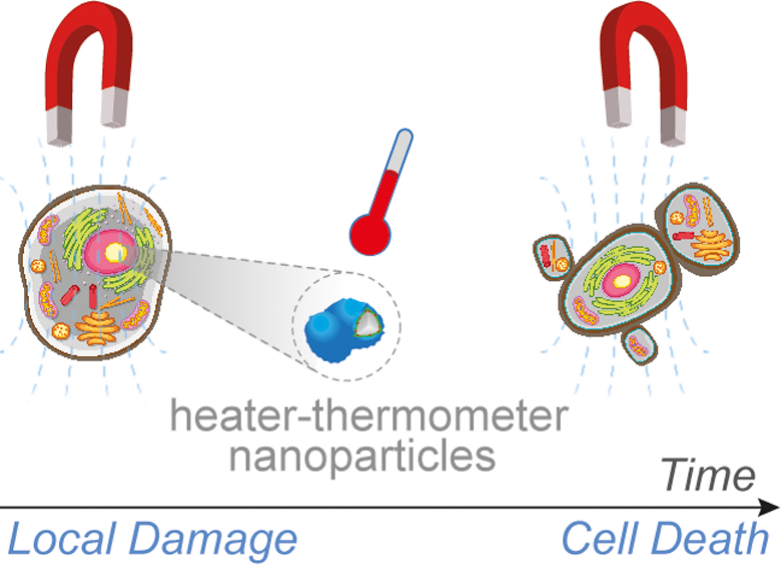

The generation of temperature gradients on nanoparticles heated externally by a magnetic field is crucially important in magnetic hyperthermia therapy. But the intrinsic low heating power of magnetic nanoparticles, at the conditions allowed for human use, is a limitation that restricts the general implementation of the technique. A promising alternative is local intracellular hyperthermia, whereby cell death (by apoptosis, necroptosis, or other mechanisms) is attained by small amounts of heat generated at thermosensitive intracellular sites. However, the few experiments conducted on the temperature determination of magnetic nanoparticles have found temperature increments that are much higher than the theoretical predictions, thus supporting the local hyperthermia hypothesis. Reliable intracellular temperature measurements are needed to get an accurate picture and resolve the discrepancy. In this paper, we report the real-time variation of the local temperature on γ-Fe_2_O_3_ magnetic nanoheaters using a Sm^3+^/Eu^3+^ ratiometric luminescent thermometer located on its surface during exposure to an external alternating magnetic field. We measure maximum temperature increments of 8 °C on the surface of the nanoheaters without any appreciable temperature increase on the cell membrane. Even with magnetic fields whose frequency and intensity are still well within health safety limits, these local temperature increments are sufficient to produce a small but noticeable cell death, which is enhanced considerably as the magnetic field intensity is increased to the maximum level tolerated for human use, consequently demonstrating the feasibility of local hyperthermia.

One of the biggest challenges in cancer therapy is to reduce the unpleasant secondary effects of surgical and chemotherapy treatments while enhancing the survival rate. The emergence of magnetic hyperthermia a few decades ago seemed to represent a great opportunity to achieve this goal.^1−4^ In the ideal case, nontoxic iron oxide magnetic nanoparticles (MNPs) could be selectively internalized in tumor tissue and then be heated by a noninvasive external alternating magnetic field (AMF), thereby causing tumor cell destruction without affecting the rest of the body.^4,5^ However, it soon became clear that the intrinsic low heating power of MNPs allowed for human use at conditions for magnetic field induction represented a severe limitation and restricted the general implementation of this technique in hospitals.^6^ Consequently, to make this approach viable, a massive amount of MNPs had to be injected, and thermocouples (invasive) or luminescent thermometers (remote detection) had to be placed around a tumor to avoid overheating in the surrounding healthy tissue.^7−12^ Yet there is a promising alternative to this unfeasible procedure, which turns magnetic hyperthermia back into an ideal, nontoxic, and noninvasive proposition: cancer cell death can be attained by small amounts of heat generated at thermosensitive intracellular sites, without significantly increasing the average temperature of the whole tumor (or even that of the cell). This local intracellular hyperthermia hypothesis^2,4,13^ can only hold true if the heat power generated in the MNPs reaches temperature increments that are high enough to induce irreversible local damage to the surrounding cell biomolecules and consequently trigger cell death.

According to theoretical thermodynamic calculations of heat transfer,^2,14−16^ it was predicted that the heat generated in a single MNP would be dissipated to the rest of the cell before reaching any significant temperature difference with its surroundings. However, contrary to the theoretical predictions, experiments involving magnetic hyperthermia in MNPs in water suspensions^17−20^ and powder suspensions,^21^ both *ex vivo*(22) and *in vivo*,^23^ found relevant local temperature increases in MNPs with respect to the bulk water temperature. The occurrence of these temperature gradients in cells would support the local hyperthermia hypothesis. But results in water suspensions cannot simply be extrapolated to the cell environment, given that they are subject to considerable differences in terms of heat transfer efficiency and MNP mobility, which can cause major changes in magnetic heating processes.^15^ Therefore, reliable intracellular temperature measurements are needed to get an accurate picture and clear up the discrepancy.

Up to now, distinct luminescent intracellular thermometers have been reported, including organic dyes,^24,25^ oligonucleotides,^19^ fluorescent proteins,^26−28^ polymers,^29,30^ nanodiamonds,^31,32^ quantum dots,^33^ and lanthanide-doped nanoparticles.^34−37^ Intracellular thermometry measurements in living cells have shown spontaneous thermogenesis,^38−40^ inhomogeneous temperature progressions,^24,27^ and “hot organelles”, including the mitochondria,^41,42^ the nucleus,^29^ and nucleoli.^35^ These reports, however, have been hotly debated, given the questions surrounding the observation of intracellular temperature gradients based on simple thermodynamic arguments that predicted intracellular temperature fluctuations of less than several millikelvins.^30,43,44^ Moreover, cell temperature gradients go beyond intracellular temperature measurements and extend to endogenous heat generation in general.^32,40,45−47^ A detailed discussion of this controversy, which extends to many other nanoscale heat transfer phenomena,^48^ is provided in Section I in the Supporting Information.

In recent years, significant progress in the techniques used to measure the local temperature of nanoheaters in water suspensions and cells has been achieved thanks to the concomitant advancements in luminescence nanothermometry (see Section I in the Supporting Information for a comprehensive overview). Of the distinct examples reported so far, three studies have reported on measuring cell temperature during exposure to an external AMF.^24,49,50^ In the first one,^24^ the temperature determination was performed on MNPs that were fixed to the cell membrane and were therefore not internalized within the cells. In the second one,^49^ a single-particle approach (a cyanine dye thermometer probe separated a distance of 7 nm from the MNP surface) was employed to follow the temperature of MNPs encapsulated in cellular lysosomes through a single emission of the dye. In the third one,^50^ a dual-particle approach (green fluorescence protein, GFP, bound to actin filaments and separated from MNPs) was used to measure the intracellular temperature through the GFP lifetime. We notice that in these last two works^49,50^ single-^34^ and dual-particle^31,51^ approaches were used separately, therefore not allowing an evaluation of the influence of the uncontrolled spatial distribution of nanoheaters and nanothermometers in the local temperature measurements and not permitting a comparison of the dynamics of the intracellular temperature increments when the heaters and the thermometers were chemically bonded or physically separated.

In this paper, we demonstrate the viability of the local hyperthermia hypothesis by reporting real-time intracellular temperature increments during exposure to an external AMF using single- and dual-particle heater/thermometer nanostructures. The single-particle approach is composed of heater–thermometer core@shell NPs formed by a γ-Fe_2_O_3_ core coated by a polymeric shell embedded with Sm^3+^- and Eu^3+^-bearing complexes for ratiometric luminescence temperature sensing, thereby allowing access to intracellular temperature increments on the surface of the MNPs. In addition, Sm^3+^/Eu^3+^-bearing thermometric nanomicelles not chemically linked to γ-Fe_2_O_3_ MNPs (dual-particle approach) are used to measure the temperature increments in both intracellular and extracellular media induced by the AMF application. While the extracellular temperature remains virtually unaffected as the MNPs are heated, the observed maximum intracellular temperature increment in the vicinity of MNPs is lower (5.9 °C) than what is measured on the surface of MNPs using the heater–thermometer core@shell NPs (8.0 °C), and it takes double the time to reach it. These differences are explained by the fact that thermal sensing is not achieved at the heating volume in the dual-particle approach. Therefore, a distribution of interparticle distances exists, and the measured temperature corresponds to an average value instead of a precise one ascribed to a particular heater–thermometer distance.^34^ For a large distribution of interparticle distances (and in the majority of the cases, we do not know if the distribution is large or small), the meaning of the average temperature is vague, which can be viewed as a limitation of the dual-particle approach for precise intracellular temperature readouts. The most relevant outcome of this study is that even for moderate magnetic fields (with an amplitude of *H* = 30 mT at a frequency of *f* = 100 kHz and *Hf* = 2.4 × 10^9^ A·m^–1^·s^–1^) which are still well within the healthy limit value^52^ (*Hf* = 5 × 10^9^ A·m^–1^·s^–1^), local temperature increments induce noticeable cell death, which is considerably enhanced as the magnetic field intensity is increased to the maximum tolerated level for human use. These findings are a breakthrough in the path toward implementing the local magnetic hyperthermia concept in the clinic.

## Results and Discussion

### Ratiometric Molecular Temperature Probes

**Figure 1:**
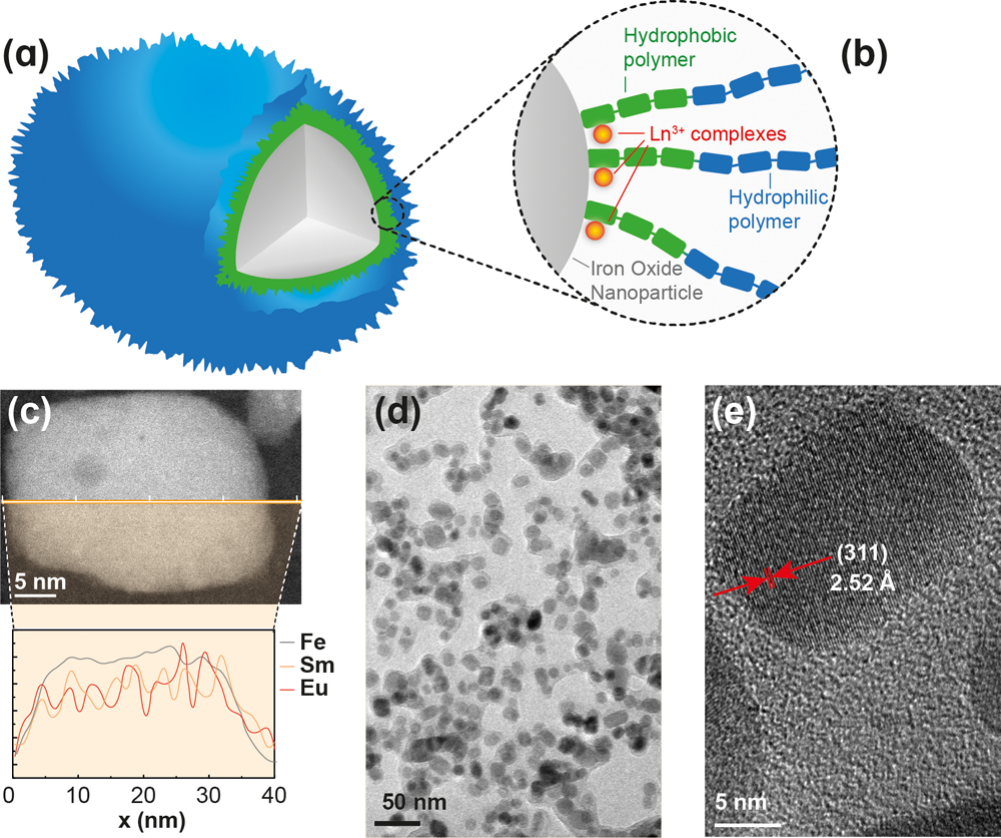
(a) Scheme of the dual heater–thermometer core@shell NPs composed of a γ-Fe_2_O_3_ magnetic core (which dissipates heat under irradiation with an external AMF) and an amphiphilic copolymer shell and (b) details of the chemical structure of the hydrophobic block (in green), including covalently bonded terpyridine residues that fixed the molecular Sm^3+^ and Eu^3+^ thermometric chelates, and a hydrophilic biocompatible PEG methacrylate block (in blue). (c) EDS composition profile of the Fe, Sm, and Eu elements across a nanoparticle. (d) TEM images of the dual heater–thermometer core@shell NPs. (e) Magnification of a single NP showing the interplanar distance between (331) planes.

Dual heater–thermometer core@shell nanostructures were prepared by coating γ-Fe_2_O_3_ NPs with an amphiphilic copolymer P(MPEGMA-st-PEGMA)-*b*-P(4VP-st-VBPTpy), which incorporated the Ln(BNPD)_3_·2H_2_O (Ln = Sm or Eu) chelates by coordination with (VBPTpy) ligands in the polymer (details in Section II in the Supporting Information). A scheme of the dual heater–thermometer core@shell NPs and the amphiphilic copolymer used for the coating is shown in Figure 1a,b. The energy dispersive X-ray spectroscopy (EDS) composition profile across the dual heater–thermometer core@shell NPs is in accordance with the location of the thermometric Sm^3+^ and Eu^3+^ ions on the surface of the iron oxide NPs (Figure 1c), which had a size of 20 ± 5 nm, as determined by transmission electron microscopy (TEM) images (Figure 1d,e and Figure S7c in the Supporting Information). The hydrodynamic diameter of the dual heater–thermometer core@shell NPs was 50 ± 13 nm, as determined by dynamic light scattering (DLS) (Figure S10b and Table S5 in the Supporting Information). Single Sm^3+^/Eu^3+^-bearing thermometric nanomicelles were formed by the self-assembly of an amphiphilic copolymer with a hydrophobic block of P(CholA-st-PhenA) containing the auxiliary ligand 4-(acryloyloxymethyl)-1,10-phenanthroline (PhenA) and a hydrophilic block of P(MPEGA-st-PEGA), similar to that of the dual heater–thermometer core@shell NPs (Figures S2, S3 in the Supporting Information). The PhenA ligand fixes the Sm^3+^ and Eu^3+^ aqua-complexes inside the hydrophobic part of the micelles by the formation of Ln(BNPD)_3_(PhenA) (Ln = Sm, Eu) tetracoordinate chelates, which show enhanced intensity and chemical stability with respect to the aqua-complexes. The Sm^3+^ and Eu^3+^ ion content in the micelles was 1.53% w/w, and their size was 19 ± 5 nm (Figure S10c and Table S5 in the Supporting Information).

**Figure 2:**
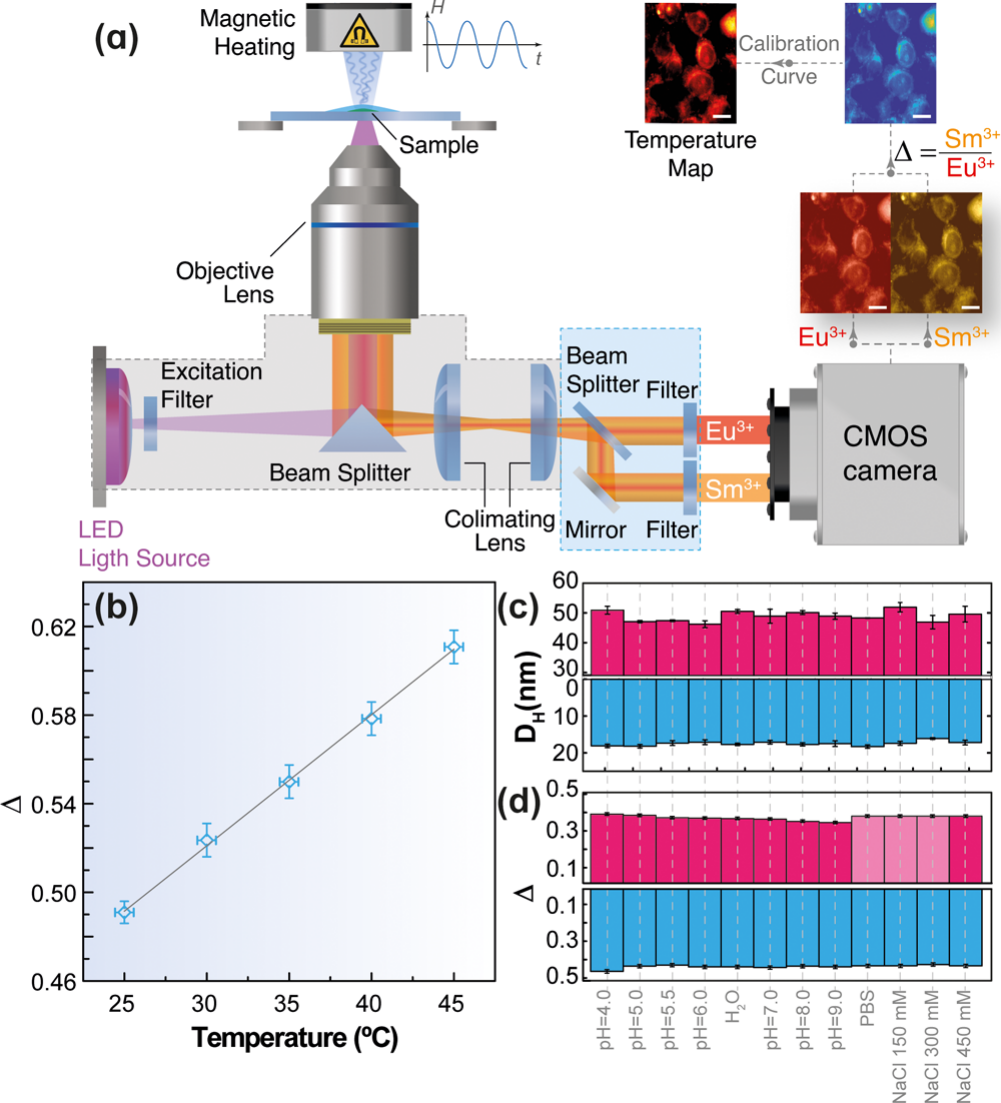
(a) Scheme of the microscope temperature imaging setup. The scale bars correspond to 10 μm. (b) Intensity ratio-to-temperature calibration for dual heather–thermometer core@shell NPs in the collagen gel. The temperature was measured by a semiconductor sensor (Neoptix Reflex). The straight line is the best fit to the data corresponding to Δ = (5.9 ± 0.1) × 10^–3^*T* + (34.5 ± 0.3) × 10^–2^ (°C) (*r*^2^ > 0.998). (c) Hydrodynamic diameter (*D*_H_) and (d) thermometric parameter (Δ) of the dual heather–thermometer core@shell NPs (magenta) and the Sm^3+^/Eu^3+^-bearing thermometric nanomicelles (blue) for distinct pH values, ionic strength, and incubation media. The data points highlighted in light magenta were extrapolated from the NaCl 450 mM measurement according to the independence of the Δ parameter from the ionic strength observed in Sm^3+^/Eu^3+^-bearing thermometric nanomicelles.

The thermometric parameter, Δ = *I*_1_/*I*_2_, is defined by the ratio between the emission intensities of the Sm^3+^^4^G_5/2_ → ^6^H_9/2_ (*I*_1_) and Eu^3+^^5^D_0_ → ^7^F_2_ (*I*_2_) transitions. The ratiometric nature of the thermometer ensures the reading of absolute temperatures.^53−57^ Moreover, the *I*_1_ and *I*_2_ thermal dependencies are explained by the balance between ligand-to-ion (Sm^3+^ or Eu^3+^) forward and backward intramolecular energy transfer processes,^58^ as demonstrated in similar Sm^3+^/Eu^3+^ thermometric nanomicelles.^35^ The temperature imaging is done in an in-house-developed microscopy system consisting of a conventional fluorescence microscope equipped with a beam splitter that splits the emission beam in two. Each of the beams is filtered to select the spectral regions corresponding to *I*_1_ (Sm^3+^ emission) and *I*_2_ (Eu^3+^ emission).^35^ Details of the setup are given in Figure 2a and Figures S14, S15 in the Supporting Information.

To mimic the conditions of the temperature measurements in cells, the thermometric performance of the NPs was evaluated by dispersing them in a collagen gel (Materials and Methods) and then measuring the emission intensity using the microscope temperature imaging setup in both the Sm^3+^ and Eu^3+^ channels (Figure 2a) for externally applied temperatures between 25 and 45 °C. For each temperature, the mean intensity values (and the corresponding standard deviations, SD) obtained in a representative region of 20 × 20 μm^2^ were used to calculate Δ (and the corresponding uncertainty, δΔ). The resulting calibration points (Figure 2b) were fitted to a straight line that was used as the calibration curve to convert the ratiometric relationship between the intensities in the Sm^3+^ and Eu^3+^ channels into temperature through a dedicated MatLab routine. The corresponding relative thermal sensitivity, *S*_r_, ranges between 1.20%·°C^–1^ (at 25 °C) and 0.98%·°C^–1^ (at 45 °C) (Figure S18 in the Supporting Information). These values are comparable with those previously reported by us for lanthanide-bearing thermometric nanomicelles.^34,35^ Similar thermometric performance was obtained for NPs dispersed in water suspensions (Figures S17, S18 in the Supporting Information).

In living cells, the microenvironmental conditions, such as pH and ionic strength, change with time and from one organelle to another.^36,37,44^ Thus, a quantitative analysis of the intracellular temperature dynamics using luminescent nanothermometers requires colloidal stability and optical performance that is independent of the microenvironmental conditions. The mean hydrodynamic size (Figure 2c) and the thermometric parameters (Figure 2d) of dual heater–thermometer core@shell NPs and Sm^3+^/Eu^3+^-bearing thermometric nanomicelles are not affected by the ionic strength, pH level, and incubation media, which indicates colloidal stability and constant optical performance of the NPs.

### Cell Cultures, Cell Internalization Quantification, Localization of NPs, and Toxicology

Three sets of magnetic hyperthermia experiments were performed on incubated MDA-MB-468 breast cancer cells to determine the temperature during AMF application using Sm^3+^/Eu^3+^-based luminescent thermometers located at distinct distances from the magnetic heating source. In the first set (labeled as I), the cells were incubated with dual heater–thermometer core@shell NPs (thermometers located on the surface of the heaters), while in the second experiment (labeled as II), rhodamine B (RhB)-labeled MNPs and single Sm^3+^/Eu^3+^-bearing thermometric nanomicelles were used. In both experiments, the incubation with the NPs and nanomicelles was long enough to allow their internalization in the cells. In the third set of experiments (labeled as III), the cells were incubated with RhB-labeled MNPs for 24 h, and the single Sm^3+^/Eu^3+^-bearing thermometric nanomicelles were added to the culture just before the application of the magnetic field. This means that in experiment III the Sm^3+^/Eu^3+^-bearing nanomicelles remained outside the cell membrane.

**Figure 3:**
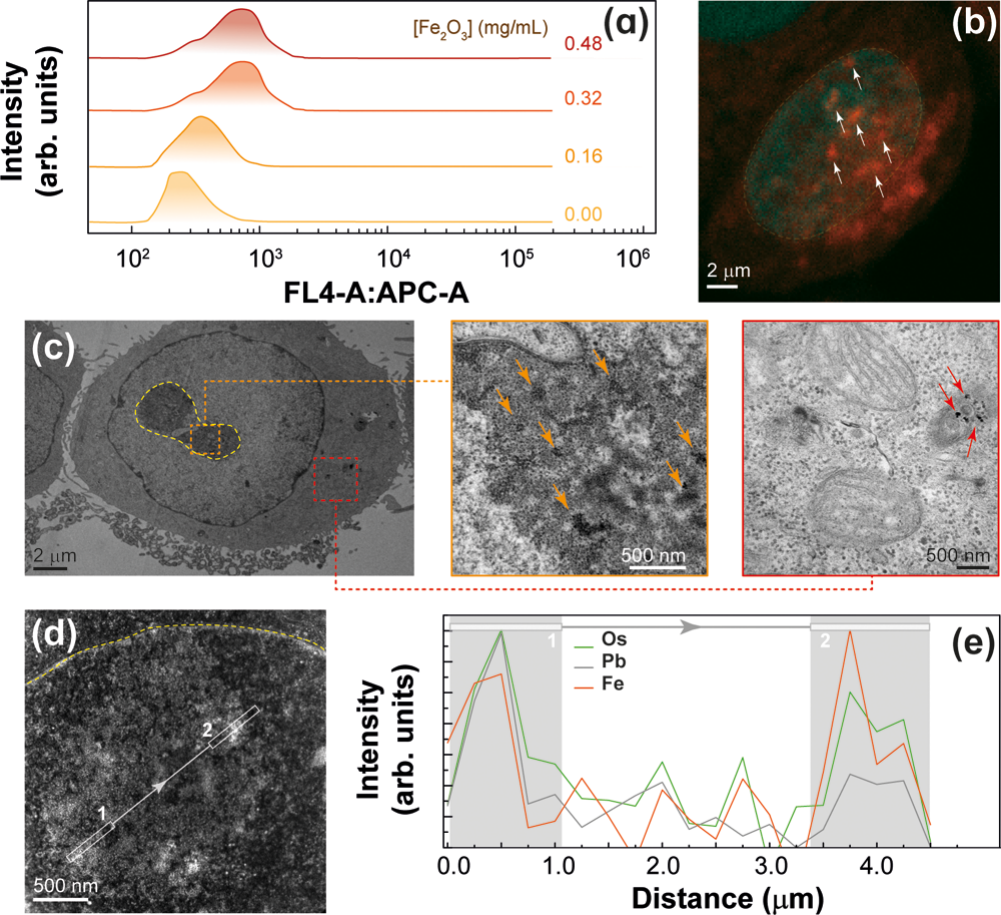
(a) MDA-MB-468 cell internalization of dual heater–thermometer core@shell NPs after incubation at different concentrations determined by cell cytometry. (b) Confocal image of the MDA-MB-468 cells after incubation with RhB-labeled MNPs. The arrows mark some red spots assigned to the emission of the dye inside the nucleolus stained with blue color Hoechst. (c) TEM and (d) STEM images of ultramicrotomes of the MDA-MB-468 cells after incubation with MNPs. Osmium tetroxide and uranyl and lead acetate were used for staining. The images show a whole cell in (c) and the interior of the nucleolus in (d). The insets in (c) present details of the nucleolus (orange) and of the cytoplasm (red), with the mitochondria and dark objects of a similar size to that of the MNPs in its vicinity. (e) EDS profile from the STEM image in (d) confirms the location of the MNPs in the nucleolus. Pb and Os signals increase notably as the Fe signal increases, indicating a preferential absorption of the staining molecules on the copolymer coating.

All the cells were observed by optical microscopy before and after incubation with the distinct NPs. The morphology of the cells did not change after incubation, as observed in phase-contrast images taken by color and CMOS cameras (Figures S27, S28 in the Supporting Information). Fluorescence images of control cells did not show any emission, in either the Sm^3+^ or the Eu^3+^ channels (Figure S29a in the Supporting Information), but the fluorescence images of cells incubated with the dual heater–thermometer core@shell NPs or the single Sm^3+^/Eu^3+^-bearing thermometric nanomicelles showed clear luminescence in both channels (Figure S29b in the Supporting Information), thereby indicating that the collected emission comes exclusively from the Sm^3+^ and Eu^3+^ ions in the NPs. Internalization of the NPs was studied by chemical analysis and cell cytometry. ICP-OES analysis of the supernatant after incubation with the dual heater–thermometer core@shell NPs yielded 2.1 × 10^–12^ g of Fe_2_O_3_ per cell. According to the cytometry measurements, the internalization ratio doubled when the concentration of NPs in the culture was increased from 0.16 to 0.32 mg(Fe_2_O_3_)·mL^–1^, but there was no further increase for higher values up to 0.48 mg(Fe_2_O_3_)·mL^–1^ (Figure 3a). Annexin V assays did not show any significant cytotoxic effect of the NPs (Figure S21 in the Supporting Information).

Confocal fluorescence images of the MDA-MB-468 cells after incubation with RhB-labeled MNPs showed a uniform distribution in the cytoplasm, although occasionally they had also accumulated in the nucleus and inside the nucleolus (Figure 3b and Figures S30–S32 in the Supporting Information), which is quite rare for NPs. Ultramicrotomes of cell cultures were observed by TEM after staining with osmium tetroxide, uranyl acetate, and lead acetate (Figure 3c,d), and EDS analysis confirmed the presence of NPs inside the nucleolus (Figure 3e and Figure S23 in the Supporting Information). Colocalization experiments of RhB-labeled MNPs with lysosomes showed no evidence of a relevant accumulation of NPs in those organelles (Figure S33 in the Supporting Information), as it has often been reported.^59−61^ To the contrary, staining with MitoTracker indicated a strong colocalization of MNPs with the mitochondria (Figure S34 and Table S6 in the Supporting Information).

### Local Temperature Determination

**Figure 4:**
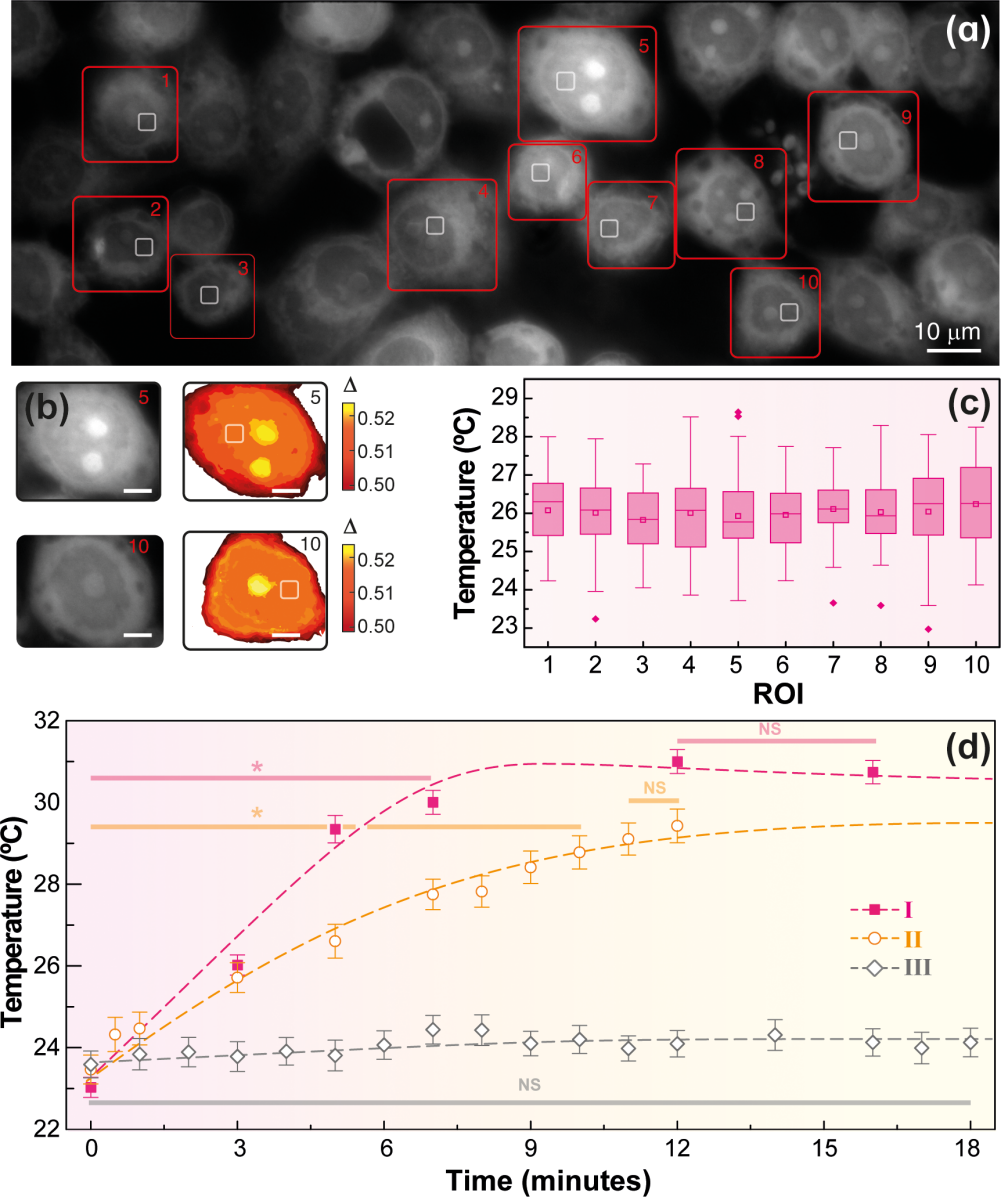
(a) Fluorescence image under 360 ± 20 nm illumination of living MDA-MB-468 cells in the Eu^3+^ channel for *t* = 3 min for experiment I. The big squares identify the 10 analyzed cells, and the small squares delimit the chosen ROI in each cell. (b) Magnification of the fluorescence images of cells 5 and 10 of panel (a), together with the corresponding Δ = *I*_1_/*I*_2_ map, taken as the pixel-by-pixel intensity ratio. The scale bars correspond to 10 μm, and the squares correspond to the selected ROIs. (c) Box plot diagrams of the 75 temperature readouts recorded in each ROI shown in (a). The one-way ANOVA analysis showed no statistically significant differences, meaning the average temperature was similar for all ROIs. (d) Transient temperature of living MDA-MB-468 cells during exposure to an external AFM recorded in experiments I, II, and III. Significance label, NS, no significance; *p ≤ 0.05; dashed lines are guides for the eyes.

Figure 4a presents an illustrative intensity image of MDA-MB-468 cells internalized with the dual heater–thermometer core@shell NPs recorded in the Eu^3+^ channel after 3 min of being irradiated by an external AMF (*Hf* = 2.4 × 10^9^ A·m^–1^·s^–1^). Analogous images were obtained in the Sm^3+^ channel (Figure S37 in the Supporting Information). To illustrate the intracellular temperature determination procedure, we selected 10 cells and in each one identified a region of interest (ROI) of 3 × 3 μm^2^ (small squares in Figure 4a). Some of the cells (e.g., those labeled as 5, 8, 9, and 10) presented brighter spots, which were ascribed to the nucleolus, as previously discussed.^35^Figure 4b presents a magnification of the cells labeled as 5 and 10, together with the corresponding Δ maps calculated by the procedure described in Materials and Methods (Figures S38, S39 and Table S7 in the Supporting Information). The intracellular temperature was then calculated using the calibration straight line presented in Figure 2b. The corresponding box plot diagrams for the ROIs (each one in a distinct cell) are shown in Figure 4c. The ANOVA analysis of the temperature readouts (see details in Materials and Methods) allows us to conclude that there are no differences between the mean temperature values recorded in the distinct ROIs of the distinct cells during exposure to the external AMF (within the uncertainty of the measurements, SD). An analogous concordance of the mean temperature values between the distinct ROIs is also observed in experiments II and III (Figure S40 in the Supporting Information).

The transient heating curves corresponding to the intracellular temperature variation as a function of the exposure time to the AMF (*Hf* = 2.4 × 10^9^ A·m^–1^·s^–1^) are represented in Figure 4d for experiments I, II, and III. At *t* = 0 s (with the AMF off), the temperatures measured by the dual heater–thermometer core@shell NPs (experiment I) or the single Sm^3+^/Eu^3+^-bearing thermometric nanomicelles (experiments II and III), 23.0 ± 0.4, 23.5 ± 0.7, and 23.6 ± 0.6 °C, respectively, are concordant with the temperature of the cell culture. Depending on the heater-to-thermometer distance (experiments I and II) and on the location of the thermal probes (experiments II and III), distinct transient curves were observed. In experiment I, there is a sudden temperature increase with a sharp slope within the first 5 min of AMF exposure, after which a plateau is reached (interpreted as a sign of equilibrium, with heat dissipation to the surrounding medium^34^). At *t* = 12 min the temperature is 31.0 ± 0.6 °C, which corresponds to an increase of 8.0 ± 0.5 °C with respect to the initial temperature. However, in experiment II (where the nanothermometers are not chemically linked to the nanoheaters) the temperature reaches 29.4 ± 0.8 °C at the same instant, corresponding to a temperature increase of 5.9 ± 0.8 °C. Moreover, it should be stressed that in this last experiment the temperature dynamics did not show a sudden initial increase with a sharp slope.

Regarding the relative positions of the magnetic heaters and the Sm^3+^/Eu^3+^-bearing thermometric nanomicelles, given that they both had the same surface composition (poly(ethylene glycol)), similar internalization pathways could be expected, as well as a short distance between each other. Indeed, colocalization experiments using magnetic heaters marked with RhB and nanomicelles marked with a cyanine dye (Dy-647P1) showed the expected short distance between each other, and a large proportion of nanomicelles were within touching distance of the nanoheaters (Figures S35, S36 in the Supporting Information). Depending on the proximity of the nanomicelles to the nanoheaters, the temperature of the nanomicelles also increases during AMF application, although to a lesser degree than on the surface of the MNPs. This is one of the basic fundamentals of the local hyperthermia hypothesis. It explains the lower maximum reached and the slower dynamics of the temperature increment measured by the single Sm^3+^/Eu^3+^-bearing thermometric nanomicelles in experiment II, relative to the readouts of the dual core@shell NPs in experiment I, given that they are not in direct contact with the magnetic nanoheater core. To estimate the overall temperature increase of the cell, we analyzed the temperature readouts in experiment III. The microscope images of the cell cultures showed that the luminescent probes are essentially located on the exterior of the cell membrane. Surprisingly, we realized that the average temperature remained constant at ∼24 °C during AMF application (experiment III, Figure 4d). Finally, we noticed that the temperature increment values recorded in experiments I and II were comparable to the values obtained by Clerc *etal*.^49^ (Δ*T* = 14.1 ± 1.4 °C) on MNPs encapsulated in lysosomes inside the cells, considering that the *Hf* of the AMF applied in their report (*Hf* = 12.7 × 10^9^ A·m^–1^·s^–1^, *H* = 53 mT, *f* = 300 kHz) was five times higher than what we used here. In this report, as mentioned above, the temperature measurement at a distance of 7 nm from the MNP surface was followed by the single emission of a cyanine dye.

### Hyperthermia Effects on Cell Death

**Figure 5:**
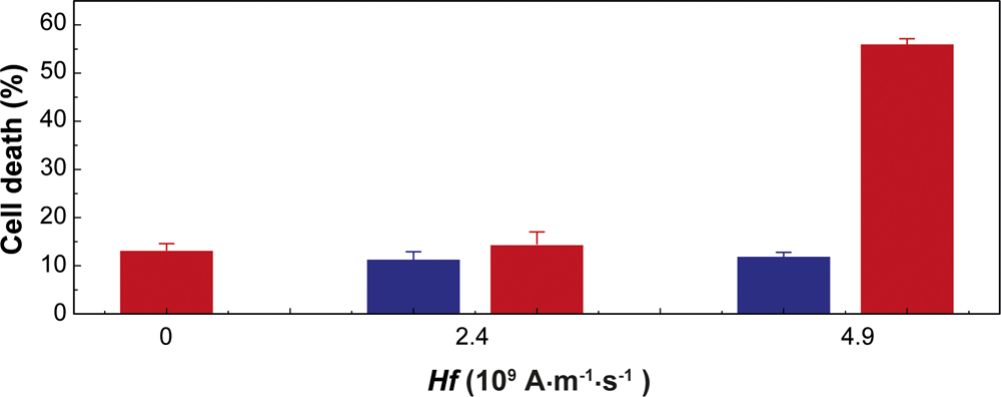
Annexin V^+^ cell death assays of cells (>10,000 in each experiment) without MNPs (blue) or cells with internalized MNPs (red), 24 h after exposure to a 30 min long AMF of the same frequency and intensity as the one used in the temperature imaging (*Hf* = 2.4 × 10^9^ A·m^–1^·s^–1^) or close to the human health safety limit (4.9 × 10^9^ A·m^–1^·s^–1^).

A temperature increase of just a few degrees above physiological values can cause protein unfolding, entanglement, and unspecific aggregation that would trigger a heat shock response to revert the damage,^62^ in addition to causing alterations to membrane stability and reactive oxygen species (ROS) generation.^63−66^ The generated temperature increments of 8 °C obtained in MDA-MB-468 cells internalized with the dual heater–thermometer core@shell NPs during exposure to an external AMF could presumably be high enough to produce irreversible damage to the surrounding tissue, thereby leading to cell death. We, therefore, performed annexin V assays in cells containing internalized MNPs after exposure to an AMF with a power similar to that used in the temperature imaging experiments. The results are shown in Figure 5. It can consequently be inferred that the aforementioned local temperature increments are sufficient to cause cell death, although to a very low degree (from 11.1% with AMF without NPs and 13.0% with NPs and no AMF to 14.2% with NPs and AMF). Despite this work representing a proof of concept, we must stress that the field parameters used in these experiments (*Hf* = 2.4 × 10^9^ A·m^–1^·s^–1^, *H* = 30 mT, *f* = 100 kHz) are still well within the health safety limits for human use, which is usually established at *Hf* = 5 × 10^9^ A·m^–1^·s^–1^. When we increased the *Hf* values close to this limit (4.9 × 10^9^ A·m^–1^·s^–1^, *H* = 60 mT, *f* = 100 kHz), the specific absorption rate (SAR) value increased from 45 to 150 W·g(Fe_2_O_3_)^−1^ and the cell death ratio subsequently increased to 60%, a value already relevant for the final goal of therapeutic performance. The efficacy of local hyperthermia could be increased when NPs are targeted to specific cell sites with the highest thermal sensitivity. One possible cell site especially sensitive to temperature increments is the nucleolus,^62,67^ which performs essential cell functions and where our heater nanoparticles spontaneously accumulate without any targeting agent. Another sensitive organelle could be the mitochondrion,^68^ which is directly involved in ATP and ROS generation, as well as in the apoptotic programs. Another factor to consider is the protective response to heat shock by heat shock proteins and how this can be affected by local heating at precise cell sites. Indeed, it has been reported that isolated mitochondria become uncoupled at temperatures above 45 °C,^66^ and we have recently observed, in intact cells as well as in isolated mitochondria, that temperatures above 43 °C (that is, 6 °C above the physiological value) cause the destabilization of mitochondrial complexes and supercomplexes, leading to a compromised respiratory function.^69^ Thus, the detected colocalization of MNPs with the mitochondria and nucleolus could help to reach the level of observed cell death.

Considering the total cell count of 170,000 in the culture, the amount of internalized NPs per cell was approximately 17,600 NPs/cell; the total amount of Fe_2_O_3_ per cell, estimated by ICP-OES, was 0.28 × 10^–12^, and the total heating power per cell was 7.4 × 10^–12^ W (assuming the calculated SAR as 45 W·g(Fe_2_O_3_)^−1^). Thus, considering a cell heat capacity of 3.22 J·K^–1^·g^–1^, as reported for red cells;^32^ a cell weight of 3.2 × 10^–9^ g, estimated from the cell size according to a confocal microscope; and a cell density of 1 g·mL^–1^, the accumulated heat in the cell generated by hyperthermia after 5 min of exposure to an AMF would produce a temperature increase of only 0.2 °C, in the ideal case in which no heat was dissipated to the medium (i.e., in the case of isolated or compact packed cells). We nevertheless observed that this treatment already can cause a small degree of cell death when compared with cells treated with AMF but without NPs (14.2 *vs* 11%).

Even considering stronger treatment with a SAR of 145 W·g^–1^ obtained with fields within the safety limit, the generated heat would still be too low to produce significant overall heating of the cell, with a temperature increase (using the same calculations as before) of 0.6 °C. However, in this case, we observed that the cell death effect becomes very relevant (close to 60%). Thus, both the experiments and the theory indicate that hyperthermia under our experimental conditions is too scarce to raise the temperature of the whole cell, and therefore the cause of the cell death (through apoptosis or other mechanisms) could be the considerable temperature gradients produced within the vicinity of the magnetic nanoheaters.

### Conclusions

In summary, our *in vitro* results indicate that the local hyperthermia therapy approach suggested as an improved alternative to the current overall heating with hyperthermia is feasible. Other experimental reports support this hypothesis. For instance, Rodriguez-Luccioni *et al*.^70^ reported a decrease in cell viability and the activation of apoptotic pathways after magnetic hyperthermia. They also observed increased membrane permeability that enhanced the effect of anticancer drugs such as CisPt^71^ and lipid and protein denaturation.^72^ Reliable luminescent thermometers were developed in the form of core@shell heater–thermometer NPs (comprising a γ-Fe_2_O_3@_ core coated with a polymeric shell embedded with Sm^3+^- and Eu^3+^-bearing complexes), thereby allowing the temperature to be determined irrespective of the surrounding media. These NPs registered an intracellular temperature increase of 8 degrees upon exposure to an AMF, showing a steep increase within the first 5 min, followed by a constant temperature readout. The rational design of the control experiments, according to which the distance between the heat source and the thermometric probes was increased using a dual-particle approach, pinpointed the relevance of local heating in the cell death processes. When the Sm^3+^/Eu^3+^-bearing thermometric nanomicelles are not chemically linked to the γ-Fe_2_O_3_ MNPs, the intracellular temperature dynamics is distinct (with a smaller temperature increment and a lower rate of increase). This illustrates the limitations of dual-particle approaches for precise intracellular temperature readouts, given that the temperature determination is critically dependent on the distance of the thermometer–MNPs, and consequently the measured temperature corresponds to an average value instead of a precise one ascribed to a particular heater–thermometer distance (as in the case of the single-particle approach). The cell membrane temperature readouts virtually show no change for similar exposures to the AMF (a result corroborated by a conventional thermometric probe placed in the culture medium). We can conclude that the combination of heater/thermometer and nanomicelle temperature probes can be very useful in determining internal cell thermal phenomena leading to cell death. Moreover, these results suggest that the current theoretical modeling of heat transfer in cells is insufficient to explain the local temperature differences found within a cell in magnetic hyperthermia.

## Methods

### Materials

All the details of the synthesis and characterization of copolymers, ligands, Sm^3+^ and Eu^3+^ complexes, MNPs, dual heater–thermometer NPs, and single Sm^3+^/Eu^3+^-bearing thermometric nanomicelles are given in Section II in the Supporting Information.

### Synthesis and Characterizations

The synthesis of the copolymer used for the preparation of the dual heater–thermometer core@shell NPs was carried out by RAFT polymerization. First, the P(MPEGA-st-PEGMA) block was synthesized from 2-cyano-2-propyl dodecyl trithiocarbonate (CTA) poly(ethylene glycol) methyl ether methacrylate (MPEGMA) and poly(ethylene glycol) methacrylate (PEGMA) monomers, using 2,2′-azobis(2-methylpropionitrile) (AIBN) as the initiator. Then, P(MPEGMA-st-PEGMA) was used as the macro-CTA for the polymerization of 4-vinylpyridine (4VP) and 4′-(4-((4-vinylbenzyl)oxy)phenyl)-2,2’:6′,2″-terpyridine (VBPTpy) to obtain the P(MPEGMA-st-PEGMA)-*b*-P(4VP-*b*-VBPTpy) amphiphilic copolymer (BCP1). Subsequently, the copolymer was functionalized with Sm(BNPD)_3_·2H_2_O and Eu(BNPD)_3_·2H_2_O complexes (BNPD stands for 1-([1,1′-biphenyl]-4-yl)-3-(naphthalen-2-yl)propane-1,3-dione) by reaction with the terpyridine residues of the polymer in toluene, evaporation of the solvent, and dissolution in dimethyl sulfoxide (DMSO). The copolymer used in the preparation of the single Sm^3+^/Eu^3+^-bearing thermometric nanomicelles was prepared similarly by reaction of CTA, poly(ethylene glycol) methyl ether acrylate (MPEGA), poly(ethylene glycol) acrylate (PEGA), and AIBN to obtain the P(MPEGA-st-PEGA) block that was then used as the macro-CTA for the polymerization of cholesteryl acrylate (CholA) and PhenA to obtain the final P(MPEGA-st-PEGA)-*b*-P(CholA-st-PhenA) amphiphilic copolymer (BCP2). The copolymer was then functionalized with the Ln(BNPD)_3_·2H_2_O complexes by reaction with the PhenA residues of the polymer in toluene, evaporation of the solvent, and dissolution in tetrahydrofuran (THF).

γ-Fe_2_O_3_ NPs synthesized by co-precipitation and hydrothermal treatment^73^ were redispersed in methanol and mixed with the DMSO solution of the polymer (BCP1, Section II in the Supporting Information), while water was slowly added to the mixture. Coated NPs were then collected in a magnetic column and redispersed in water to obtain a stable suspension of dual heater–thermometer core@shell NPs. The water suspensions of the single Sm^3+^/Eu^3+^-bearing thermometric nanomicelles were prepared by slow addition of water to the THF solutions of the lanthanide-doped copolymer (BCP2, Section II in the Supporting Information), and then the THF was removed by dialysis to obtain stable micellar dispersions.

The collagen gels used for determining the thermometric response of the dual heater–thermometer core@shell NPs were prepared by mixing 360 μL of rat tail collagen type I (>5 mg, First Link UK Ltd.), 100 μL of the NP suspension at a concentration of 1.6 mg/mL, 140 μL of HBSS (Hank’s Balanced Salt Solution), and 150 μL of 0.25 M NaOH. The mixture was deposited on a 35 mm glass-bottom dish (Ibidi) that was placed in the thermostatic holder fixed on the microscope stage. The temperature was controlled by a semiconductor sensor (Neoptix Reflex, accuracy ±0.8 °C according to the manufacturer).

### Cell Cultures

Maintenance of the MDA-MB-468 cells, NP seeding, cell death, internalization, and NP localization assays were carried out according to standard procedures and are described in detail in Section VIII in the Supporting Information.

### Temperature Imaging of Cell Cultures

Fluorescence images were recorded under 360 ± 20 nm illumination in a microscope (Leica DMI3000B). The images were captured simultaneously in the Sm^3+^ and Eu^3+^ channels by an Orca 4.0 camera (Hamamatsu), after splitting the incident light into two beams (Figure 2a). Each beam was filtered through a band-pass filter, 610 ± 20 nm for the Eu^3+^^5^D_0_ → ^7^F_2_ transition, *I*_2_, and 650 ± 40 nm for the Sm^3+^^4^G_5/2_ → ^6^H_9/2_ transition, *I*_1_. The optical images were converted into temperature maps using a dedicated MatLab routine. First, the routine splits the as-recorded grayscale image of the CMOS camera into the Eu^3+^ and Sm^3+^ channels and then determines the pixel-by-pixel ratiometric image according to Δ = *I*_1_/*I*_2_ (illustrative examples in Figure 4b). The temperature is then calculated using the intensity-to-temperature calibration straight line, as Figure 2b illustrates for the dual heater–thermometer core@shell NPs. For the Sm^3+^/Eu^3+^-bearing thermometric nanomicelles, the calibration was obtained based on Figure 2b and the information presented in Figure S17 in the Supporting Information.

### Magnetic Heating Equipment

In-house magnetic heating sources were used consisting of a signal generator, a power amplifier, and an RCL circuit. The L element consists of a ferrite electromagnet with Litz wire windings. The ferrite nucleus for aqueous suspension experiments has a rectangular shape, with a 1.1 cm gap and 2.1 cm^2^ cross-section, while the nucleus for cell cultures has a toroid shape with a 0.5 mm gap (Section III in the Supporting Information).

### Statistics

OriginLab software was used to perform all the statistical analyses. The box plot charts are used to represent the statistics of the intracellular temperature readouts. Box limits indicate the range of the central 50% of the data, and the central line marks the median value. The vertical lines extending from each box display the remaining data range, and the diamond dots indicate outliers. The differences between intracellular temperature readouts were analyzed using a one-way analysis of variance (ANOVA). The analysis was performed using 10, 6, and 8 ROIs (one region per cell), respectively for experiments I, II, and III. For each ROI, *n* = 75 randomly sampled positions were considered, corresponding to a total of 750, 450, and 600 points, for experiments I, II, and III, respectively. Data are shown as the mean ± SD. In each ROI, the distribution of the temperatures is well described by a Gaussian profile (Figure S38 in the Supporting Information), meaning that all the temperature readouts follow normal distributions. The temperature distributions in the distinct ROIs (distinct cells) at distinct time instants (for the same experiment) and in distinct experiments (transient curves) were analyzed using the F-test to evaluate the hypothesis that the temperature readouts (normal populations) present identical mean values; *p values ≤ 0.05 are considered to be statistically significant.

## Supporting Information Available

The controversy about intracellular local temperature increments; materials and methods; experimental setup for magnetic-induced heating and optical microscope temperature imaging; calibration of the thermal probes, relative thermal sensitivity, and temperature uncertainty; temperature imaging of living cells; cell cultures; intracellular temperature evolution over time during exposure to an AMF (PDF)
