# Supplementary material for: Local Temperature Increments and Induced Cell Death in Intracellular Magnetic Hyperthermia
Source: ACS Nano. 2023 Mar 20;17(7):6822–32. doi: 10.1021/acsnano.3c00388 (PMC10100554; doi:10.1021/acsnano.3c00388)
Supplement: Supplementary file 1 — nn3c00388_si_001.pdf [file nn3c00388_si_001.pdf]

SUPPLEMENTARY INFORMATION

Local temperature increments and induced cell death in intracellular magnetic hyperthermia

Yuanyu Gu[1,2], Rafael Piñol[1], Raquel Moreno-Loshuertos[3], Carlos D.S. Brites[4], Justyna Zeler[4,5], Abelardo Martínez[6], Guillaume Maurin-Pasturel[1], Patricio Fernández-Silva[3], Joaquín Marco-Brualla[3], Pedro Téllez[1], Rafael Cases[1], Rafael Navarro Belsué[1], Debora Bonvin[7], Luís D. Carlos[4], and Angel Millán[1]**

[1] INMA, Institute of Nanoscience and Materials of Aragon, CSIC-University of Zaragoza, Zaragoza, C/ Pedro Cerbuna 12, 50009 Zaragoza, Spain

[2] School of Materials Science and Engineering. Nanjing Tech University, 210009, Nanjing PR China

[3] Department of Biochemistry and Molecular and Cellular Biology, and Institute for Biocomputation and Physics of Complex Systems, University of Zaragoza, C/ Pedro Cerbuna 12, 50009 Zaragoza, Spain

[4] Phantom-g, CICECO-Aveiro Institute of Materials, Department of Physics, University of Aveiro, Campus de Santiago, 3810-193 Aveiro, Portugal

[5] Faculty of Chemistry, University of Wroclaw, 14. F. Joliot-Curie Street, 50-383 Wroclaw, Poland

[6] Department of Power Electronics, I3A, University of Zaragoza, 50018 Zaragoza, Spain

[7] Powder Technology Laboratory, Institute of Materials, Ecole Polytechnique Fédérale de Lausanne, 1015 Lausanne, Switzerland

Contents

I. The controversy on local intracellular temperature increments 3
II. Materials and methods 9
Materials 9
Synthesis 9
Polymerizable auxiliary ligands.. 9
Block copolymers.. 10
γ-Fe_2O_3 nanoparticles 13
Ligands and complexes 13
Hydrophobic block polymer in the nanoparticles 15
Hydrophilic block polymer in the nanoparticles.. 15
Dual heater-thermometer core@shell nanoparticles 16
Core@shell magnetic nanoparticles 17
Rhodamine B labeled magnetic nanoparticles 17
Sm^{3+}/Eu^{3+}-bearing thermometric nanomicelles.. 17
Methods 18
Characterization of polymers 18
Chemical and physical characterization of the materials... 22
III. Setup for magnetic-induced-heating and optical temperature imaging 29
IV. Calibration of the molecular temperature probes 31
V. Relative thermal sensitivity and temperature uncertainty... 33
VI. Temperature imaging of living cells... 35
Magnetization measurements 36
Specific absorption rate (SAR) measurements 37
VII. Cell cultures.. 38
Cell internalization of the nanoparticles 38
Cytotoxicity analysis 38
TEM images of cell cultures... 39
Confocal microscope images.. 45
Lysosomes and mitochondria colocalization experiments 47
Colocalization of magnetic heaters and thermometric nanomicelles 49
Cell death in local intracellular hyperthermia 51
VIII. Intracellular temperature evolution over time during exposure to an AMF............ 52
References 56

I. The controversy on local intracellular temperature increments

Theoretical thermal analyses, based on the classical Fourier's laws of heat conduction (discarding a ballistic approach due to the short mean free path of the magnetic nanoheaters) predict the unfeasibility of creating significant temperature gradients by intracellular hyperthermia. In 2002, Rabin[1] performed an analysis of heat transfer on cell internalized magnetic nanoparticles (MNPs, heaters) approximating the cell interior as a water suspension. He estimated a temperature increment of 10^{-5} K (at steady-state) in a single uncoated iron NP with a diameter of 100 nm and heating power (quantified by the specific absorption rate, SAR) of 150 $W{\cdot}g^{-1}$. Analogous calculations on a single cell containing densely packed NPs resulted in a temperature increase of 0.1 K for a cell size of 25 µm. Furthermore, Rabin estimated that to achieve a temperature increase of 6 degrees necessary for cell death, with $1/30^{th}$ of the cell volume occupancy of MNPs, the tumor should have a minimum size of 1.1 µm.[1] Furthermore, the author anticipated that the cell membrane would have a negligible thermal isolating effect.

Later, Keblinski *et al.*[2] applied the diffusive heat equation to a single nanoheater with a heating power of 14 nW in tissue with thermal conductivity of κ=0.3 $W{\cdot}m^{-1}\ K^{-1}$, neglecting both the heat capacity of the NP and the thermal conductance of the interface. The authors estimated that the characteristic time of the transient heating process for a nanoheater (diameter of 130 nm) should be 100 ns and the temperature increment 0.06 K, in water suspension, at the steady-state. Moreover, they considered the effect of an ensemble of randomly dispersed 10^{15} nanoheaters per cubic meter, yielding a transient time of 1 ms, and a local temperature increase in a single NP of 1% of the global temperature increase. However, some authors are already questioning the validity of the Fourier diffusive heat equation as the width of heat sources is reduced.[3]

Kozissnik *et al.*[4] and Chiu-Lam & Rinaldi[5] estimated the accumulation of MNPs with SAR from 100 to 1000 W per gram of Fe_2O_3 in the tumor by considering the effects of tissue blood perfusion. According to these works, the only possibility to achieve a local temperature of 45 ºC is by the direct massive injection of MNPs in the tumor.

Several attempts have been made to measure the local temperature of MNPs in water suspensions. A representative account of measured local temperature

increments with respect to the structural and thermal characteristics of the MNPs and alternating magnetic field (AMF) features is given in Table S1. In a couple of examples of MNPs exposed to an AMF the measured temperature increments were negligible, as predicted by theory.[6, 7] For instance, Gupta *et al.*[6] employed fluorescent quantum dots (QDs) as temperature probes and found no difference between the temperature in the vicinity of rf-heated NPs and that in the bulk. Faure *et al.*[7] used a synchrotron technique to measure the internal temperature of MNPs embedded in a solid matrix and found no difference with the matrix. Later, some of the authors of the paper considered the technique complex and not representative of the behavior of the particles in solution, and they used catalytic thermometry to measure high surface temperatures that varied accordingly with the magnetic field intensity.[8] This is also the case for most of the local temperature determinations in solutions and cells. Some authors have questioned the validity of fluorescence dye temperature measurements,[9] which is the most typical thermometry system used in these studies, but non-optical methods yielded similar results (Table S1). These controversial reports have opened a debate about heat transfer phenomena at the nanoscale.[10] Moreover, the question of ultralow thermal conductivity and steep temperature gradients in the nanoscale is not exclusive to MNPs as it has been observed in other systems as laser-heated metal NPs, carbon nanotubes, and graphene embedded in a matrix or silicon nanowires, boosting the need for both experimental thermometry improvements and better theoretical understanding of the mechanisms of heat transfer across interfaces.[11]

Techniques to measure the local temperature of nanoheaters in water suspensions have evolved with the progress of luminescence nanothermometry. Although the detection is fluorescence emission, several thermo-dependent physical and chemical properties have been used for sensing. Early attempts were based on bond breaking or polymer transitions occurring above a certain temperature threshold. Thus, these methods only detect when the temperature of the MNPs rises above a threshold, not giving, therefore, the temperature of the NPs.

Polo-Corrales *et al.*[12] used MNPs coated with a thermosensitive polymer (poly(N-isopropylacrylamide), pNIPAM) with a critical transition at 35 ^{o}C and found that the temperature of the MNPs raised above this threshold value from

an initial temperate of 20 ºC under the action of an AMF. Using distinct techniques dissimilar results were found. Riedinger *et al.*[13] used thermosensitive azo-bonds and reported temperature increments above 45 degrees. Dias *et al*.[14] used the thermal decomposition of DNA strands to report temperature increments of 8.3 degrees.

Later, techniques based on temperature-dependent emission were developed, allowing *in situ* temperature determination. Small temperature increments (1.2 degrees) were measured by Shah *et al.*[15] using the temperature dependence of the emission intensity of Rhodamine B (RhB). Gupta *et al.*[6] followed the peak shift of QDs with temperature to report negligible local temperature increments, in good agreement with the theoretical predictions. It is well known that luminescence thermometry based on single emission intensity measurements might be affected by the intensity fluctuations of the excitation source and the concentration of emitters.[16]

Thus, lifetime thermometry emerged as a reliable approach. Freddi *et al.*[17] used the lifetime of the RhB to measure temperature increase values of 10 degrees. Later, 2020 Silva *et al.*[18] used the lifetime of the Green Fluorescence Protein (GFP) to obtain temperature increments of up to 57 ºC. In HeLa cells, Silva *et al.*[18] used an AMF (0.016 $Am^{-1}s^{-1}$, *f*=499 kHz) applied to PAA-coated Fe_3O_4 NPs, located mainly around the nucleus. The temperature in the cytoplasm was accessed using the GFP lifetime, calibrating the protein in the 18-29 ºC range (temperatures above this range were extrapolated, assuming a linear relationship between the lifetime and the temperature). They reported an astonishingly high increase of up to 55 degrees after 37 min field exposure. Moreover, as the probes were not placed at the heat source but at some distance from it, the local temperature of the MNP should be even higher. Nevertheless, it is challenging to draw conclusions from these experiments as the calibration curve was realized in a low-temperature range (18-29 ºC) and the temperatures above this range were calculated by extrapolation assuming a linear relationship between the GFP emission lifetime and the temperature. Furthermore, the SAR of the particles was not given in this report.

Ratiometric thermometry goes a step forward in reliability as it is independent of the light source intensity and the density of emitters and yields absolute temperatures. Resorting to this method, Dong *et al.*[19] used the upconverting Er^{3+}

emission of $NaYF_4$:(Yb^{3+}, Er^{3+}) NPs placed in a silica matrix. They observe an increase of about 20 degrees in the vicinity of MNPs (encapsulated in the same silica matrix),[19] nevertheless, this two-particle approach does not yield the temperature on the NPs, but that on the matrix at a given distance from the nanoheater.

Huang *et al.*[20] studied MNPs in water suspensions fixed to the cell membrane. A DyLight549 luminescent temperature dye attached to the MNPs by streptavidin linkers was used to probe the temperature. In this report, a temperature increment of 15 °C was found in DMSA-coated Mn-ferrite NPs attached to the membrane of HEK 293 cells after applying an AMF for 15 s. The temperature increase measured in the cell membranes (15 ºC) is surprisingly higher than that obtained in water suspensions (4 ºC), especially when considering that the heating power of the MNPs usually decreases with the restrained mobility of the particles (due to a lower contribution of the Brownian relaxation to the heating). The high-temperature increments found in MNPs attached to the cell membranes could be due to a high particle accumulation on the membrane and are not representative of the cell cytoplasm environment.

Our contribution to local hyperthermia is based on a single-particle approach that used molecular probes situated on the surface of MNPs.[21] The ratiometric molecular temperature probes that consisted of Eu^{3+}- and Tb^{3+}-containing lanthanide complexes showed a temperature-dependent decrease of the emission intensity with the temperature. We measured temperature increments of about 2 degrees in the MNPs with respect to the bulk temperature.[21]

More recently, Clerc *et al.*[22] used the single emission of a cyanine dye (DY549) to measure the temperature increase at a distance of 7 nm from the surface of MNPs internalized life cells when they were submitted to magnetic hyperthermia treatment. The MNPs were encapsulated in the lysosomes and the *Hf* of the applied magnetic field was $1.2x10^{10}$ $Am^{-1}s^{-1}$ (thus, almost twice the safety limit value Hf=$5x10^{9}$ $Am^{-1}s^{-1}$). After ~7 min of exposure to the external AMF, a temperature increment of 14.1±1.4 °C was detected by the dye thermometer, without any significant increase at the outside of the lysosomes membrane.[22]

Obviously, the temperature increase in the MNPs will strongly depend on the heat power. Unfortunately, SAR values are not reported systematically. However, most of the temperature increments extracted from Table S1 (reporting the

corresponding SAR value) are well fitted to a linear increase of the SAR values with the temperature (Figure S1), despite the very different thermometric methodologies adopted for the temperature measurements.

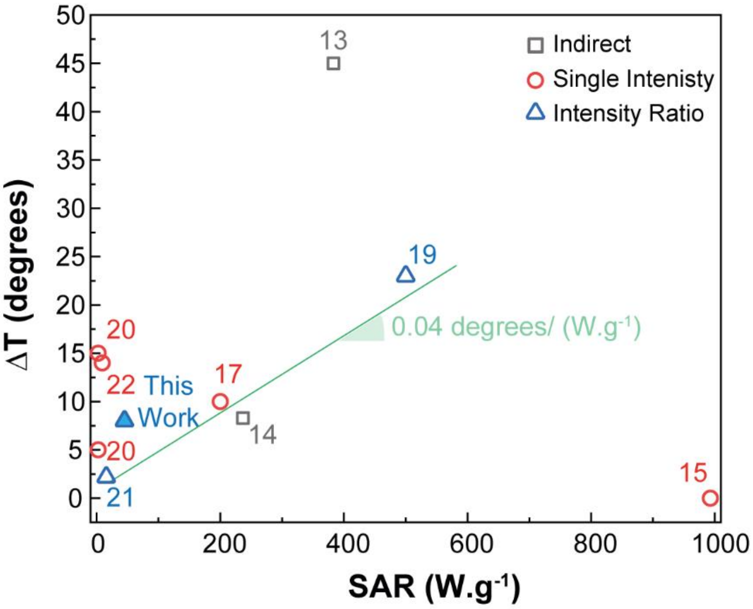

Figure S1. Local temperature increment values of MNPs in water suspensions under exposure to an AMF as a function of the SAR values for the examples gathered in Table S1. The line is a guide for the eyes and the numbers correspond to the references listed in Table S1.

Table S1. Reported temperature increments in magnetic hyperthermia measured by luminescence thermometry.

MNPs	Coating	Medium	Size (nm)	D_H (nm)	Conc. ($g(Fe_2O_3)\cdot L^{-1}$)	SAR (W $g(Fe_2O_3)^{-1}$)	H ($kA\cdot m^{-1}$)	f (kHz)	Exposure time (min)	Temperature probe	Detected signal	Temperature Increase	Ref.
**INDIRECT METHODS**													
Fe_2O_3	PEG8000	Water	15		0.027	383*	13.6	335	60	Azo-bond breaking	Intensity	44	13
Fe_3O_4	PMAO	Water	12	60	0.58	236.5	20	835	33	DNA denaturation	Intensity	8.3	14
**SINGLE INTENSITY**													
Iron oxide	copolymer	Water		100	10	-	38.4	233	10	Critical transition	Intensity	>15	12
Fe_3O_4	SiO_2	Water	10		<<1	-	0.472	820		QD	Peak shift	negligible	6
γ-Fe_2O_3		Water	22	53		993	38.2	430	20	RhB	Intensity	1.2	15
Fe_3O_4	PSS	Water		124	30	200	25.5	168	1.7	RhB	Lifetime	10	17
Fe_3O_4	PAA	Cell	8.1	40	28**	-	16	499	20	GFP	Lifetime	<57	18
Mn-Fe_3O_4	Water	Water	6		20	2.5	1	40000		DyLight549	Intensity	5	20
Mn-Fe_3O_4	DMSA-streptavidin	Cell Membrane	6	6-8		2.5	1	40000	2.5	DyLight549	Intensity	15	20
Fe_3O_4	PEG-Amine	Cell	8.7	40	0.023	9.1	32	275	7	DyLight549	Intensity	14	22
**INTENSITY RATIO**													
Fe_3O_4	SiO_2	Water	17		0.194	500	20-24	375	5	$NaYF_4$: Yb^{3+}/Er^{3+}	Intensity Ratio	23	19
γ-Fe_2O_3	P4VP-P(PEGA)	Water	10	48	6.2	15.6	18.3	98	2.5	$[Tb,Eu](btfa)_3$	Intensity Ratio	2.2	21
γ-Fe_2O_3	P4VP-P(PEGA)	Cell	20	50		45	24	100	5	$[Eu,Sm)BNPD_3VBPTpy$	Intensity Ratio	8	This work

* Interpolated from the data presented by Tong *et al.*[23] where similar cubic iron oxide nanoparticles (with the same size and synthesized through the same method) had a SAR of 265 W $g(Fe_2O_3)^{-1}$ at H=9.35 $kA\cdot m^{-1}$ and 597 W $g(Fe_2O_3)^{-1}$ at H=20.7 $kA\cdot m^{-1}$, both at 325 kHz.
** 10^{-12} g Fe_2O_3 per cell.

II. Materials and methods

Materials

Europium(III) chloride hexahydrate ($EuCl_3(H_2O)_6$, 99.99%), samarium(III) chloride hexahydrate ($SmCl_3(H_2O)_6$, 99.99%), Acryloyl Chloride, Hydroxybenzaldehide (98%), 2-Acetonaphtone (99%), Sodium Hydride (60% dispersion oil), 2-acetypyridine (99%), Triethylamine (Et_3N), Methyl 4-phenylbenzoate (98%) 4-Vinylbenzyl chloride (90%) Sodium Hydride (60% dispersion oil), %), Cholesterol (99%), 4-Methyl-1,10-phenanthroline (MPhen), and 4´-(4-methylphenyl)-2,2´:6´,2´´-terpyridine (MPhTpy, 98%) were all products of Sigma Aldrich and were used as received without further purification The synthesis of 1,10-phenanthroline-4-carboxyaldehyde,[24] 4-(hydroxymethyl)-1,10-phenantroline,[25] 2,2´:6´.2´-terpirdin -4-yl) phenol (TpyOH)[26] and Cholesteryl Acrylate (CholA)[27] was accomplished following the straightforward methods reported in the literature with slight modifications.

Synthesis

Polymerizable auxiliary ligands

4 –acryloyloxymethyl-1,10-phenanroline (PhenA)

Acryloyl chloride (0.3 mL, 4.2 mmol) was added dropwise to an ice-water bath-cooled mixture of 4-(hydroxymethyl)-1,10-phenantroline (735.8 mg, 3.5 mmol) and trietylamine (0.6 mL, 4.2 mmol) and a few grains of 2,6-di-tert-butyl-4-methylphenol (BHT) in dry chloroform (35 mL) under argon atmosphere. After addition, the reaction mixture was stirred at room temperature for 24 hours. The reaction mixture was diluted with chloroform (15 mL), washed with a solution of sodium bicarbonate (5%, 25 mL), water (25 mL), dried over anhydrous sodium sulfate, and the solvent evaporated by a rotary evaporator. The compound was purified by flash column chromatography on silica gel using dichloromethane/methanol (9:1) as eluent. After drying under vacuum at 35 ºC, 0.75 g (2.8 mmol, Yield: 80%) of a white solid was obtained.

^{1}H-NMR (400 MHz, CDCl3) δ (ppm): 9.22 (,dd, J1: 4.33 Hz, J2: 1.74 Hz,1H, Phen), 9.20 (d, J: 4.49 Hz, 1H, Phen), 8.28 (dd, J1: 8.09 Hz, J2: 1.74 Hz, 1H, Phen), 7.99 (d, J: 9.10 Hz, 1H, Phen), 7.99 (d, J: 9.10 Hz, 1H, Phen), 7.70 (d, J: 4.50 Hz, 1H, Phen), 7.66 (dd, J1: 8.07 Hz, J2: 4.33 Hz, 1H, Phen), 6.53 (dd, J_1: 17.33 Hz, J_2: 1.29 Hz, 1H, -CH=CH_2), 6.25 (dd, J_1: 17.33 Hz, J_2: 10.45 Hz, 1H, -CH=CH_2), 5.95 (dd, J_1:

10.45 Hz, J_2: 1.33 Hz, 1H, -CH=CH_2), 5.76 (s, 2H, -CH_2). ^{13}C-NMR (100 MHz, $CDCl_3$) δ (ppm): 165.6, 150.5, 150.0, 145.9, 145.8, 141.3, 136.2, 132.1, 128.3, 127.6, 127.1, 126.3, 123.3, 121.9, 121.5, 62.6.

4'-(4-((4-vinylbenzyl)oxy)phenyl)-2,2':6',2''-terpyridine (VBPTpy)

TpyOH (3.25 , 10 mmol) and 4-vinylbenzyl chloride (2.35mL, 15 mmol) were added consecutively to a suspension of ground KOH (5.28 g, 80 mmol) in Dimethylsulfoxide (DMSO, 50 mL) under inert atmosphere. The reaction mixture was then heated at 60 ºC for 20 h under stirring. The resultant reddish-brown mixture was poured over distilled water (150 mL) and cooled at 4 ºC in the refrigerator overnight. The obtained pale orange solid was collected by filtration washed with water (2×50 mL) and dried under vacuum at 40 ºC. The crude compound was purified twice by recrystallization from hot ethanol and finally by flash column chromatography on silica gel using dichloromethane/methanol (19:1) as eluent to give 2.76 g (6.25 mmol, yield 62.5 %) of a pale orange powder.

^{1}H-NMR (400 MHz, $CDCl_3$) δ (ppm): 8.79-8.72 (m, 4H, tpy), 8.70 (d, 2H, J:7.96 Hz, tpy), 7.95-7.85 (m, 4H, Ar), 7.49-7.40 (m, 2H, Ar), 7.37 (ddd, 2H, J1:7.45 Hz, J2:4.85, J3: 1.15 Hz, 2H, tpy), 7.10 (d, 2H, J:8.85 Hz, Ar), 6.74 (dd, 1H, J1:17.61 Hz, J2:10.90 Hz, -CH=CH_2), 5.76 (dd, 1H, J1:17.61 Hz, J2:0.79 Hz, -CH=CH_2), 5.27 (dd, 1H, J1:10.90 Hz, J2:0.74 Hz, -CH=CH_2), 5.12 (s, 2H, -CH_2-). ^{13}C-NMR (400 MHz, $CDCl_3$) δ (ppm): 159.6, 156.0, 155.4, 149.7, 148.7, 137.4, 137.1, 136.3, 136.1, 130.9, 128.5, 127.7, 126.4, 123.8, 121.5, 118.4, 115.2, 114.1, 69.8.

Block copolymers

2,2′-Azobis(2-methylpropionitrile) (AIBN, Acros Organics, 98%) was recrystallized twice from ethanol. 2-cyano-2-propyl dodecyl trithiocarbonate (Aldrich, 97%, RAFT chain transfer agent) was used as received. Poly(ethylene glycol) methyl ether acrylate (MPEGA) (Mn: 480 Da, Aldrich) and poly(ethylene glycol) methyl ether methacrylate (MPEGMA) (Mn: 475 Da, Aldrich) were passed through a column of aluminum oxide to remove the inhibitors. Polyethylenglicol acrylate (PEGA, Mn: 468 D, calculated by ^{1}H-NMR spectroscopy) was synthesized from polyethylene glycol 400 (Aldrich) and purified according to the procedures previously described.[21] Polyethylenglicol methacrylate (PEGMA, Mn: 360Da, Aldrich) was purified following

the same protocol (Mn: 394 Da, calculated by ^{1}H-NMR spectroscopy after purification).

The block copolymers were prepared by sequential RAFT polymerization. All polymerization followed this general procedure: CTA or Macro-CTA, monomers, AIBN (initiator), and solvent were introduced into a Schlenk flask charged with a magnetic stirrer and sealed with a rubber septum. The solution was degassed by performing three freeze-vacuum-thaw cycles, backfilled filled with argon, and then immersed in a preheated oil bath. The polymerizations were stopped by cooling the reaction flask with a liquid opening to the atmosphere. The conversion was determined by ^{1}H-NMR (in $CDCl_3$) by comparison of the integration of the vinyl protons corresponding to the remaining monomers with the integration of polymer side chain signals of the protons next to the ester group of the PEG. To remove unreacted monomers, the Macro-CTAs P(MPEG(A)-st-PEG(M)A) or the final block copolymers, P(MPEGMA-st-PEGMA)-b-P(4VP-b-VBPTpy) (BCP1) and P(MPEGA-st-PEGA)-b-P(CholA-b-PhenA) (BCP2), were purified twice by dissolving in a small volume of tetrahydrofuran and precipitation in a large volume of cold diethyl ether.

The following amounts and conditions were applied for the polymerizations:

MacroCTA 1 P(MPEGMA-st-PEGMA). 2-cyano-2-propyl dodecyl trithiocarbonate (CTA) (178 mg, 0.5 mmol), MPEGMA (5.45 g, 10.5 mmol), PEGMA (1.78 g, 4.5 mmol), AIBN (16.4 mg, 0.1 mmol), Toluene (7.2 mmL). The polymerization was carried out at 70 ºC and stopped after 5 h. (Conversion = 93%). Mn (^{1}H-NMR): 12900 Da.

BCP1 P(MPEGMA-st-PEGMA)-b-P(4VP-b-VBPTpy). P(MPEGMA-st-PEGMA) (MacroCTA 1) (2.6 g, 0.5 mmol), 4VP (0.97 mL, 9 mmol), VBPTpy (441.5 mg, 1 mmol), AIBN (6.6 mg, 0.04 mmol), DMF (4.5 mL). The polymerization was carried out at 65ºC and stopped after 18 h. (Conversion 4-VP/VBTpy = 80%). Mn (^{1}H-NMR): 18400 Da.

MacroCTA 2 P(MPEGA-st-PEGA). 2-cyano-2-propyl dodecyl trithiocarbonate (CTA) (178 mg, 0.5 mmol), MPEGA (5.09 g, 10.5 mmol), PEGA (2.1 g, 4.5 mmol), AIBN (16.4 mg, 0.1 mmol), Toluene (7.2 mmL). The polymerization was carried out at 70 ºC and stopped after 5 h. (Conversion = 91%). Mn (^{1}H-NMR): 13200 Da.

BCP2 P(MPEGA-st-PEGA)-b-P(CholA-st-PhenA). P(MPEGA-st-PEGA) (MacroCTA 2) (2.6 g, 0.5 mmol), CholA (1.32 g, 3 mmol), PhenA (264.3 mg, 1 mmol), AIBN (6.6 mg, 0.04 mmol), 1,4-Dioxane (6.8 mL). The polymerization was carried out at 70 ºC

and stopped after 8 h. (Conversion CholA = 80%, PhenA = 70%) Mn (^{1}H-NMR). Mn (^{1}H-NMR): 19100 Da.

The structure of the final BCP1 and BCP2 polymers are shown in Figure S2 and Figure S3, respectively.

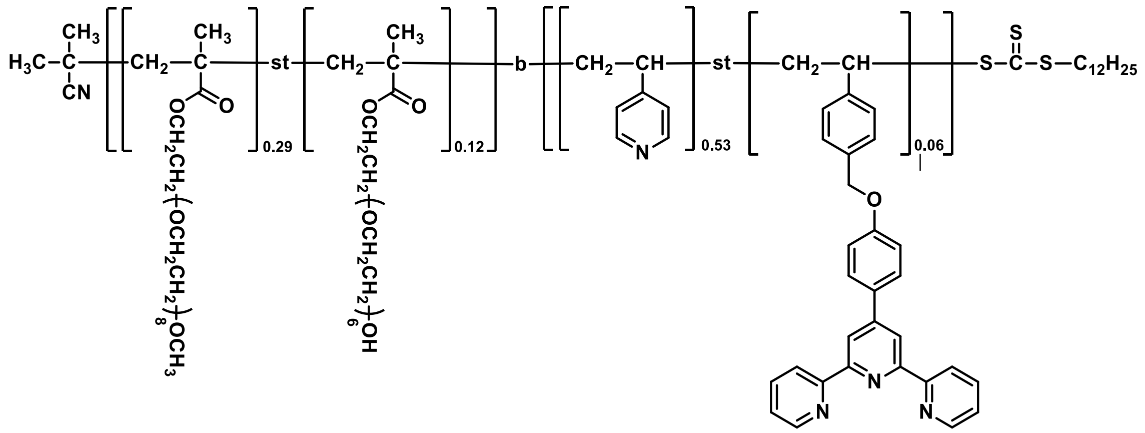

Figure S2. Structure of the block copolymer P(MPEGMA-st-PEGMA)-b-P(4VP-b-VBPTpy) (BCP1) used in the fabrication of the dual heater-thermometer core@shell NPs.

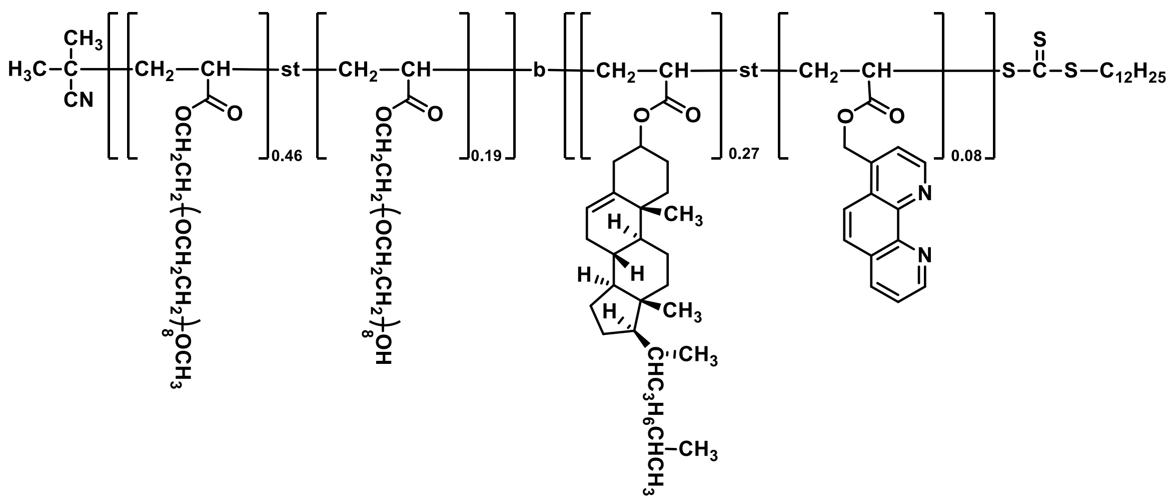

Figure S3. Structure of the block copolymer P(MPEGA-st-PEGA)-b-P(CholA-st-PhenA) (BCP2) used in the fabrication of the single Sm^{3+}/Eu^{3+}-bearing thermometric nanomicelles.

ɣ-Fe_2O_3 nanoparticles

ɣ-Fe_2O_3 MNPs were synthesized by co-precipitation in combination with a hydrothermal treatment performed at 120 ºC for 15 h following a protocol modified from Bonvin *et al.* described elsewhere.[28]

Ligands and complexes

1-([1,1'-biphenyl]-4-yl)-3-(naphthalen-2-yl)propane-1,3-dione (BNPD)

2-acetonaphtone (2.61 g, 15 mmol) dissolved in dimethoxyethane (DME, 15mL) was added dropwise under argon atmosphere to an ice-water cooled suspension of NaH (1.6, 40 mmol, 60% dispersion in mineral oil) in DME (130 mL). The mixture was allowed to reach room temperature and then a solution of 4-methyl biphenylcarboxylate (3.18 g, 15 mmol) in DME (25 mL) was added dropwise. The mixture was stirred at room temperature for 30 minutes and then heated at reflux for 2 days. After cooling at room temperature, the mixture was acidified by a careful addition of HCl 3M (375 mL). The crude product was extracted with CH_2Cl_2 (250 mL), the organic phase was dried over anhydrous $MgSO_4$ filtered through a pad of silica gel, and solvent, evaporated under vacuum. The crude product was washed with hot methanol (40 mL) and finally purified by several recrystallizations in acetone to yield 2.63 g (8.1mmol, Yield: 40%) of a faint yellow solid.

^{1}H-NMR($CDCl_3$, 400 MHz): 8.57 (s, 1H), 8.12 (d, J: 8.58 Hz, 2H), 8.05 (dd, J: 8.63 Hz, J: 1.75 Hz, 1H), 8.00 (dd, J: 7.57 Hz, J: 1.26 Hz, 1H), 7.94 (d, J: 8.70 Hz, 1H), 7.90 (dd, J: 8.85 Hz, J: 1.15 Hz, 1H), 7.74 (d, J: 8.58 Hz, 2H), 7.67 (dd, J: 8.27 Hz, J: 1.26 Hz, 1H), 7.64-7.54 (m, 2H), 7.50 (t, J: 7.41 Hz, 2H), 7.45-7.37 (m, 1H), 7.52 (s, 1H). ^{13}C-NMR ($CDCl_3$, 100 MHz): 185.46, 185.26, 145.20, 139.91, 135.30, 134.29, 132.79, 132.74, 129.36, 128.94, 128.47, 128.32, 128.16, 128.13, 127.77, 127.75, 127.31, 127.21, 126.79, 123.25, 93.42. FTIR (cm^{-1}): 3053, 1597, 1573, 1516, 1496, 1445, 1428, 1298, 1251, 791, 761, 729, 692.

$Ln(BNPD)_3(H_2O)_2$ complexes

To a stirred suspension of BNPD (0.52 g, 1.5 mmol) in EtOH (32 mL) heated at 70 ºC, NaOH (1M, 1.5 mL) and a solution of the corresponding $LnCl_3(H_2O)_6$ (0.5 mmol) in water (4.8 mL) were successively added. The mixture was heated at 100 ºC for 1 hour, cooled down to 80 ºC, and stirred for 18 hours. Water (75 mL) was added and the mixture was heated for 30 additional minutes. After cooling at room

temperature, the complex was collected by filtration and washed with water, warm EtOH (50 ºC), and dried in vacuo (35 ºC).

Eu(BNPD)$_3$(H_2O)$_2$. Yield 520 mg (84%). Elemental analysis calculated for $C_{75}H_{55}EuO_8$: C, 72.87; H, 4.48. Found, C, 73.01%, H, 4.21%. FTIR (cm^{-1}): 3557 3300, 3053, 3027, 1599, 1588, 1568, 1522, 1500, 1481, 1412, 1386, 1342, 1294, 790, 760, 711, 694.

Sm(BNPD)$_3$(H_2O)$_2$. Yield 540 mg (87%). Elemental analysis for $C_{75}H_{55}SmO_8$: C, 72.96%; H, 4.49%. Found, C, 72.53%, H, 4.03%. FTIR (cm^{-1}): 3583 3210, 3056, 3032, 1600, 1589, 1568, 1522, 1500, 1481, 1411, 1385, 1342, 1294, 790, 759, 711, 694.

The Ln(BNPD)$_3$·2H_2O complexes were covalently attached by coordination with the ancillary ligand included in the polymer chain, VBPTpy (BCP1) or PhenA (BCP2), in the following way: the block copolymer and Ln(BNPD)$_3$·2H_2O complexes were dissolved in Toluene in stoichiometric proportion (BCP1, VBPTpy:Ln, 1:1; Eu/Sm molar ratio of 0.286) or (BCP2, PhenA: Ln, 1:1; Eu/Sm molar ratio of 0.081) and heat overnight at 65 ºC. The yellow solution was cooled down to room temperature, filtered through 0.22 µm, and the solvent evaporated under vacuum. The lanthanide-doped polymer was dissolved in a suitable organic solvent to obtain a final concentration of 50 mg/mL (DMSO, BCP1) or 25 mg/mL (THF, BCP2).

Ln(BNPD)$_3$L complexes

Ln(BNPD)$_3$L complexes were synthesized as a reference for the optical and infrared characterization of the BCP1-complexes and BCP2-complexes conjugates.

MPTpy or MPhen (0.1 mmol) and the corresponding Ln(BNPD)$_3$·2H_2O complex (0.1 mmol) were mixed in Toluene (5 mL) and heated overnight at 65 ºC. The obtained yellow solution was concentrated to half its volume and then kept at −20 °C for several days. The obtained precipitate was collected by filtration, washed with a minimum amount of cold Toluene, and dried under vacuum at 50 ºC to yield a faint yellow solid.

Eu(BNPD)$_3$MPhTpy**.** Yield 85 mg (56%) of a yellow solid. Elemental analysis calculated for C97H68EuN3O6: C, 76.47; H, 4.50; Eu, 9.97; N, 2.76. Found, C, 77.31; H, 4.57; N, 1.84. FTIR (cm^{-1}): 3053, 3026, 1588, 1569, 1543, 1519, 1498, 1480, 1449, 1437, 1411, 1387, 1342, 1291, 1190, 787, 759, 694.

Sm(BNPD)$_3$MPhTpy. Yield 93 mg (61%) of a yellow solid. Elemental analysis calculated for $C_{97}H_{68}SmN_3O_6$: C, 76.55; H, 4.50; N, 2.76. Found, 75.52; H, 4.72; Eu, 9.75; N, 1.80. FTIR (cm^{-1}): 3053, 3029, 1588, 1568, 1541, 1520, 1499, 1481, 1450, 1437, 1411, 1386, 1342, 1293, 1190, 787, 759, 694.

Eu(BNPD)$_3$MPhen Yield 80 mg (57%) of a yellow solid. Elemental analysis calculated for $C_{88}H_{61}EuN_2O_6$: C, 75.80; H, 4.41; Eu, 10.90; N, 2.01. Found, 75.52; H, 4.72; N, 1.80. FTIR (cm^{-1}): 3053, 3028, 1587, 1568, 1541, 1519, 1499, 1481, 1449, 1438, 1417, 1391, 1343, 1293, 1192, 789, 759, 694.

Sm(BNPD)$_3$MPhen Yield 90 mg (65%) of a yellow solid. Elemental analysis calculated for $C_{88}H_{61}SmN_2O_6$: C, 75.89; H, 4.41; N, 2.01. Found, 75.52; H, 4.72; Sm, 9.75; N, 1.80. FTIR (cm^{-1}): 33052, 3028, 1587, 1568, 1540, 1519, 1499, 1481, 1448, 1438, 1416, 1391, 1343, 1292, 1192, 788, 759, 694.

Hydrophobic block polymer in the nanoparticles

The hydrophobic block in both dual heater-thermometer core@shell NPs and single Sm^{3+}/Eu^{3+}-bearing thermometric nanomicelles is a brush-like block of methoxy (MPEG(M)A) and hydroxyl (PEG(M)A) end-capped polyethylene glycol (meth)acrylates. The hydrophobic block is a vinyl Pyridine (4-VP) or Cholesterol acrylate (Chol A) polymer endowed with a discrete number of the auxiliary ligand VBzPhTpy or PhenA. The general formula of the lanthanide complexes is Ln(L1)$_3$L2 where L1 represents the BNPD β-diketonate ligand and L2 the ancillary ligands PhenA or VBPTpy. The complexes are covalently attached to the polymer and after the addition of water to the polymer organic solution, polymeric nanostructures are formed by self-assembly, entrapped the iron oxide cores in the hydrophobic block in the case of the single Sm^{3+}/Eu^{3+}-bearing thermometric nanomicelles.

Hydrophilic block polymer in the nanoparticles

The hydrophilic PEG block forms the outer shell of the micelles and the non-water-soluble 4-Vinyl Pyridine or acrylate Cholesterol blocks containing the lanthanide complexes form the inner shell. The presence of the PEG block provides simultaneously stability in aqueous media, biocompatibility, enhanced cellular uptake, and low toxicity to the nanostructures.

Dual heater-thermometer core@shell nanoparticles

A scheme of the synthesis procedure of the dual heater-thermometer core@shell NPs is shown in Figure S4. The coating of the NPs can be described as follows: The ferrofluid was centrifuged, the pellet containing the γ-Fe_2O_3 NPs was washed with MeOH by centrifugation, redispersed in methanol (20 mg·mL^{-1}), and mixed with the polymer containing the lanthanides DMSO solution (50 mg·mL^{-1} BCP1) in a ratio 1:1 by volume. Water was added dropwise under gentle stirring to a final content of water of 85% v/v. The dispersion was sonicated, filtered through 0.22 µm, and purified and concentrated by magnetic separation using an LS Column (MACS® Miltenyi Biotec). The dual heater-thermometer core@shell NPs were collected in MilliQ water.

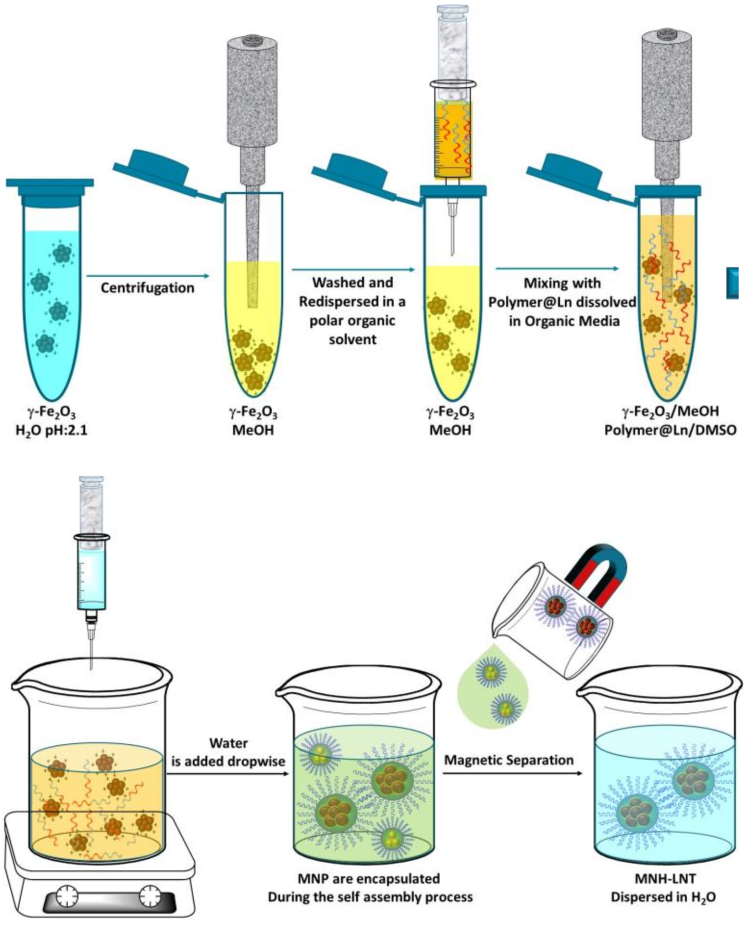

Figure S4. Scheme of the synthesis of the dual heater-thermometer core@shell NPs.

Core@shell magnetic nanoparticles

MNPs without lanthanide complexes used in hyperthermia experiments were synthesized from the γ-Fe_2O_3 NPs by coating with a P4VP-b-P(MPEGA-co-PEGA) copolymer as described elsewhere.[29] The polymer was added to a suspension of γ-Fe_2O_3 NPs at pH=2, then the pH was increased to 7.4, and the suspension was filtered through 0.22 μm membrane filters and purified by magnetic separation.

Rhodamine B labeled magnetic nanoparticles

MNPs labeled with RhB used in the confocal microscopy experiments were prepared similarly, but using a polymer containing RhB residues (P4VP-b-P(MPEGA-co-RhodPEGMA-carboxylicPEGMA) prepared as described previously.[29]

Sm^{3+}/Eu^{3+}-bearing thermometric nanomicelles

The nanomicelles were formed by self-assembly. Water was added dropwise to a THF solution of BCP2 polymer containing the lanthanides (25 $mg\cdot mL^{-1}$) under gently stirring to a final content of water of 85% v/v. The organic solvent was removed by dialysis against water using a dialysis tubing cellulose membrane with a molecular cut-off of 12000 Da. Finally, the micellar suspension was filtered through a 0.22 μm membrane filter. The polymer lanthanide-conjugate weight content (3.3 $mg\cdot mL^{-1}$) was determined by gravimetry in freeze/vacuum-dried samples. The concentration of the lanthanide in the micellar suspension was determined by ICP-OES. ([Ln]: 0.34 mM, Eu/Sm molar ratio 0.084). The Ln^{3+} ions content in the micelles was 1.53% w/w).

Methods

Characterization of polymers

Nuclear magnetic resonance (NMR)

^{1}H-NMR spectroscopy was used to confirm the synthesis of the Macro CTAs and the final BCPs and determine the number average molecular weight, Mn (NMR), monomer percentage conversion, and composition of the polymers (degree of polymerization (DP), Mole fraction (X) and Weight fraction (X_w) for each monomer.

Proton (^{1}H), and carbon (^{13}C) NMR and NMR spectra were recorded at room temperature in a BRUKER AV-400 spectrometer (400 MHz, for ^{1}H, and 100 MHz for ^{13}C) using $CDCl_3$ or DMSO-d6 as solvent. ^{1}H and ^{13}C chemical shifts (δ) are reported in ppm and are referenced to the solvent peak the coupling constants (J) are given in Hz.

Gel permeation chromatography (GPC)

Molecular weights (Mn, Mw) and polydispersity (Mw/Mn) were measured by GPC using a Water Alliance 2695 liquid chromatography system with a Waters 2424 evaporation light scattering detector and a Waters 2998 PDA detector (Waters, Milford, MA, USA), using two PLgel 5µm MIXED-C Agilent columns (7.5×300 mm) and THF (HPLC grade) as eluent (flow 1 mL min^{-1}). The calibration of the liquid chromatography system was made with narrow molecular weight standards of polymethacrylate (PMMA).

Size-exclusion chromatograms (SEC) of both the macro chain transfer agent and the final diblock copolymers showed a narrow polymer distribution indicating the formation of well-defined polymers with good polydispersity.

The GPC curve for the P(MPEGMA-st-PEGMA)-b-P(4VP-st-VBPTpy) block copolymer doesn’t shift to higher molecular weights (lower retention times) after chain extension, as could be expected. A combination of different factors can influence these results. The hydrophobic block of the BCP here presented has relatively low molecular weight with an increase of just 5400 Da (determined by NMR) with respect to the MacroCTA, thus a lack of resolution in these molecular weight range of the columns used, combined with a slight interaction of the pyridine and terpyridine units of the polymer and the different behavior of both polymers with the solvent could explain these results.

Molecular weight and molecular weight distribution and composition of the block copolymer and the MacroCTA measured are listed in Table S2 and Table S3.

Table S2. Molecular weight and composition of the block copolymer P(MPEGMA-st-PEGMA)-b-P(4VP-st-VBPTpy) (BCP1) and its MacroCTA P(MPEGMA-co-PEGMA) (MacroCTA 1) determined by ^{1}H NMR spectroscopy and GPC.

	NMR				SEC		
Polymer	Mn (Da)	DP	X	Xw	Mw (Da)	Mn (Da)	PDI
MacroCTA1	12900	MPEGMA			13363	11955	1.11
		19	0.70	0.72			
		PEGMA					
		8	0.30	0.26			
BCP1	18400	MPEGMA			12456	10.939	1.14
		19	0.29	0.50			
		PEGMA					
		8	0.12	0.18			
		VP					
		35	0.52	0.20			
		BPTpy					
		4	0.06	0.10			

Table S3. Molecular weight and composition of the block copolymer P(MPEGA-st-PEGA)-b-P(CholA-st-PhenA) (BCP2) and its MacroCTA P(MPEGA-co-PEGA) (MacroCTA 2) determined by ^{1}H NMR spectroscopy and GPC.

	NMR				SEC		
Polymer	Mn (Da)	DP	X	Xw	Mw (Da)	Mn (Da)	PDI
MacroCTA2	13350	MPEGMA			9440	8770	1.08
		19	0.70	0.69			
		PEGMA					
		8	0.30	0.29			
BCP2	19100	MPEGMA			11620	10720	1.08
		19	0.46	0.50			
		PEGMA					
		8	0.19	0.20			
		CholA					
		11	0.27	0.23			
		PhenA					
		3.4	0.08	0.05			

Fourier-transform infrared spectroscopy (FTIR)

FTIR spectra were recorded in a Perkin Elmer Spectrum 100 FT-IR spectrometer equipped with a universal attenuated total reflection sampling accessory. The spectra were collected over the 4000–380 cm^{-1} range (attenuated total reflection correction performed).

The FTIR analysis confirmed the chemical structure of the block copolymers and the successful anchoring of the complex. The FTIR spectra (Figure S5) of the $Ln(BNPD)_3(H_2O)_2$ (Ln=Sm and Eu) complexes show an intense absorption in the region 3000–3500 cm^{-1} indicating the presence of coordinated water molecules. The absence of these bands in the $Ln(BNPD)_3L$ suggests that water has been displaced successfully by the ancillary ligands. The carbonyl stretching frequency of BNPD (1537 cm^{-1}) present as a shoulder in $Ln(BNPD)_3(H_2O)_2$ complexes spectra appear as a clear peak shifted to higher wavenumbers 1547 cm^{-1} in $Ln(BNPD)_3L$ complexes and in the complexes-BCP conjugates, which also display an enhanced intensity of the peaks at 1437, 1411 and 1387 cm^{-1} compared with the tris-diketonate complexes spectra.

Infrared spectroscopy was used to characterize the aqueous suspensions of the dual heater-thermometer core@shell NPs after magnetic separation (Figure S5) and Sm^{3+}/Eu^{3+}-bearing thermometric nanomicelles (Figure S6). The FTIR spectra of the encapsulated iron oxide MNPs showed the characteristics of double peaks at 545 and 629 cm^{-1} of the maghemite, showing the existence of iron oxide particles in the encapsulated system. The peak at 1727 cm^{-1} corresponds to the stretching vibrations of the (C=O) ester carbonyl groups present in the polymer. The presence of P4VP and PEG in the coating shell can be also observed clearly by the stretching peak of pyridine rings at 1597 cm^{-1} and the C–O-C stretching vibration peak at 1104 cm^{-1}. The presence of lanthanide complexes was revealed by the presence of characteristic vibrational 1650-1450 cm^{-1} bands associated with the metal-ligand coordination bonds. A comparison of the spectrum of polymer powders obtained by evaporation of the stock solutions in toluene with that of the dual heater-thermometer core@shell NPs pellets obtained by freeze-drying of the ferrofluid suspension showed no changes in the polymer-complex conjugate bands indicating that the integrity of the complex was preserved during the coating and the magnetic separation processes.

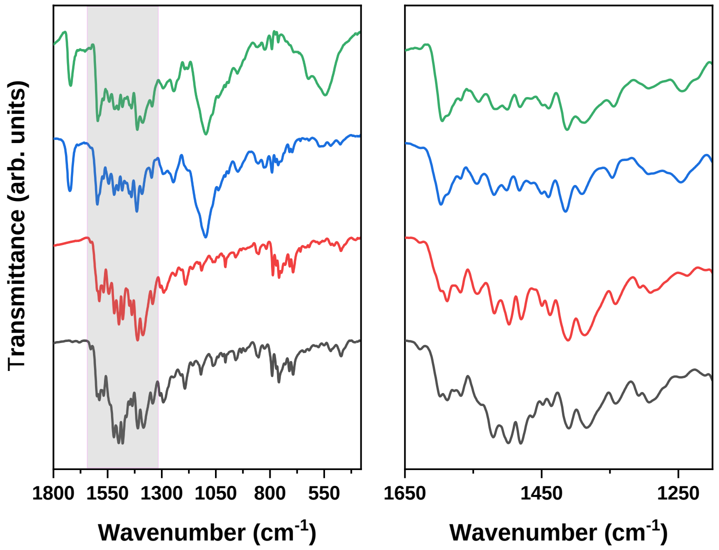

Figure S5. FTIR spectra $Eu(BNPD)_3(H_2O)_2$ (black line), $Eu(BNPD)_3$MPTpy (red line), BCP1-complexes conjugated P(MPEGMA-st-PEGMA)-b-P(4VP-st-VBPTpy@$Eu_{0.225}$ $Sm_{0.775}(BDPD)_3$) (blue line) and dual heater-thermometer core@shell NPs after magnetic separation and freeze-drying (green line).

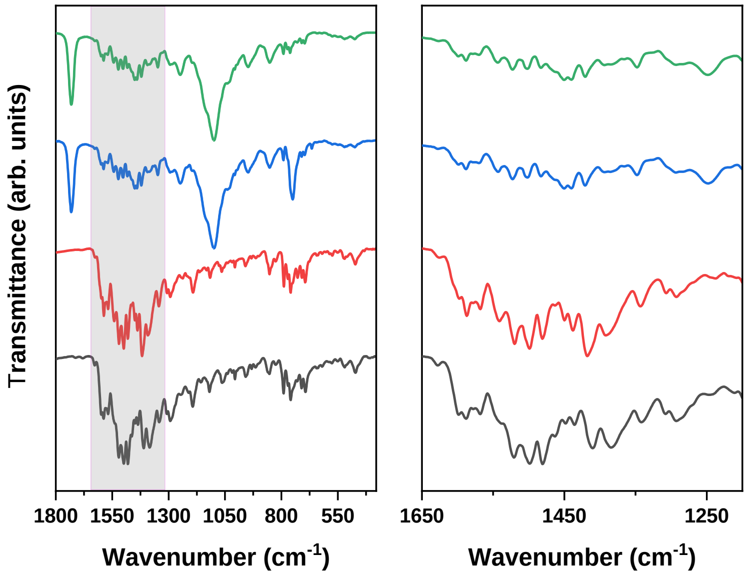

Figure S6. FTIR spectra of $Eu(BNPD)_3(H_2O)_2$ (black line), $Eu(BNPD)_3$MPhen (red line), BCP2-complexes conjugated P(MPEGA-st-PEGA)-b-P(CholA-st-PhenA@$Eu_{0.075}Sm_{0.925}$ $(BDPD)_3$) after toluene evaporation (blue line) and Sm^{3+}/Eu^{3+}-bearing thermometric nanomicelles after freeze-drying (green line).

Thermal Analysis

Thermogravimetry (TGA) and Differential Thermal (DTA) measurements were performed using a simultaneous DTA-TGA unit SDT2960 (TA Instruments) in the range of 25 to 700 ºC at a heating rate of 10 ºC·min^{-1} under air atmosphere, the residual weight was the weight of the iron oxide core, and the weight loss was thus attributed to the weight of the organic components coated on the surface of the NPs.

Elemental Analysis

Elemental analysis for C and H was performed using a Perkin Elmer CHN 2400 elemental analyzer with standard combustion conditions and handling of the samples in the air.

Chemical and physical characterization of the materials

Inductively coupled plasma optical emission spectrometry

Analysis of the single Sm^{3+}/Eu^{3+}-bearing thermometric nanomicelles and the dual heater-thermometer core@shell NPs was performed by inductively coupled plasma optical emission spectrometry in a plasma 40 ICP Perkin-Elmer spectrometer. The measured Fe_2O_3, Eu, and Sm concentration in the aqueous dispersion and the corresponding Eu/Sm molar ratio are gathered in Table S4. The samples for analysis were prepared by digestion of 1 mL of the corresponding dispersion with concentrated HNO_3 (1 mL) overnight and then diluting to the final volume of 10 mL with ultrapure water.

Table S4. Concentration of Fe_2O_3, Eu, and Sm determined by ICP.

**Sample**	**Fe_2O_3 (mg/mL)**	**Eu (mM)**	**Sm (mM)**	**Eu/Sm Molar ratio**
dual heater-thermometer core@shell NPs	1.65	0.032	0.112	0.286
MNPs	3.67	-	-	-
Sm^{3+}/Eu^{3+}-bearing thermometric nanomicelles	-	0.026	0.313	0.084

Transmission electron microscopy (TEM)

Transmission electron microscopy observations of uncoated MNPs were carried out in Tecnai T20 (FEI). Figure S7 shows the corresponding images, electron diffraction pattern, and particle size distribution. The dual heater-thermometer core@shell NPs (Figure S8) and the single Sm^{3+}/Eu^{3+}-bearing thermometric nanomicelles (Figure S9) were observed in an Analytical Titan Low-base (FEI). The EDS spectrum is presented in Figure S9 showing the $SmM_{\alpha 1}$, $SmL_{\alpha 1}$, and $SmL_{\beta 1,2}$ peaks.

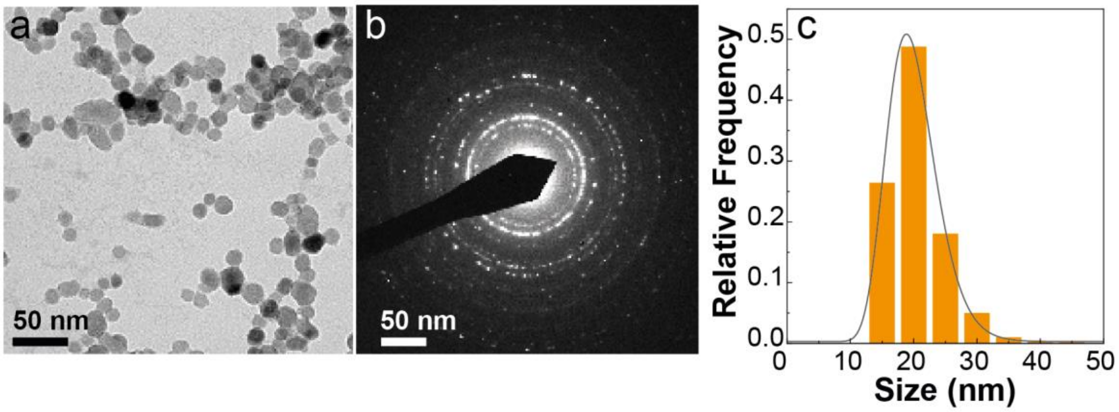

Figure S7. (a) TEM image, **(b)** electron diffraction pattern, and **(c)** size distribution of the uncoated MNPs (extracted from the TEM images). In **(c)** the line represents the log-normal function centered at mean ± SD (standard deviation), 20±5 nm, used to fit the experimental data (r^2>0.998).

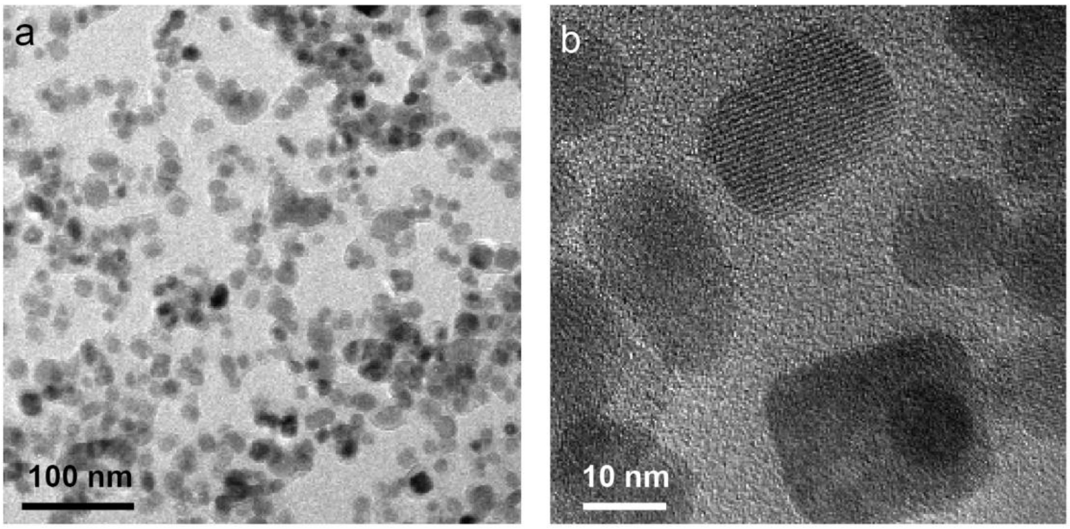

Figure S8. TEM images of **(a)** a general view and **(b)** a magnification of the dual heater-thermometer core@shell NPs.

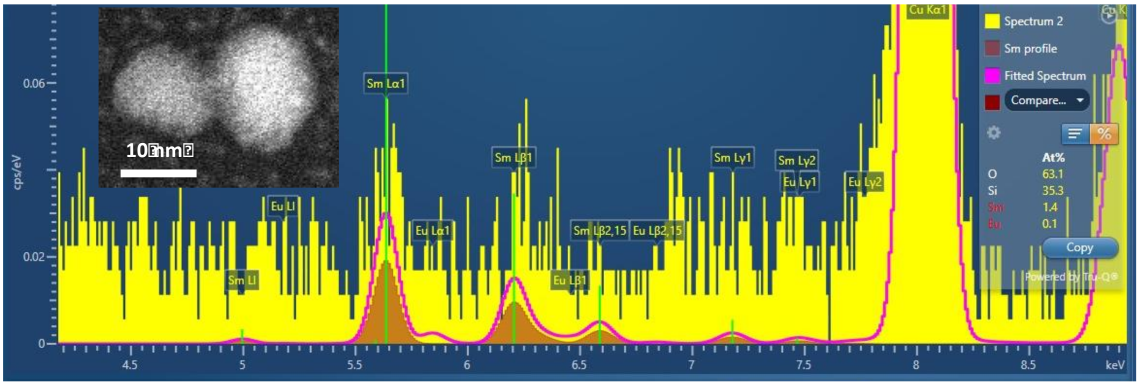

Figure S9. TEM image and EDS spectrum of Sm^{3+}/Eu^{3+}-bearing thermometric nanomicelles.

Dynamic light scattering (DLS) and zeta potential

DLS and zeta potential measurements of the ferrofluids were performed on a Malvern Zetasizer NS (Malvern Instruments Ltd., Worcestershire, UK) using a He–Ne laser with a 633 nm wavelength and a detector angle of 173º.

The samples were diluted with ultrapure water. In the case of zeta potential measurements, the samples were set in a folded capillary cell, DTS 1070 (Malvern Instruments Ltd.). The measurements were repeated three times, and the average value is reported. Size distributions of uncoated MNPs, dual heater-thermometer core@shell NPs, and single Sm^{3+}/Eu^{3+}-bearing thermometric nanomicelles are shown in Figure S7 and Figure S10. The mean hydrodynamic diameter (D_H), the polydispersity (PDI), and the zeta-potential (ζ) are listed in Table S5.

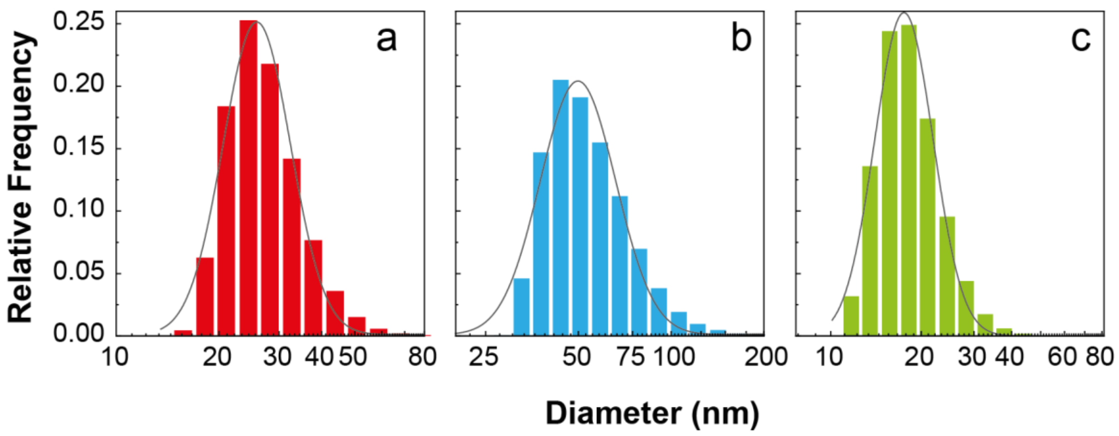

Figure S10. Distribution of the hydrodynamic diameter of **(a)** uncoated MNPs, **(b)** dual heater-thermometer core@shell NPs and **(c)** Sm^{3+}/Eu^{3+}-bearing thermometric nanomicelles. The lines correspond to the best fits of the experimental data (r^2>0.96) using log-normal distributions. The corresponding mean value (D_H), SD, and PDI are presented in Table S5.

Table S5. D_H, SD, PDI, and ζ of the ferrofluids obtained from dynamic light scattering (DLS) measurements.

**Sample**	**D_H (nm)**	**SD (nm)**	**PDI**	**ζ (mV)**
Uncoated MNPs	27.8	7.6	0.18	36
MNPs	46.8	13.9	0.16	13
Dual heater-thermometer core@shell NPs	49.8	13.1	0.07	25
Sm^{3+}/Eu^{3+}-bearing thermometric nanomicelles	18.8	4.9	0.10	1

Photoluminescence of the Ln(BNPD)$_3$L complexes and Sm^{3+}/Eu^{3+}-doped block copolymers

The excitation and emission spectra of the Ln(BNPD)$_3$L (Ln=Eu, Sm) complexes and Sm^{3+}-Eu^{3+}-doped block copolymer (P(MPEGMA-st-PEGMA)-b-P(4VP-st-VBPTpy@Eu$_{0.22}$Sm$_{0.775}$(BDPD)$_3$ and P(MPEGA-st-PEGA)-b-P(CholA-st-PhenA@Eu$_{0.075}$Sm$_{0.925}$(BDPD)$_3$) are displayed in Figure S11 and Figure S12. The spectra were obtained at room temperature by exciting the samples with a 1000 W ORIEL 66187 tungsten halogen lamp coupled to a double 0.22 m SPEX 168OB monochromator. The emission was detected using a 0.5 JAREL-ASH monochromator with a Hamamatsu R928 photomultiplier tube. The spectroscopy measurements were corrected from the system response.

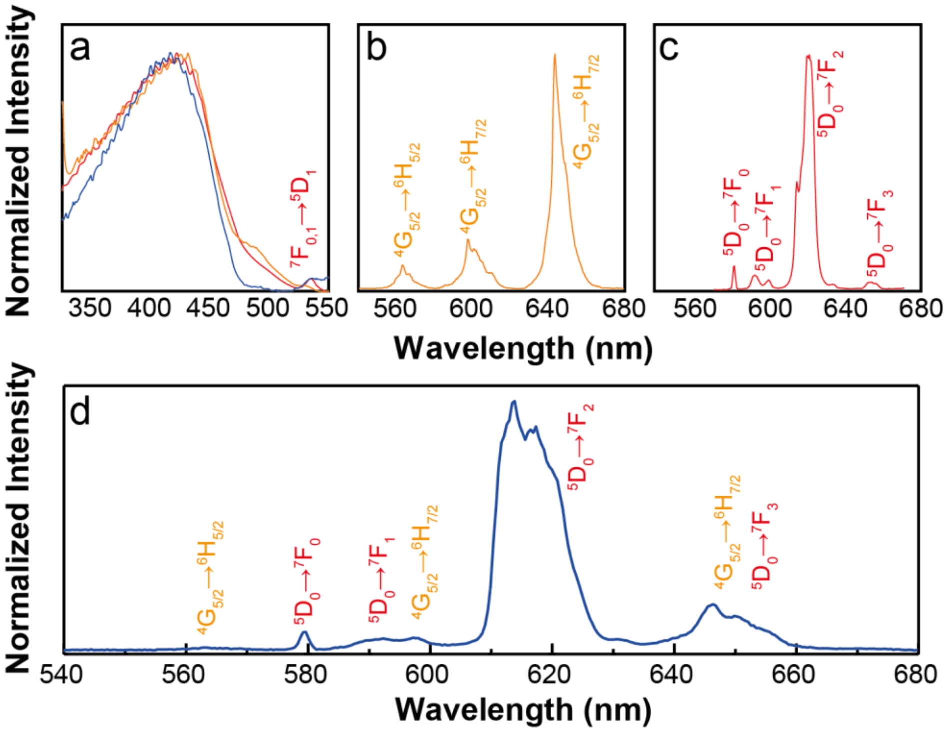

Figure S11. Room temperature **(a)** excitation spectra of Eu(BNPD)$_3$MPTpy (red line), Sm(BNPD)$_3$MPTpy (orange line) and P(MPEGMA-st-PEGMA)-b-P(4VP-st-VBPTpy@Eu$_{0.22}$Sm$_{0.775}$(BDPD)$_3$ (blue line) monitoring the $^5D_0\rightarrow{}^7F_2$ (Eu^{3+}) or $^4G_{5/2}\rightarrow{}^6H_{9/2}$ (Sm^{3+}) transitions. Emission spectra upon 425 nm excitation for **(b)** Sm(BNPD)$_3$MPTpy, **(c)** Eu(BNPD)$_3$MPTpy, and **(d)** P(MPEGMA-st-PEGMA)-b-P(4VP-st-VBPTpy@Eu$_{0.22}$Sm$_{0.775}$(BDPD)$_3$.

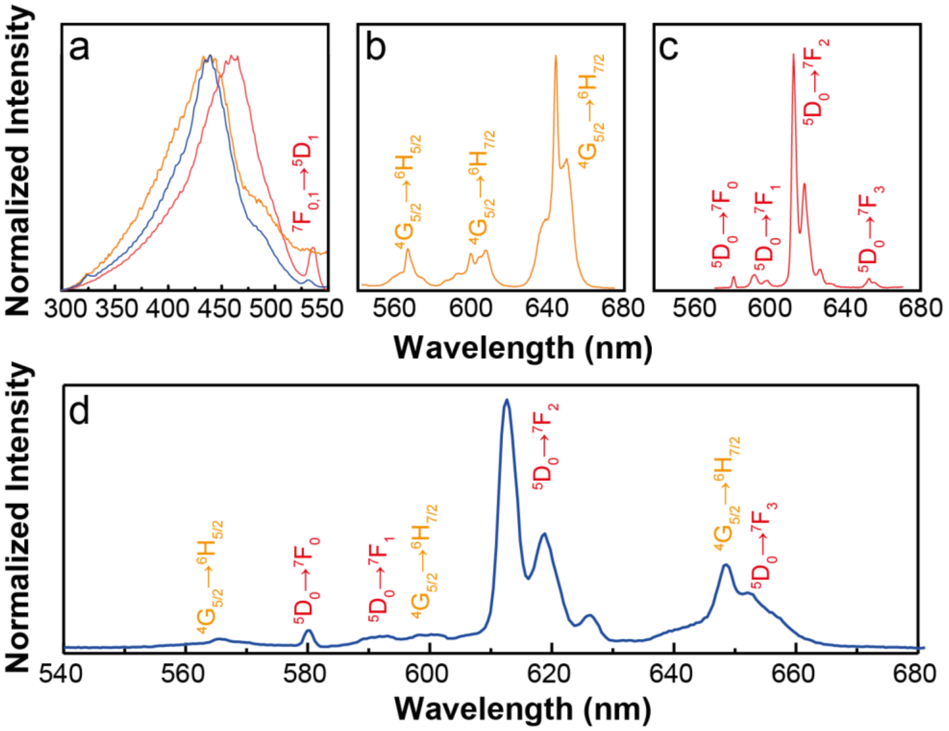

Figure S12. Room temperature **(a)** excitation spectra of $Eu(BNPD)_3MPhen$ (red line), $Sm(BNPD)_3MPhen$ (orange line), and P(MPEGA-st-PEGA)-b-P(CholA-st-PhenA@$Eu_{0.075}Sm_{0.925}(BDPD)_3$ (blue line) monitoring the $^5D_0\rightarrow{}^7F_2$ (Eu^{3+}) or $^4G_{5/2}\rightarrow{}^6H_{9/2}$ (Sm^{3+}) transitions. Emission spectra upon 425 nm excitation for **(b)** $Sm(BNPD)_3MPhen$ **(c)** $Eu(BNPD)_3MPhen$, and **(d)** P(MPEGA-st-PEGA)-b-P(CholA-st-PhenA@$Eu_{0.075}Sm_{0.925}(BDPD)_3$.

Effect of an ac magnetic field on the emission spectra of Sm^{3+}/Eu^{3+}-bearing thermometric nanomicelles

The emission spectrum of the Sm^{3+}/Eu^{3+}-bearing thermometric nanomicelles in the absence and presence of an ac magnetic field were displayed in Figure S13 showing that the calculated thermometric parameter is independent of the presence of the magnetic field.

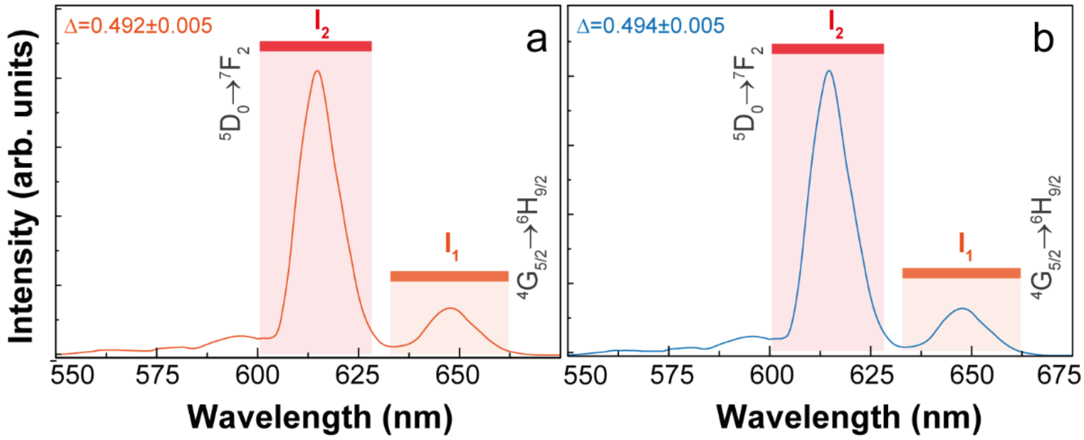

Figure S13. Emission spectrum of the Sm^{3+}/Eu^{3+}-bearing thermometric nanomicelles in the **(a)** absence and **(b)** presence of an ac magnetic field with *f*=107 kHz and *H*=42 mT. The corresponding Δ values are presented.

III. Setup for magnetic-induced-heating and optical temperature imaging

A scheme of the instrument, consisting of 2 parts, is depicted in Figure S14.

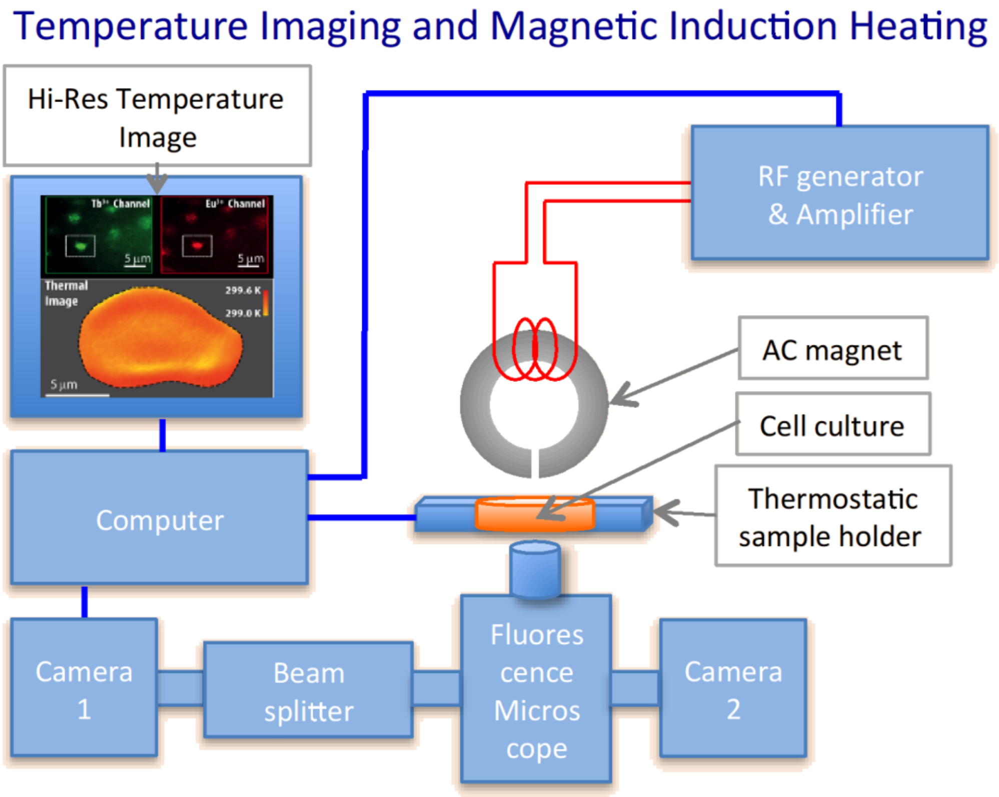

Figure S14. Scheme of the instrumental setup used in the experiments of temperature imaging of NPs internalized in cells under exposure to an AC magnetic field.

Part 1: is a temperature imaging system comprising: a) a conventional inverted fluorescence microscope (Leica DMI3000B equipped with a DFC 7000 color camera); b) a Gemini beam splitter (*Hamamatsu*) with a dichroic (*Semrock FF635-Di01)* that divides the emission beam into two beams that are passed, respectively, through an FBH650-40 bandpass filter (Semrock) for the $^4G_{5/2}\rightarrow^6H_{9/2}$ Sm^{3+} transition and an ET610/20 bandpass filter (Chroma) for the $^5D_0\rightarrow^7F_2$ Eu^{3+} transition; c) a CMOS *Orca 4.0* camera (*Hamamatsu*) that capture these two emissions, and send them to d) a computer endorsed with a MatLab software made on purpose that transforms the emission intensity images into a temperature image of the NPs internalized in the cells. This system was previously used to screen the temperature distribution in life cells[29] and a more detailed description of the setup can be found in this reference.

Part 2: of the instrument consists of an AC Magnetic Field Applicator adapted to cell cultures under microscope observation. It consists of a signal generator connected to a linear amplifier (HSA 4014) feeding current to a toroid ferrite magnet, similar to that described elsewhere[30] with a gap of 0.4 mm and a flat area underneath that contacts the cell culture well bottom. The magnet, the cell culture well, and an optical fiber thermometer are snapped within a plastic holder that is placed in a thermostatic plaque coupled to the microscope stage (Figure S15).

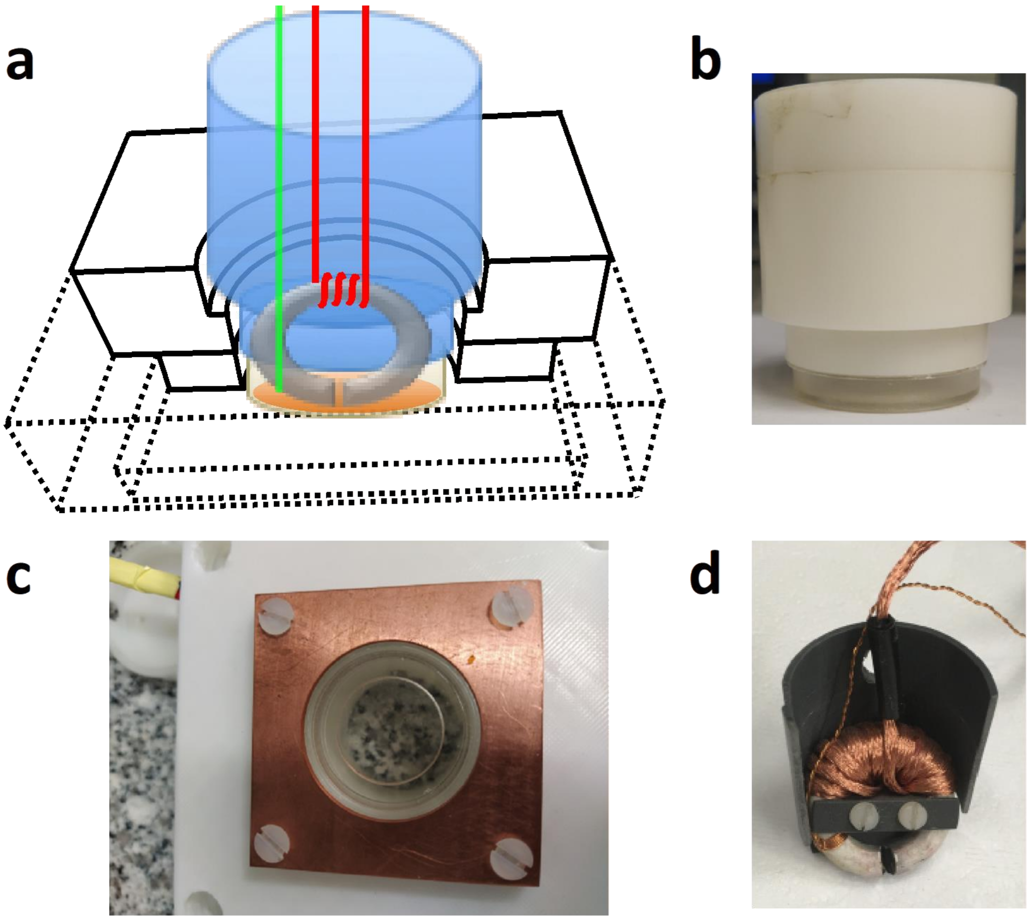

Figure S15. (a) Scheme of the thermostatic sample holder used in the temperature imaging experiments consisting of **(b)** a PVC plaque with a copper bottom heated with **(c)** a set of electric resistances coupled to a thermostat. A 35 mm glass-bottom dish containing the cell culture is plugged into a Teflon cylindrical chamber that holds **(d)** the mini electromagnet consisting of a toroid ferrite supplying the AC magnetic field.

IV. Calibration of the molecular temperature probes

The calibration of the dual heather-thermometer core@shel NPs and of Sm^{3+}/Eu^{3+}-bearing thermometric nanomicelles in water suspension was carried out in a setup depicted in Figure S16, a holder for the suspension (a cuvette placed on Peltier thermostatic holder, H), an excitation LED light source (LLS-365, Ocean Optics, centered at 365 nm, L), a bifurcated optical fiber (modified Ocean Optics QR450-7-XSR fiber with a polyether ether ketone housing instead of the usual metallic one, B), a semiconductor temperature sensor connected (Neoptix Reflex) to a temperature controller unit (TR). A portable spectrophotometer (USB-4000FL portable spectrometer, Ocean Optics, D) is connected to a PC for recording the emission spectra.

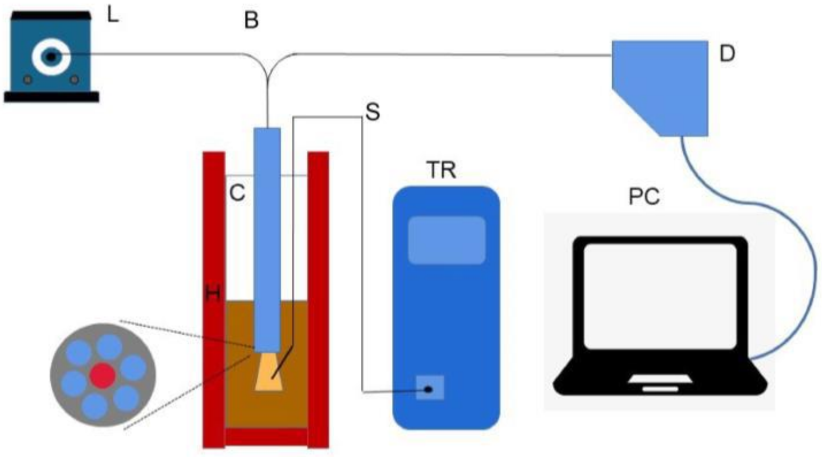

Figure S16. Setup for calibration of the dual heather-thermometer core@shel NPs and Sm^{3+}/Eu^{3+}-bearing thermometric nanomicelles in water suspensions.

The recorded emission spectra of Sm^{3+}/Eu^{3+}-bearing thermometric nanomicelles and dual heather-thermometer core@shell NPs (Figure S17a,b) were post-processed using MatLab®. A routine was implemented to import the emission spectra, remove the baseline and integrate the area under the Sm^{3+} $^4G_{5/2}\rightarrow{}^6H_{9/2}$ (I_1, integration range 600-612 nm) and the Eu^{3+} $^5D_0\rightarrow{}^7F_2$ transitions (I_2, integration range 630-650 nm). The thermometric parameter, defined as $\Delta=I_1/I_2$, is calculated by the routine, together with the corresponding uncertainty, resulting from the error propagation.

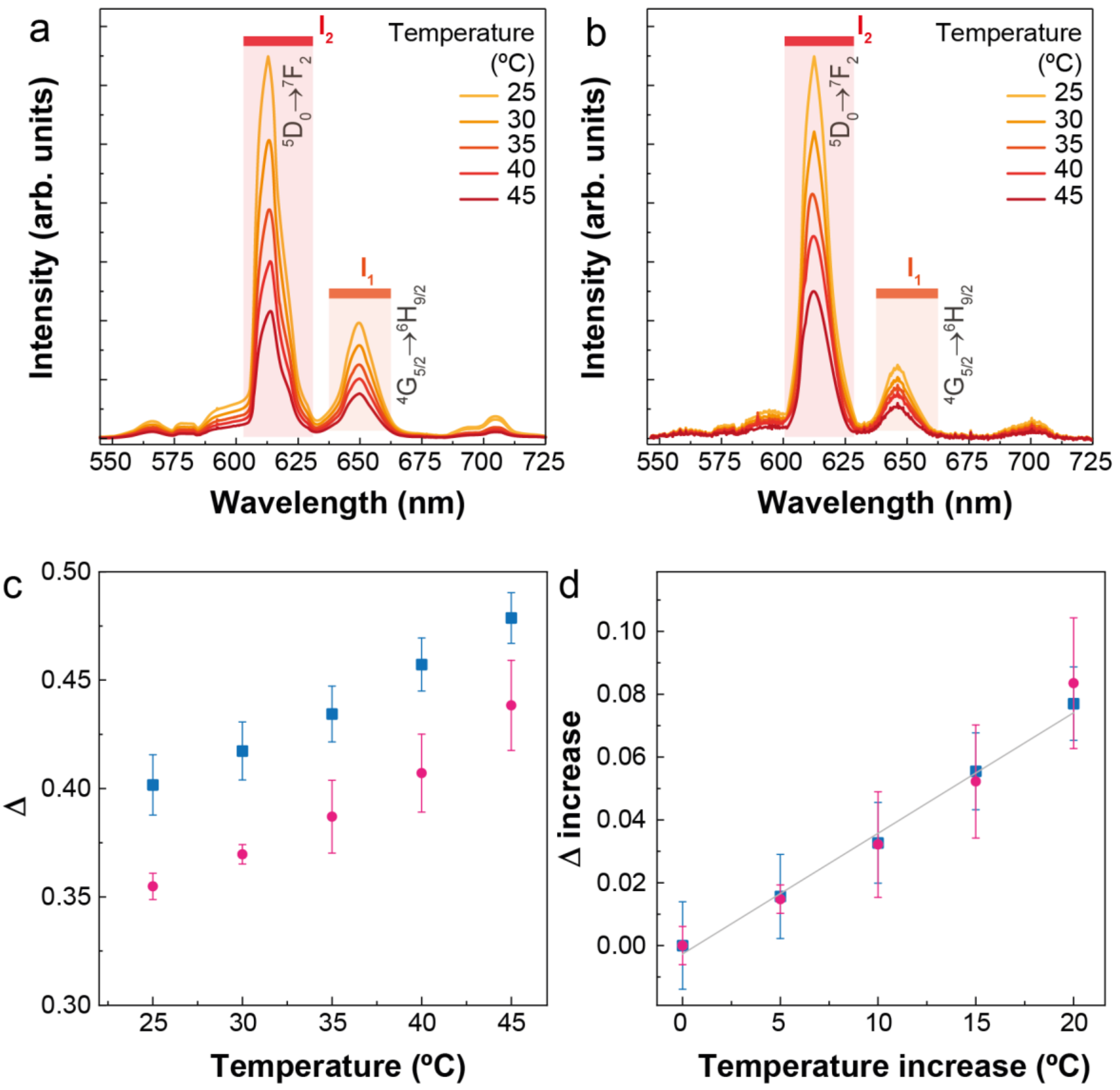

Figure S17. Emission spectra of the **(a)** Sm^{3+}/Eu^{3+}-bearing thermometric nanomicelles (blue squares) and **(b)** dual heather-thermometer core@shell NPs in water suspensions in the 25-45 ºC range. **(c)** Intensity ratio (thermometric parameter) to temperature calibration for the dual heather-thermometer core@shell NPs (magenta circles) and Sm^{3+}/Eu^{3+}-bearing thermometric nanomicelles (blue squares) in water suspensions. **(d)** Increase of the thermometric parameter with the temperature increase. Both NPs probe the increasing of the temperature with a similar change in the thermometric parameter. The line is the best linear fit of the data (r^2>0.986), slope (3.8±0.2)×10^{-3} ºC^{-1}.

The intensity-to-temperature calibration curve of the Sm^{3+}/Eu^{3+}-bearing thermometric nanomicelles in cells is obtained based on the calibration of dual heather-thermometer core@shell NPs performed in the collagen gel using the intensity images of the Eu and Sm channels acquired with the microscope camera (Figure 2b) and the information presented in Figure S17. Analyzing in this figure the dependence of the thermometric parameters in water, we observe a 0.05 shift in the Δ value of the Sm^{3+}/Eu^{3+}-bearing thermometric nanomicelles relative to that of the dual heather-thermometer core@shell NPs, despite the same slope of the two thermometers. This shift corresponds to a temperature difference of 2.9 ºC. Therefore, the intensity-to-temperature calibration curve of the Sm^{3+}/Eu^{3+}-bearing thermometric nanomicelles in cells is determined by reducing the intercept of the calibration curve of the dual heather-thermometer core@shell NPs represented in Figure 2b by a value corresponding to 2.9 ºC, *i.e.*, $\Delta = (5.9 \pm 0.1) \times 10^{-3}\mathrm{T} + (36.1 \pm 0.3) \times 10^{-2}$ (ºC).

V. Relative thermal sensitivity and temperature uncertainty

The relative thermal sensitivity (S_r) was calculated using:

$$S_r = \frac{1}{\Delta}\left|\frac{\partial \Delta}{\partial T}\right|. \qquad \textbf{(S1)}$$

and the temperature uncertainty, δT:

$$\delta T = \frac{1}{S_r}\sqrt{\left(\frac{\delta I}{I_2}\right)^2 + \left(\frac{I_1}{I_2^2}\delta I\right)^2}. \qquad \textbf{(S2)}$$

where $\delta I/I_1$ and $\delta I/I_2$ are determined by the noise fluctuations, as presented elsewhere.[31]

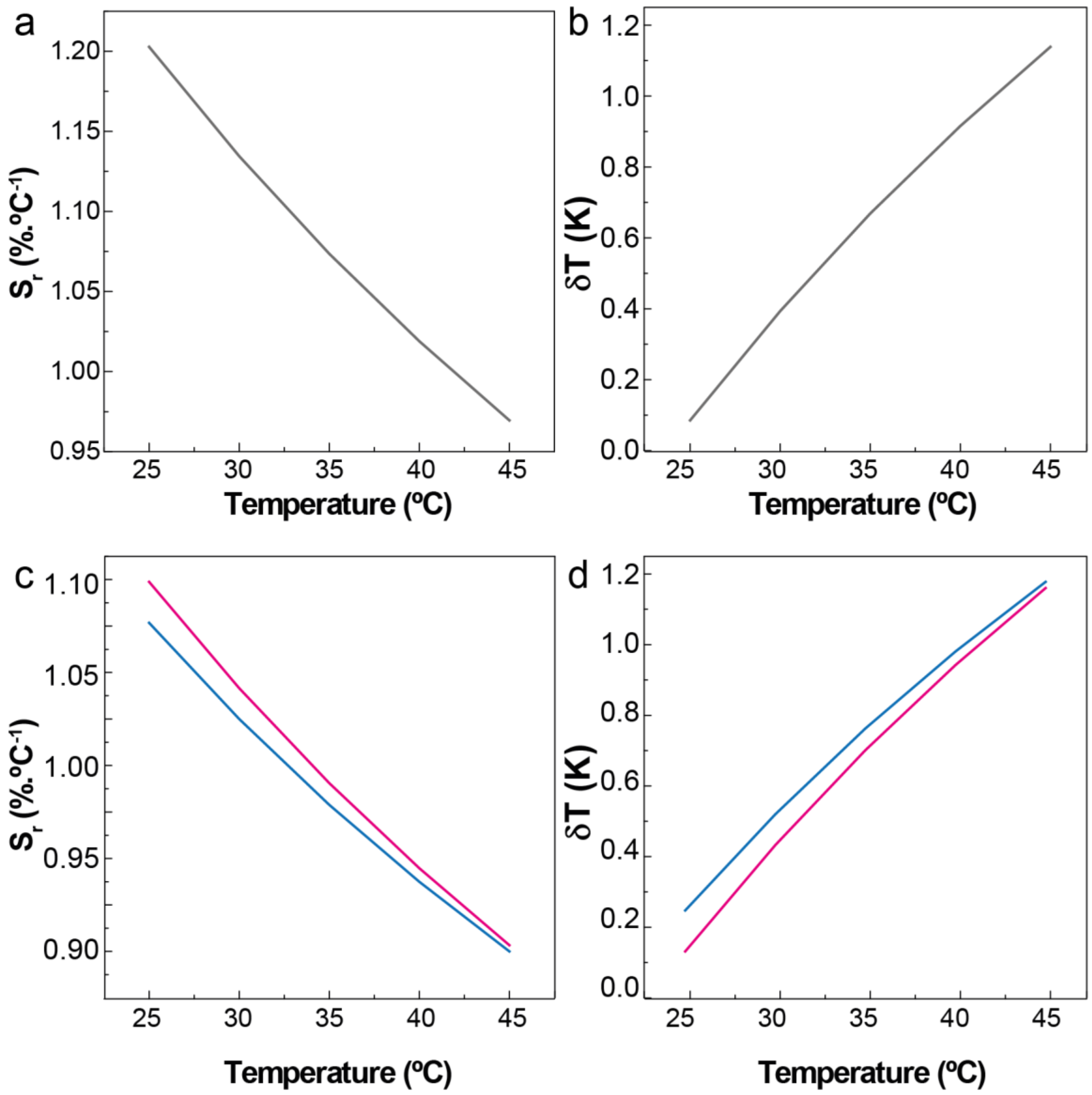

Figure S18. (a,c) Relative thermal sensitivity and **(b,d)** temperature uncertainty for Sm^{3+}/Eu^{3+}-bearing thermometric nanomicelles and dual heather-thermometer core@shell NPs. The results for the dual heather-thermometer core@shell NPs in collagen, calculated using the microscope data, are presented in **(a,b)**. In **(c,d)**, the Sm^{3+}/Eu^{3+}-bearing thermometric nanomicelles (blue) and dual heather-thermometer core@shell NPs (magenta) data were calculated from the data presented in Figure S17.

VI. Temperature imaging of living cells

About 170,000 cells were cultivated in 35 mm glass-bottom dishes (Ibidi) in 2 mL of DMEM without phosphates with 10% SFB and antibiotics for 24 h. Then, 0.2 mL of the heater-thermometer NPs suspension was added and left incubating for another 24 h at 37 ºC. Then, the culture medium was removed and cells were washed twice with DMEM medium without phosphates and SFB, and then two more times with 0.9% NaCl. Finally, we added 2 mL of 0.9% NaCl + glucose 0.9 mg·mL^{-1} were added, and the cell culture dishes were placed in a thermostatic holder as depicted in Figure S15. The temperature of the culture is measured with a fiber optic temperature sensor (Neoptix™ Reflex™). The temperature imaging experiments were performed at different culture temperatures, 23, 28, 30, and 33 ºC. The fluorescence images were taken with an exposure time of 6 s.

VII. Magnetic properties of the nanoparticles

Magnetization measurements

Magnetization hysteresis loops of the NH at several temperatures below and above the water freezing temperature were carried out in aqueous suspension on a superconducting quantum interference device (SQUID)-Based Magnetometer MPMS-XL5 from Quantum Design in a −50000 Oe to 50000 Oe field range (Figure S19). The saturation magnetization at 300 K was 74.2 emu/g (Figure S19a) The NPs suspension showed a coercivity of about 30 Oe well below the freezing point. This coercivity had already disappeared at 250 K due to NP mobility that allows the orientation of the NPs with the field by physical rotation.

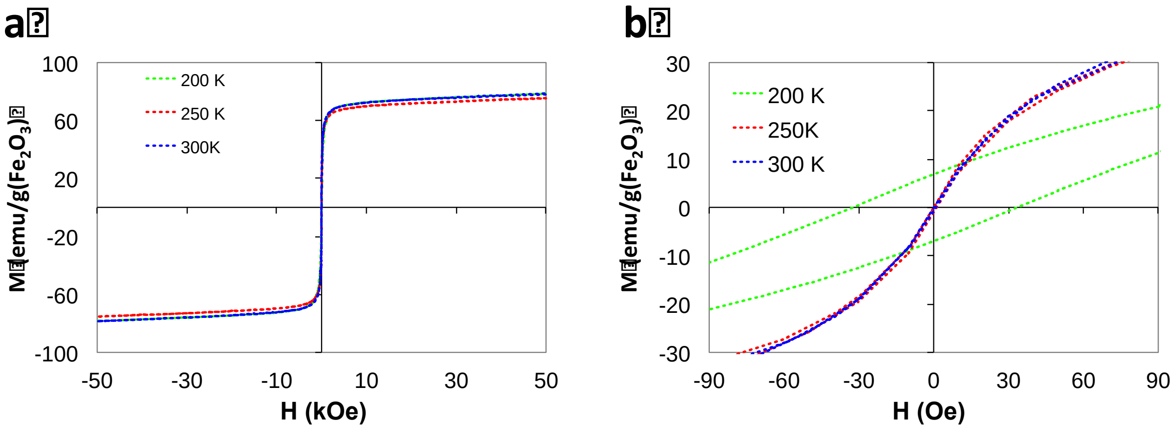

Figure S19. (a) Magnetization vs field of a water suspension of polymer-coated γ-Fe_2O_3 NPs at different temperatures and **(b)** detail of the magnetization of the sample within the range of field amplitudes used in SAR experiments.

Specific absorption rate (SAR) measurements

SAR values of MNPs suspensions were measured at different AMF amplitudes on an in-house developed equipment, as described elsewhere.[32] The SAR values were calculated from the slope at *t*=0 of the *T*(*t*) curves (**Figure S20**), by using:

$$SAR = \frac{C_{\mathrm{H_2O}}}{c_{\mathrm{Fe2O3}}} \frac{dT}{dt} \qquad \textbf{(S3)}$$

where $\boldsymbol{C_{\mathrm{H_2O}}}$ is the heat capacity of water, $\boldsymbol{c_{\mathrm{Fe2O3}}}$ is the concentration of iron oxide NPs in suspension, and *dT/dt* is the derivative of a polynomial obtained by fitting the experimental data to a second-order polynomial function (**Figure S20a**). **Figure S20b** shows the obtained SAR values at different field intensities for two frequencies. The corresponding SAR value for the magnetic field conditions (*H*=30 mT, *f*=100 kHz) used in the cell hyperthermia experiments was 45 W·g(Fe_2O_3)$^{-1}$.

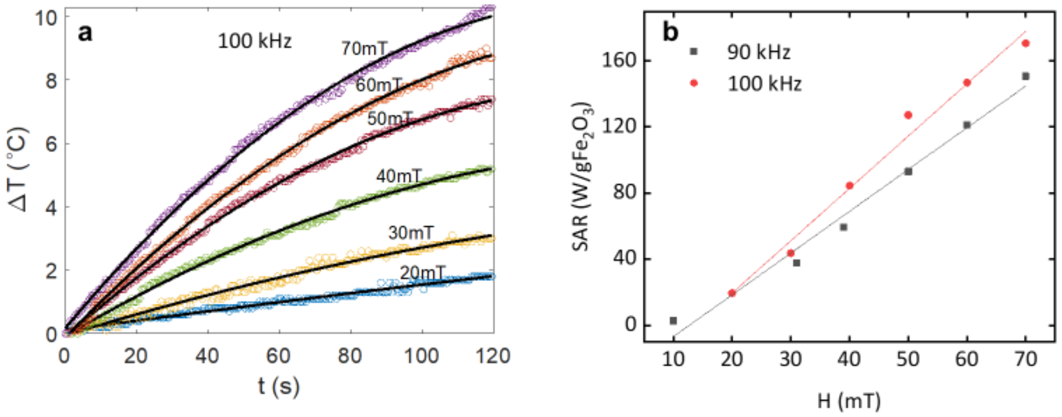

Figure S20. (a) Heating curves of iron oxide NPs in aqueous suspension (3.2 mg·mL^{-1}) under ac magnetic field, *H*, with a similar frequency (100 kHz) and different amplitudes, including those used in the cell hyperthermia experiments. The points are experimental measurements and the black lines are the fittings to a second-degree polynomial function. **(b)** Plot of SAR *vs. H* at two frequencies. The lines are guides for the eyes.

VII. Cell cultures

MDA-MB-468 breast cancer cell lines were cultured in commercial DMEM high glucose medium without phosphate, supplemented with 10% (v/v) fetal bovine serum, and 100 U/mL penicillin and 100 μg·mL^{-1} streptomycin and kept in a thermostatic incubator (Nuaire) in saturated humid air with 5% CO_2 at 37 °C (310 K). Successive subcultures and media changes were done every 2 or 3 days. Cell viability was calculated by the Trypan blue (Sigma) exclusion technique. Manipulations of cell cultures were carried out inside a vertical laminar flow cabinet (Nuaire) in sterility conditions.

Cell internalization of the nanoparticles

In total, about 470,000 cells were seeded on coverslips in 6-well plates 24 h before the treatments with NP suspensions. Then, the cells were incubated for 24 h with different concentrations of NPs (160, 320, and 480 mg Fe_2O_3·mL^{-1}). In order to eliminate the presence of non-internalized NPs in the sample, the supernatants were aspirated out and the cells were washed 3 times with cold NaCl 0.9%. Then, the cells were fixed with 4% (w/v) paraformaldehyde for 10 minutes, washed again three times with the same solution, and mounted for microscopy. As a control, MDA-MB-468 cells were cultured in the absence of NPs and prepared under the same conditions.

Cytotoxicity analysis

The cytotoxicity of the NPs was evaluated by Annexin V^+ binding assays. Briefly, cells treated with different concentrations of NPs were stained for 20 minutes at room temperature in the darkness, with Annexin-Dy634, which binds to the phosphatidylserine exposed on the surface of death cells. Staining was performed in annexin binding buffer (140 mM NaCl, 2.5 mM $CaCl_2$, 10 mM HEPES/NaOH, pH 7.4). The cell suspension was diluted with the same buffer and analyzed by flow cytometry. The results showed no apparent effect of the NPs on cell viability, even when the concentration of NPs in the culture was three times higher than that used in the hyperthermia experiments (Figure S21).

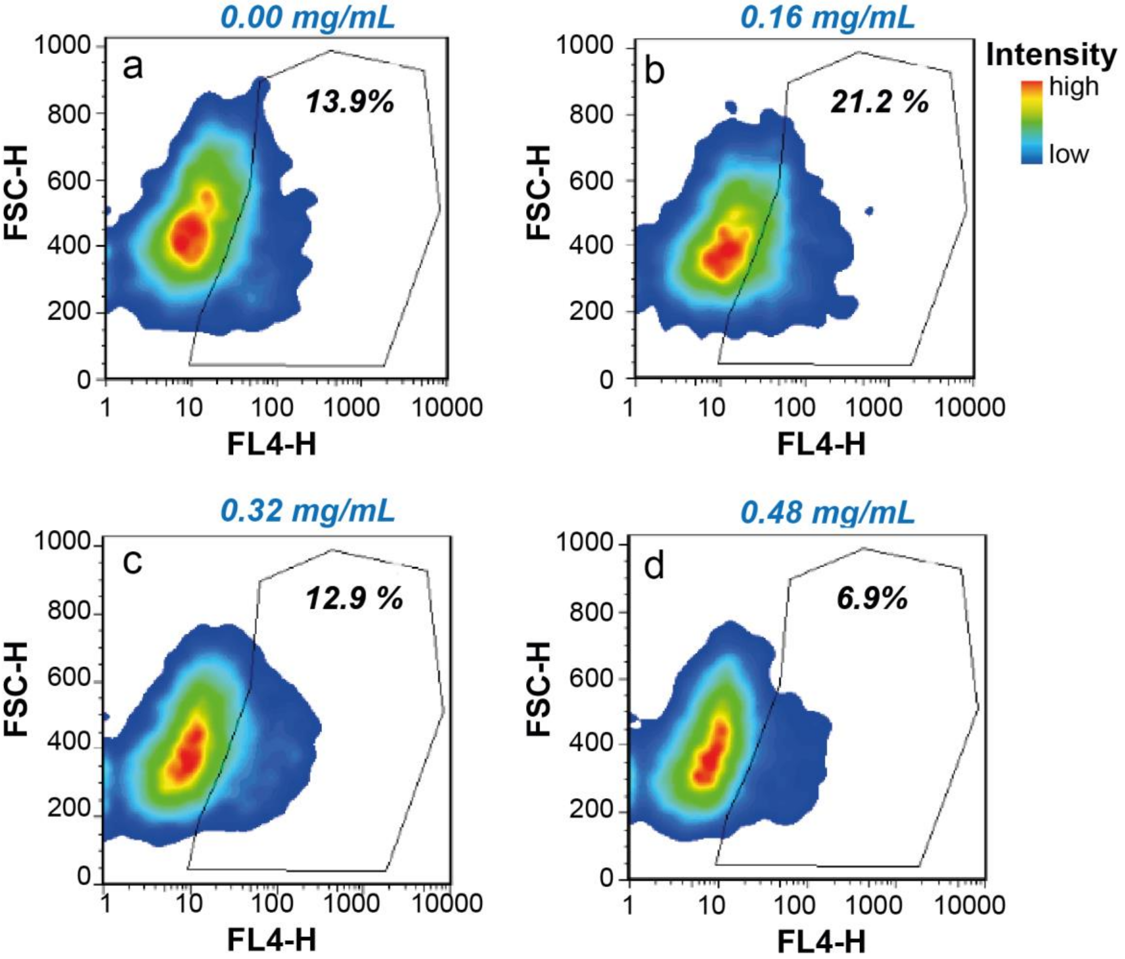

Figure S21. Cell death induction analyzed by flow cytometry. Cells were incubated with different concentrations of MNP (up to 0.48 mg(Fe_2O_3)·mL^{-1}) and cell death was measured by Annexin V-Dy634 staining. The images show FSC-H (cell size) *vs* FL4-H (Annexin V-Dy634 detection) as a density map, where the percentage of events inside the polygon gate is considered as Annexin V^+, and, thus, cell death.

TEM images of cell cultures

Ultramicrotoms of cells embedded in epoxy resin were observed by TEM. Images obtained in a 100kV microscope (JEOL JEM 1010) after incubation with dual heater-thermometer core@shell NPs are shown in Figure 3. Similar observations were carried out in cells after internalization with Sm^{3+}/Eu^{3+}-bearing thermometric nanomicelles (Figure S22).

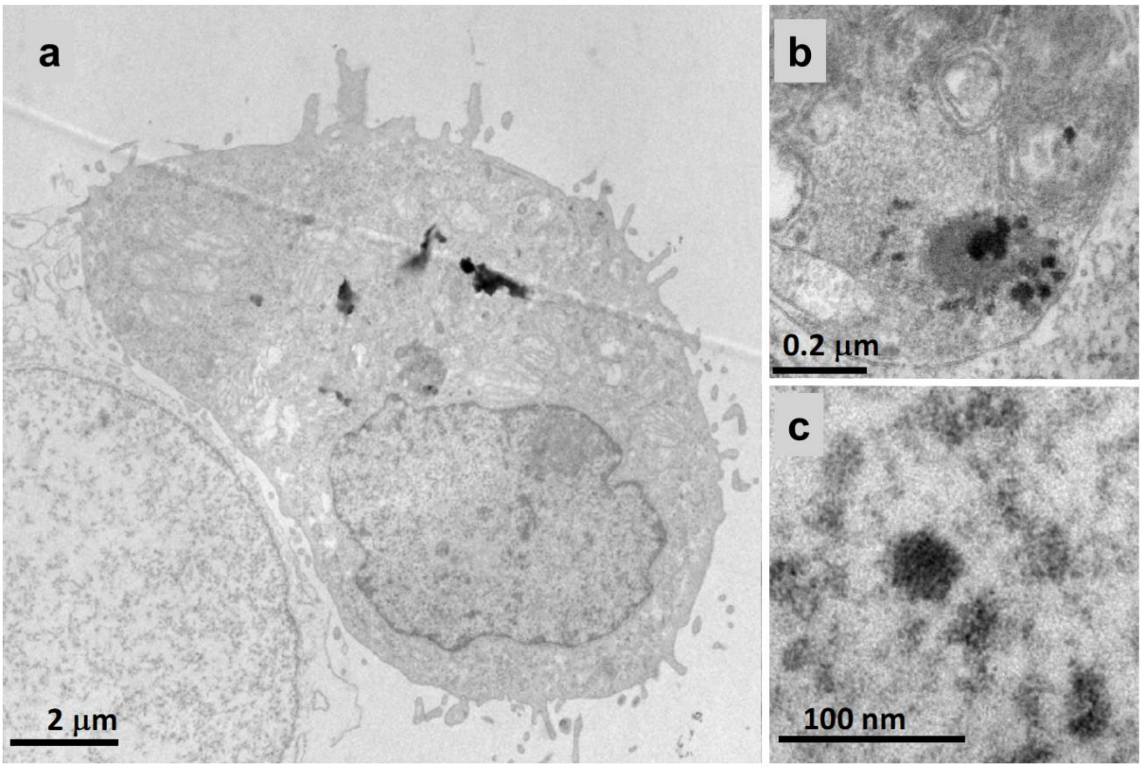

Figure S22. TEM images of an MDA-MB-468 cell after incubation with the Sm^{3+}/Eu^{3+}-bearing thermometric nanomicelles obtained with a JEOL (JEM 1010 100kV) transmission electron microscope: **(a)** image of a whole cell, **(b)** and **(c)** details of cytoplasm areas containing objects with a similar size as that of thermometric micelles. Osmium tetroxide, uranyl, and lead acetate were used for staining.

Ultramicrotoms of cell cultures were also observed with an Analytical Titan electron microscope (FEI) equipped with EDS Oxford Instruments Ultim Max TLE 100 which permitted the identification of the NPs from the Fe signal and confirmed the presence of the dual heater-thermometer core@shell NPs inside the nucleolus (Figure S23). It was observed that both OsO_4 and lead staining agents were strongly absorbed on the nanoparticle surface hampering the detection by the sight of the iron oxide cores.

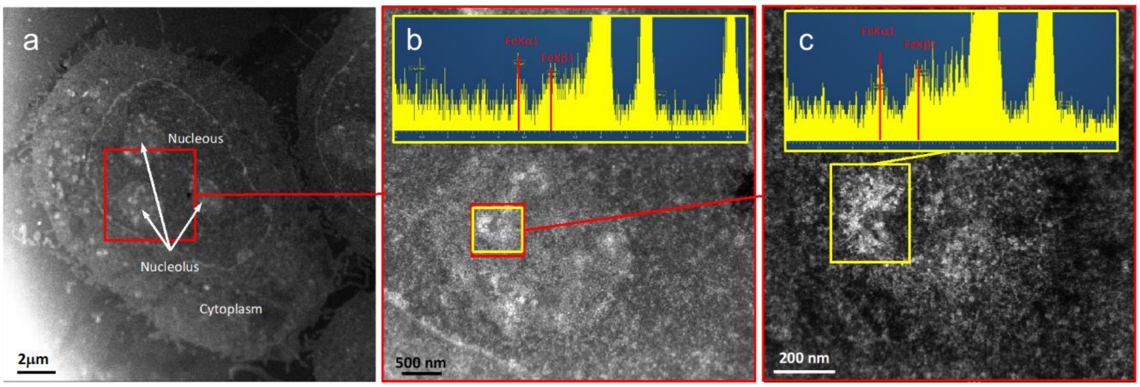

Figure S23. STEM (Titan FEI) observations of ultramicrotomes of MDA-MB-468 cells after incubation with MNPs. **(a)** Image of a whole cell. **(b), (c)** Selected areas of the nucleus and nucleolus and the corresponding EDS spectra showing the presence of Fe.

Ultramicrotoms of cell cultures incubated with Sm^{3+}/Eu^{3+}-bearing thermometric nanomicelles and stained with OsO4, PbAc, and UO_2 were also examined by STEM. No Fe traces were detected in the EDS spectrum of selected areas in the nucleus, mitochondria or cytoplasm of cells (Figure S24). Meanwhile, line profiles across bright regions in the nucleus, the mitochondria, and the cytoplasm and spectra of selected areas in non-treated cells did not reveal any presence of iron as it can be appreciated in Figure S25. However, the presence of iron in cell samples incubated with dual heater-thermometer core@shell NPs was quite evident both in EDS spectra and line profiles (Figure S26).

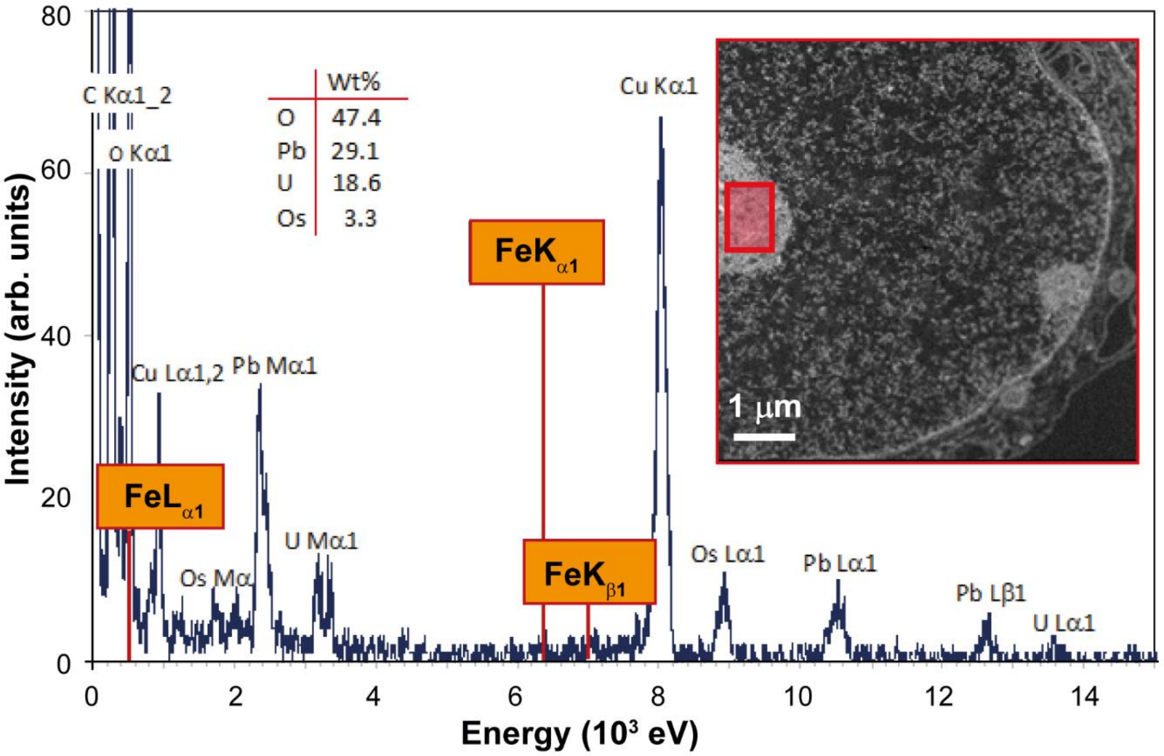

Figure S24. EDS spectrum of a selected area in the nucleolus of cells incubated with Sm^{3+}/Eu^{3+}-bearing thermometric nanomicelles and stained with lead acetate, uranyl acetate, and osmium tetroxide.

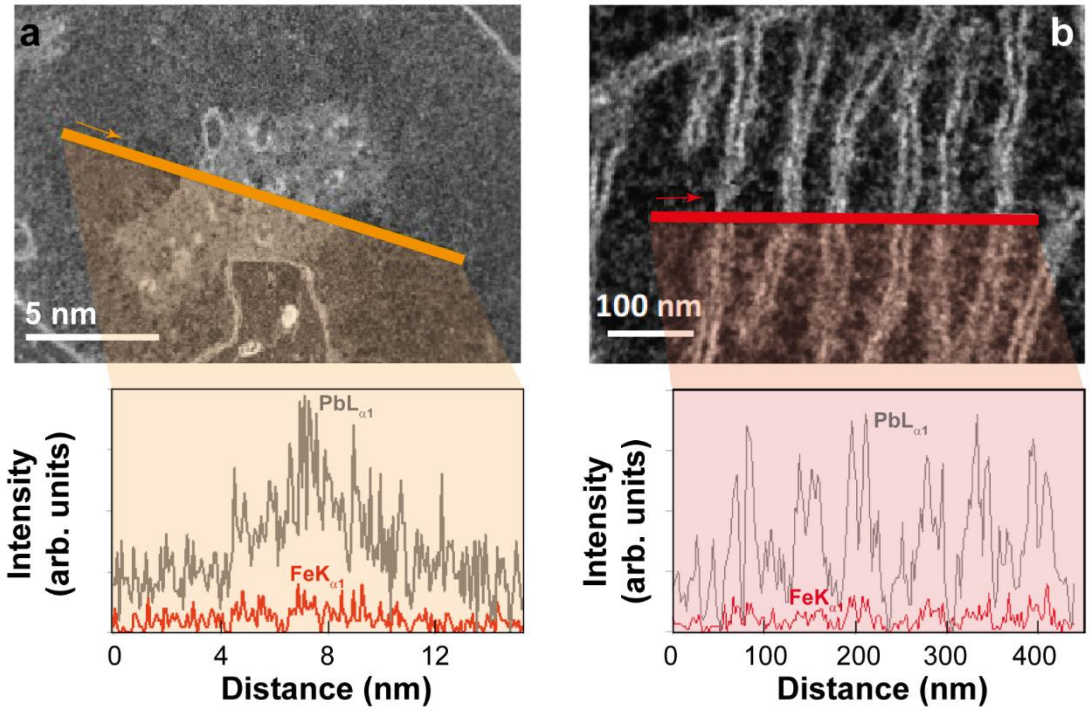

Figure S25. Line profiles across **(a)** the nucleus and **(b)** the mitochondria of non-treated cells stained with lead acetate, uranyl acetate, and permanganate.

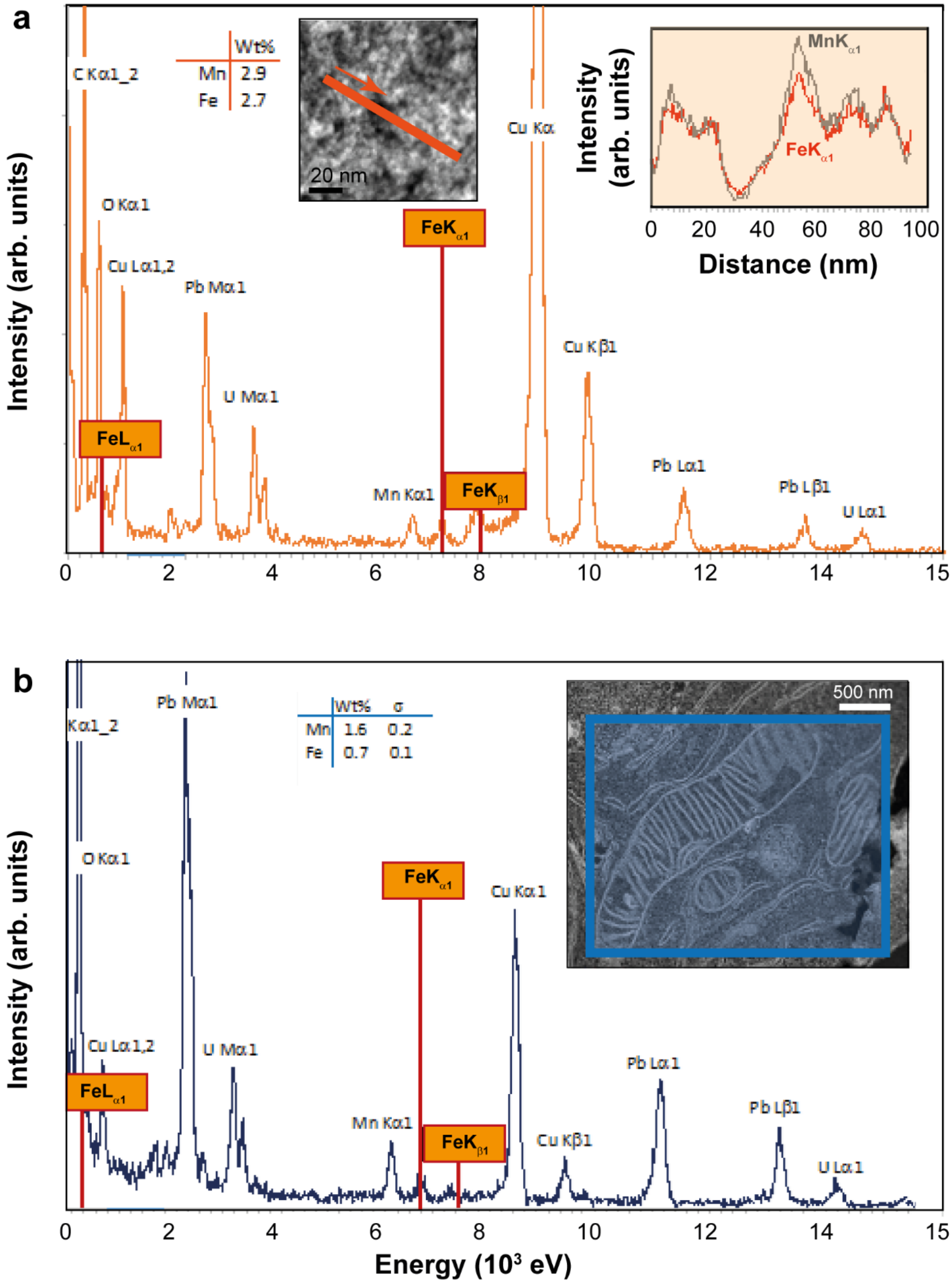

Figure S26. EDS spectrum and composition profile **(a)** across a line in the cytoplasm and **(b)** in a selected area, including several mitochondria, of cells incubated with dual heater-thermometer NPs and stained with lead acetate, uranyl acetate, and permanganate.

Optical microscope images of cell cultures

All the cells were observed by optical microscopy before and after incubation with the distinct NPs. The morphology of the cells did not change after the incubation as observed in phase-contrast images taken by the color camera (Figure S27) and the CMOS camera (Figure S28). Fluorescence images of control cells did not show any emission in either Eu^{3+} or Sm^{3+} channels (Figure S29), whereas those of cells incubated with the dual heater-thermometer core@shell NPs showed clear luminescence emission in both channels, indicating that the emission collected comes exclusively from the lanthanide ions in the dual heater-thermometer core@shell NPs.

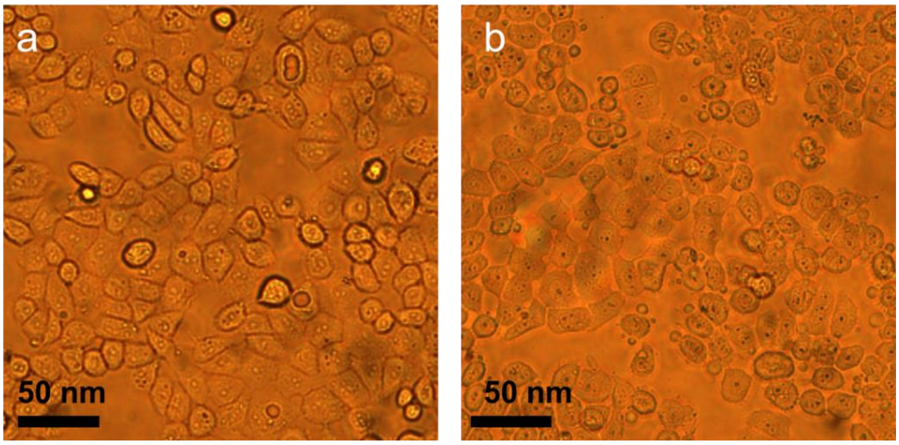

Figure S27. Phase contrast images of MDA-MB-468 cell cultures before **(a)** and after **(b)** the incubation with dual heater-thermometer core@shell NPs taken with a Leica color camera.

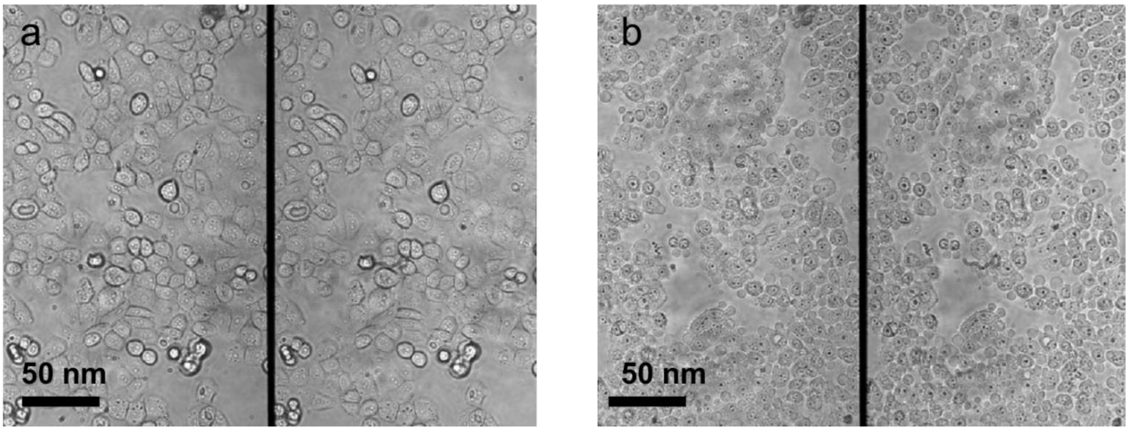

Figure S28. Phase contrast images of MDA-MB-468 cell cultures before **(a)** and after **(b)** the incubation with dual heater-thermometer core@shell NPs taken with a CMOS camera.

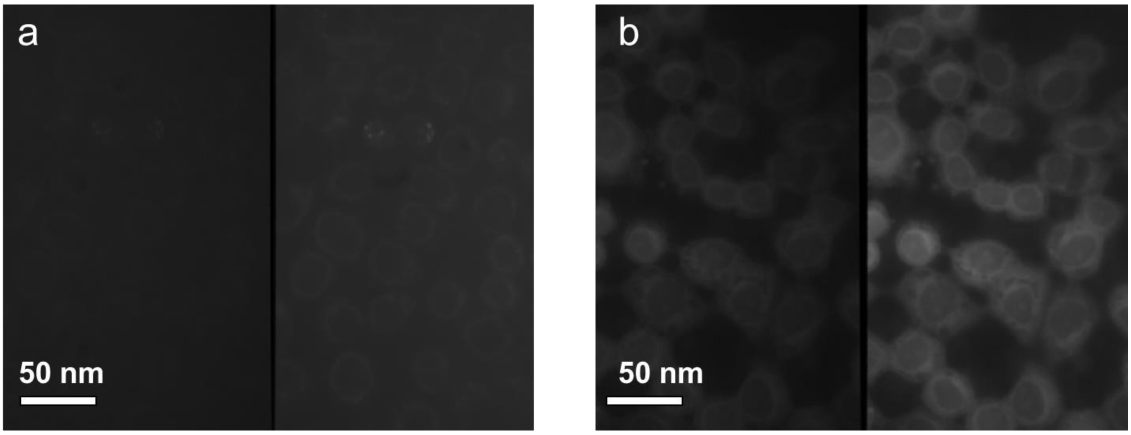

Figure S29. Sm^{3+} or Eu^{3+} channels fluorescence images of MDA-MB-468 cell cultures before **(a)** and after **(b)** the incubation with dual heater-thermometer core@shell nanostructures taken with a CMOS camera. Cells without NPs did not show any emission in either Sm^{3+} or Eu^{3+} channels.

Confocal microscope images

To visualize the location of the MNPs in the cells, cells were incubated with RhB-labeled MNPs and they were examined by confocal microscopy (Zeiss LSM 880). Figure S30 shows the emission of the RhB dye superimposed on a dark field image of the cells. Figure S31 shows XY in-plane merged fluorescence images at different Z depths of the NPs in cells that have been stained with DRAQ5^{TM} to mark the nucleus following the manufacturer. The NPs are distributed uniformly in the cytoplasm, and some of them appear in the interior of the nucleus. Figure S32 shows comparative images of XY, XZ, and YZ plane images of the cells.

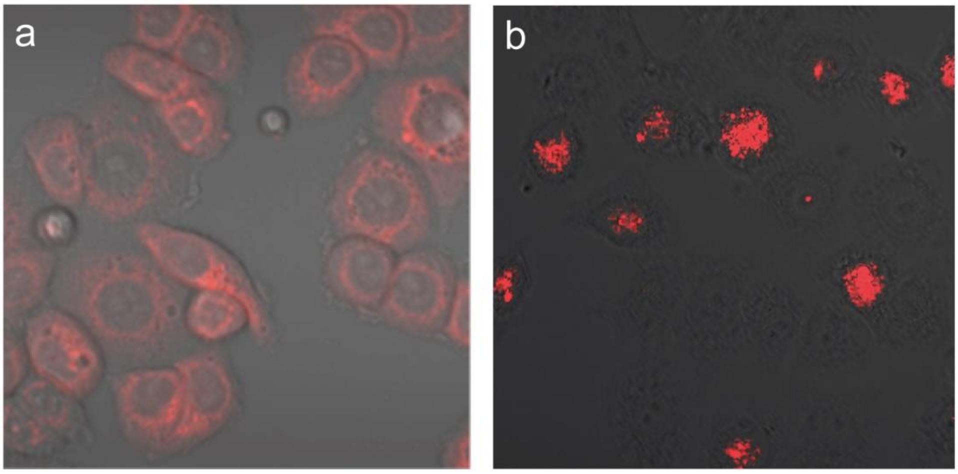

Figure S30. (a) Dark-field and **(b)** phase contrast microscope images merged with the luminescence of MDA-MB-468 cells after incubation with RhB-labeled MNPs.

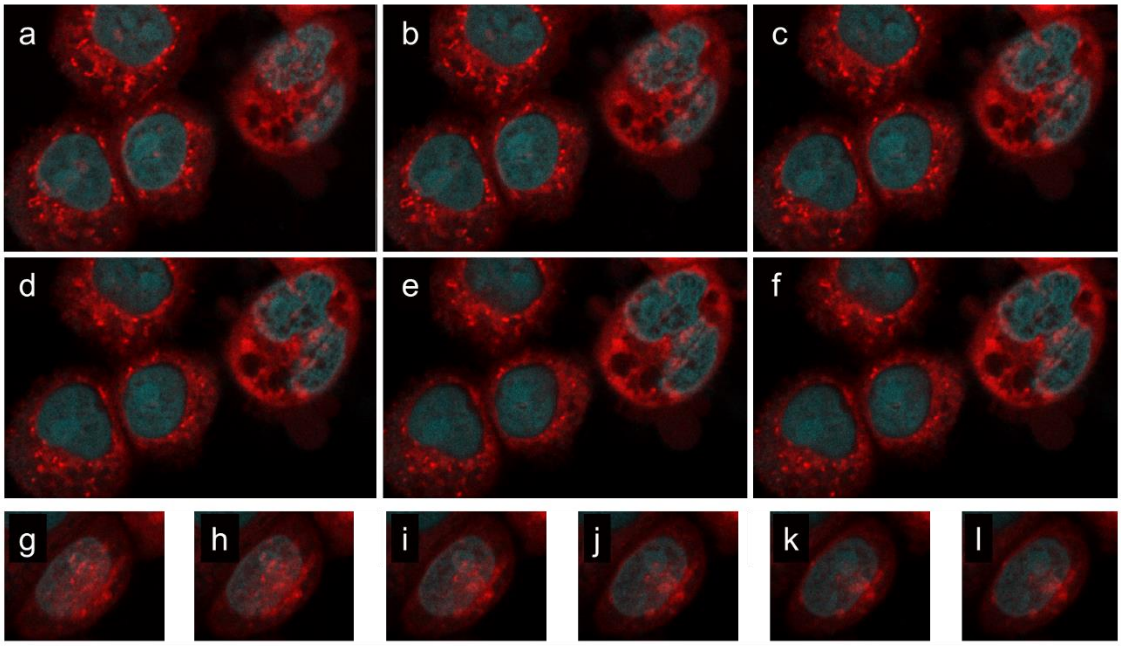

Figure S31. Merged microscope (Zeiss LSM 880) images of MDA-MB-468 cells after incubation with RhB-labeled MNPs and nuclei staining with DRAQ5^{TM} fluorescent probe. The (a-l) photographs were taken consecutively with a thickness of 0.9 μm and a depth increment of 0.432 μm showing the presence of NPs inside the nucleus.

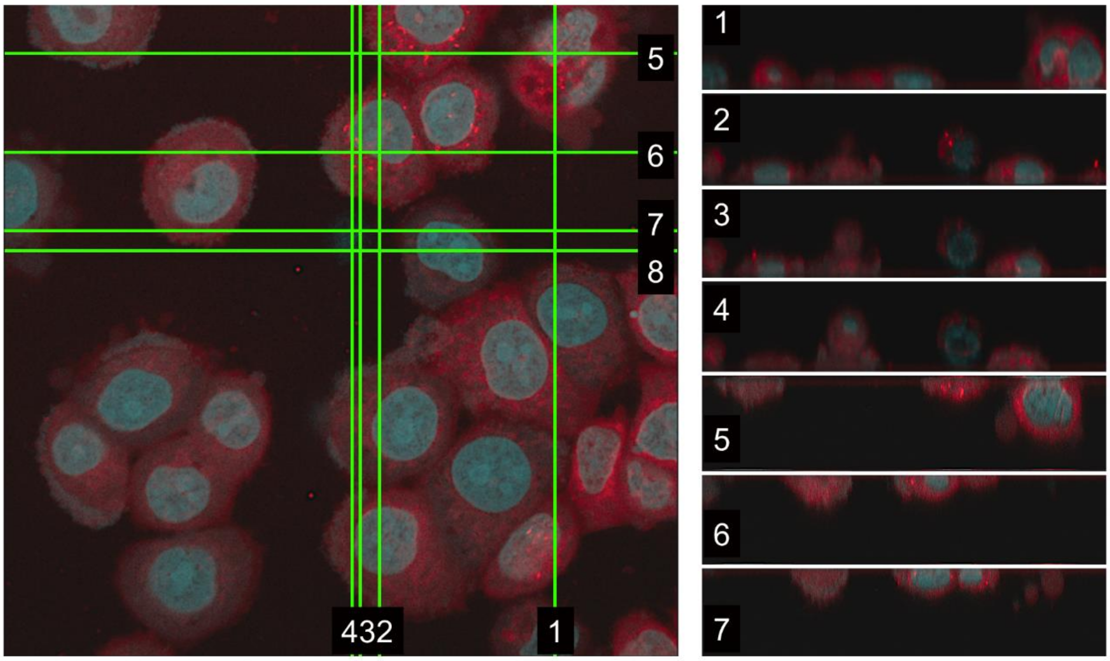

Figure S32. Merged DRAQ5™ and RhB images of XY, XZ, and YZ slices of MDA-MB-468 cells stained with DRAQ5™ after incubation with RhB-labeled MNPs.

Lysosomes and mitochondria colocalization experiments

Colocalization experiments of RhB-labeled MNPs in lysosomes and mitochondria were performed by confocal microscopy using the commercial fluorescent probes LysoTracker™ Green DND-26 and MitoTracker™ Green FM respectively. 50 000 cells were seeded in µ-Slide 8 Well plates (Ibidi) and grown in the presence of RhB-labeled MNPs for 24 h. Then, they were stained with either 150 nM LysoTracker or 200 nM MitoTracker for 30 min 37 °C. After staining, cells were washed three times with PBS and incubated in a culture medium for 30 min at 37°C to reduce unspecific staining. Then, cells were observed in a confocal microscope. The results are shown in Figure S33 and Figure S34. Superimposed images of red-labeled RhB MNPs and green-labeled lysosomes did not show any important color overlapping but separated red and green spots Figure S33d. Moreover, the size and distribution of lysosomes in cells were not changing after the internalization of the MNPs indicating that they are not engulfed by lysosomes.

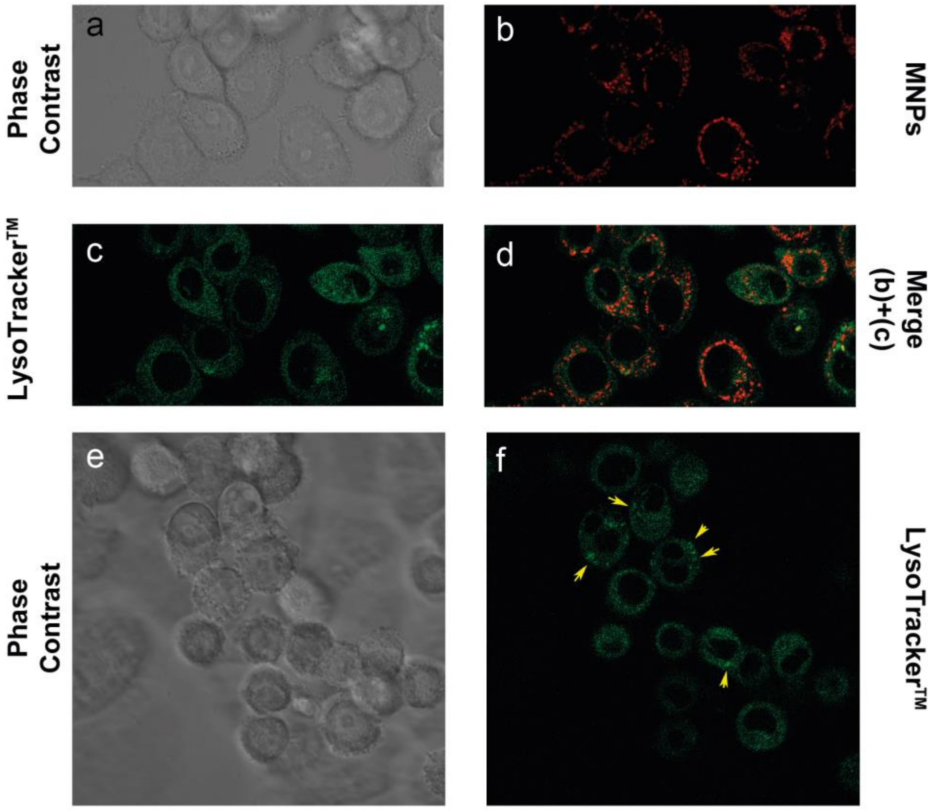

Figure S33. (a-d) Confocal colocalization experiments on RhB-labeled MNPs in lysosomes. **(e,f)** Phase-contrast and fluorescence images of lysosomes before MNPs internalization.

However, colocalization experiments on mitochondria showed a strong overlapping between the green and red spots of - MitoTracker^{TM} and red-labeled RhB MNPs, respectively, Table S6. Moreover, the internalization of NPs in the nucleus was also evident in these experiments (Figure S31 g-l). It can be inferred that the NPs can move freely in the cell interior and efficiently heat their local environment.

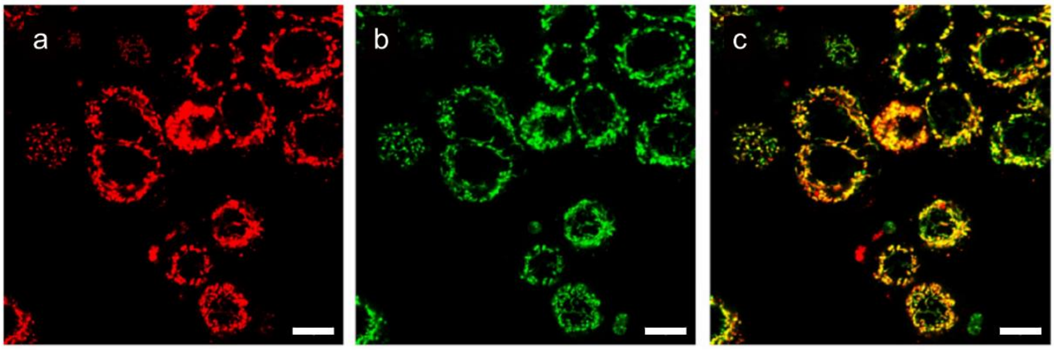

Figure S34. Confocal colocalization experiments on RhB-labeled MNPs in mitochondria. Fluorescence images of cells after internalization of the MNPs. The scale bars correspond to 100 μm.

Table S6. Statistical analysis of colocalization of MNPs with mitochondria

**Coefficient type**	**Original**
Pearson	$r = 0.652$
Overlap	$r = 0.704$ ($r = \sqrt{k_1 k_2}$, $k_1 = 0.659$ $k_2 = 0.752$)
Manders*	$M_1 = 0.933$<br>$M_1 = 0.933$
Costes*	Pearson's coefficient $r = 0.185$ (0.0 below thresholds)<br>$M_1 = 0.984$<br>$M_1 = 0.952$

*M_1 fraction of A overlapping B and M_2 fraction of B overlapping A

Colocalization of magnetic heaters and thermometric nanomicelles

To estimate the relative location of magnetic heaters and thermometric micelles, MDA-MB-468 cells were incubated with: i) heater MNPs labeled with RhB; ii) Sm^{3+}/Eu^{3+}-bearing thermometric nanomicelles labeled with Dy647; and iii) both of them. After washing, treatated cells were observed by confocal microscopy in comparison with non-treated cells. Fluorescence images of cells containing MNPs and thermometric micelles are shown in Figure S35. The colocalization of thermometric micelles and MNPs is evident in Figure S35a-c. Moreover, fluorescence line scans (Figure S35d-h) showed also thermometric micelles at a close distance of MNP heaters. In order to estimate the number of thermometric micelles in close contact with MNP heaters, a calculation of weighted colocalization coefficients of thermometric micelles in 54 confocal plane images was performed showing an average colocalization coefficient of 0.49.

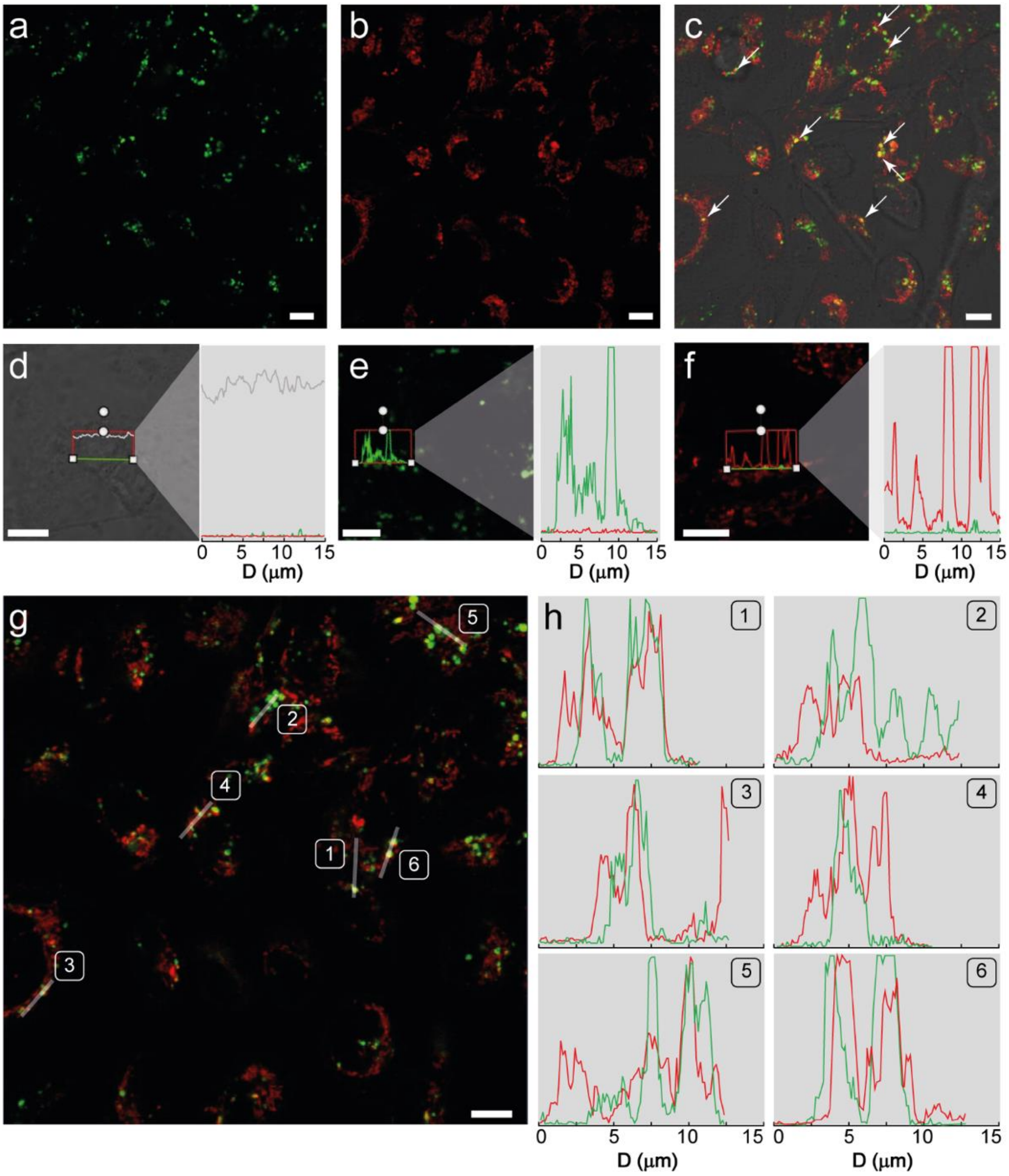

Figure S35. Confocal single-plane images of MDA-MB-468 cells incubated with heater MNPs labeled with RhB and Sm^{3+}/Eu^{3+}-bearing thermometric nanomicelles labeled with Dy647 observed in **(a)** Dy647 channel, **(b)** RhB channel and **(c)** merged channels. The arrows indicate sites containing MNPs labeled with RhB and Sm^{3+}/Eu^{3+}-bearing thermometric nanomicelles labeled with Dy647. Fluorescence line scans of MDA-MB-468 cells **(d)** without NPs and incubated with **(e)** Dy647 labeled Sm^{3+}/Eu^{3+}-bearing thermometric nanomicelles or **(f)** RhB labeled MNPs. The gray line in **(d)** corresponds to the background intensity while the red and green lines are the RhB and Dy647 intensities, respectively. **(g)** Illustrative regions of the image presented in **(c)** in which the fluorescence line scans displayed in **(h)** were performed. The scale bars are 10 μm.

The relative location of thermometric micelles (loaded with Gd^{3+} instead of Eu^{3+} and Sm^{3+} for better identification) with respect to MNP heaters was also examined by STEM of cell ultramicrotomes with a Titan (FEI) electron microscope. However, a large absorption of Mn and U contrast agents on the surface of the NPs prevented a rapid identification of both MNPs and nanomicelles, and therefore the localization of a representative number of NPs in the cells was not possible. Moreover, the signal of Fe from the MNPs could be clearly detected by EDS, but that of Gd was too weak.

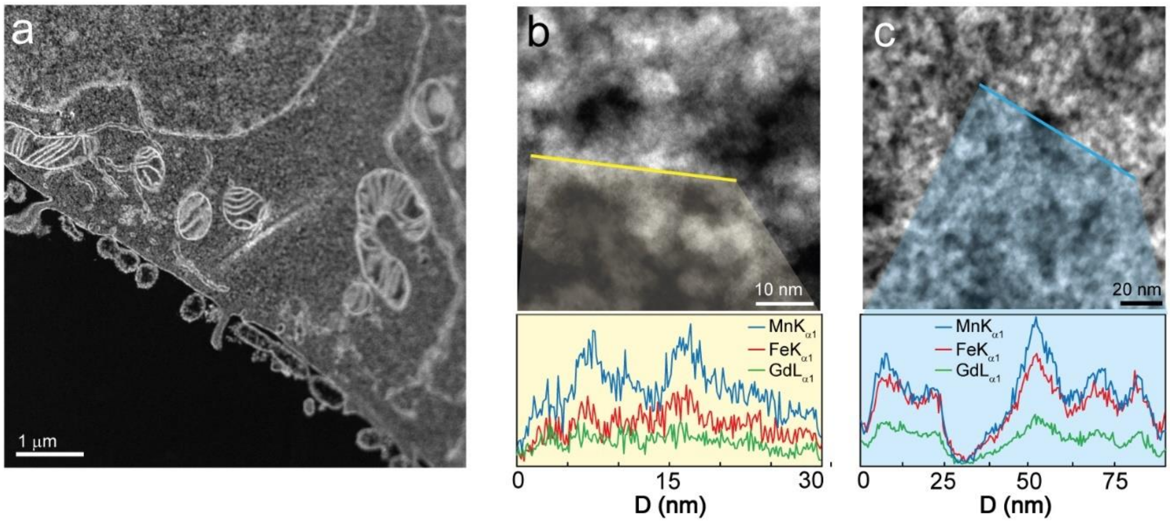

Figure S36. STEM observations of ultramicrotomes of MDA-MB-468 cells after incubation with MNPs and Gd^{3+}-doped nanomicelles. **(a)** Partial image of nucleus and cytoplasm at low magnification. **(b)**, **(c)** EDS concentration profiles of Mn, Fe, and Gd in different regions of the cells.

Cell death in local intracellular hyperthermia

Cell death experiments of intracellular hyperthermia were performed in an in-house developed AMF applicator at temperature-controlled conditions (T=37 ^{o}C). The magnet has a ferrite nucleus with a 2.5×2.5 cm^2 gap in which field intensity was uniform that allowed a simultaneous AMF application to 4 cell culture wells, one of them containing MDA-MB-468 cells with MNPs in the interior, and the rest were used as controls. The cell death ratio in the different samples was analyzed in a cytometer by the annexin assay.

VIII. Intracellular temperature evolution over time during exposure to an AMF

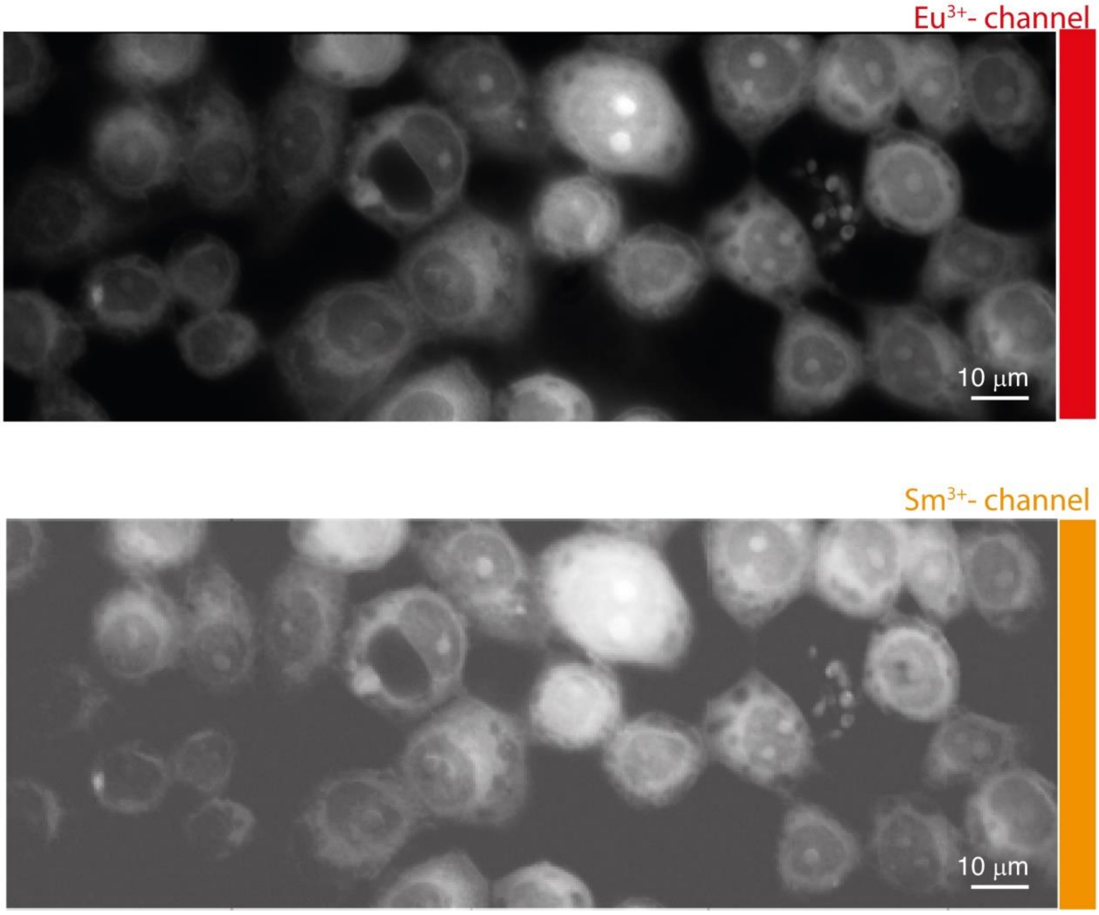

Figure S37. Illustrative intensity image of MDA-MB-468 cells internalized with the dual heater-thermometer core@shell NPs recorded in the Eu^{3+}- and Sm^{3+}-channels after 3 min of turned on an external AMF (Hf=2.4x10^{9} A·m^{-1}·s^{-1}).

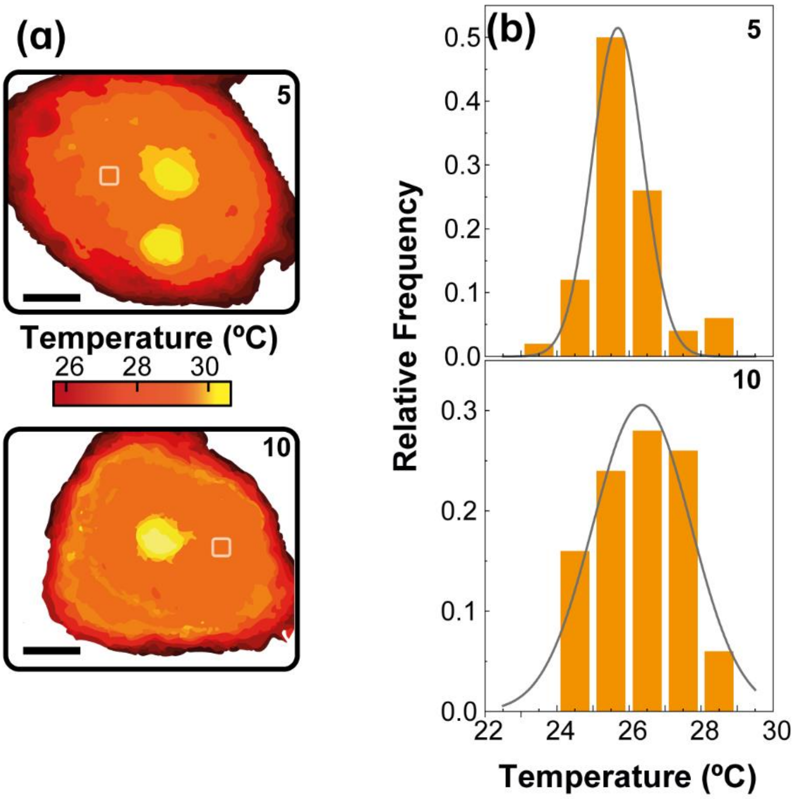

Figure S38. (a) Illustrative temperature maps of MDA-MB-468 cells internalized with the dual heater-thermometer core@shell NPs within the cytoplasm (orange region) after 3 min of exposure to an external AMF (Hf=2.4x10^{9} A·m^{-1}·s^{-1}). The scale bars correspond to 10μm. **(b)** Corresponding temperature histograms using 75 points, in the cytoplasm. The solid lines represent the Gaussian functions used to fit the experimental data (r^2>0.94). In each ROI, the distribution of the temperatures is well described by a Gaussian profile meaning that all the temperature readouts follow normal distributions.

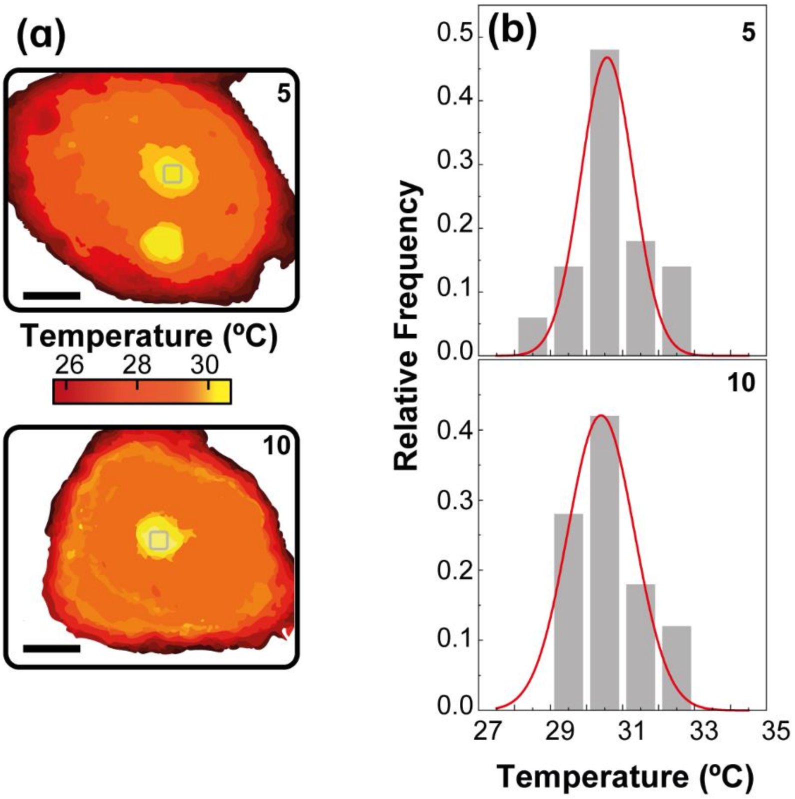

Figure S39. (a) Illustrative temperature maps of MDA-MB-468 cells internalized with the dual heater-thermometer core@shell NPs within the nucleolus (yellow region) after 3 min of exposure to an external AMF (Hf=2.4x10^{9} A·m^{-1}·s^{-1}). The scale bars correspond to 10μm. **(b)** Corresponding temperature histograms using 50 points in the nucleolus. The solid lines are the best fits to the data using Gaussian functions (r^2>0.90). In each ROI, the distribution of the temperatures is well described by a Gaussian profile meaning that all the temperature readouts follow normal distributions.

Table S7. Statistical analysis of the temperature histograms shown in Figure S38 and Figure S39.

**Cell**	**5**		**10**	
**Region**	*Cytoplasm*	*Nucleolus*	*Cytoplasm*	*Nucleolus*
**Mean Temperature**	25.7	30.4	25.3	30.6
**Standard Deviation**	0.7	0.9	0.9	0.7
r^2	0.97	0.90	0.94	0.93

The calculated value of the temperature in the nucleolus is, on average, about 5 ºC higher than the values measured in the cytoplasm, in agreement with previous reported results.[29]

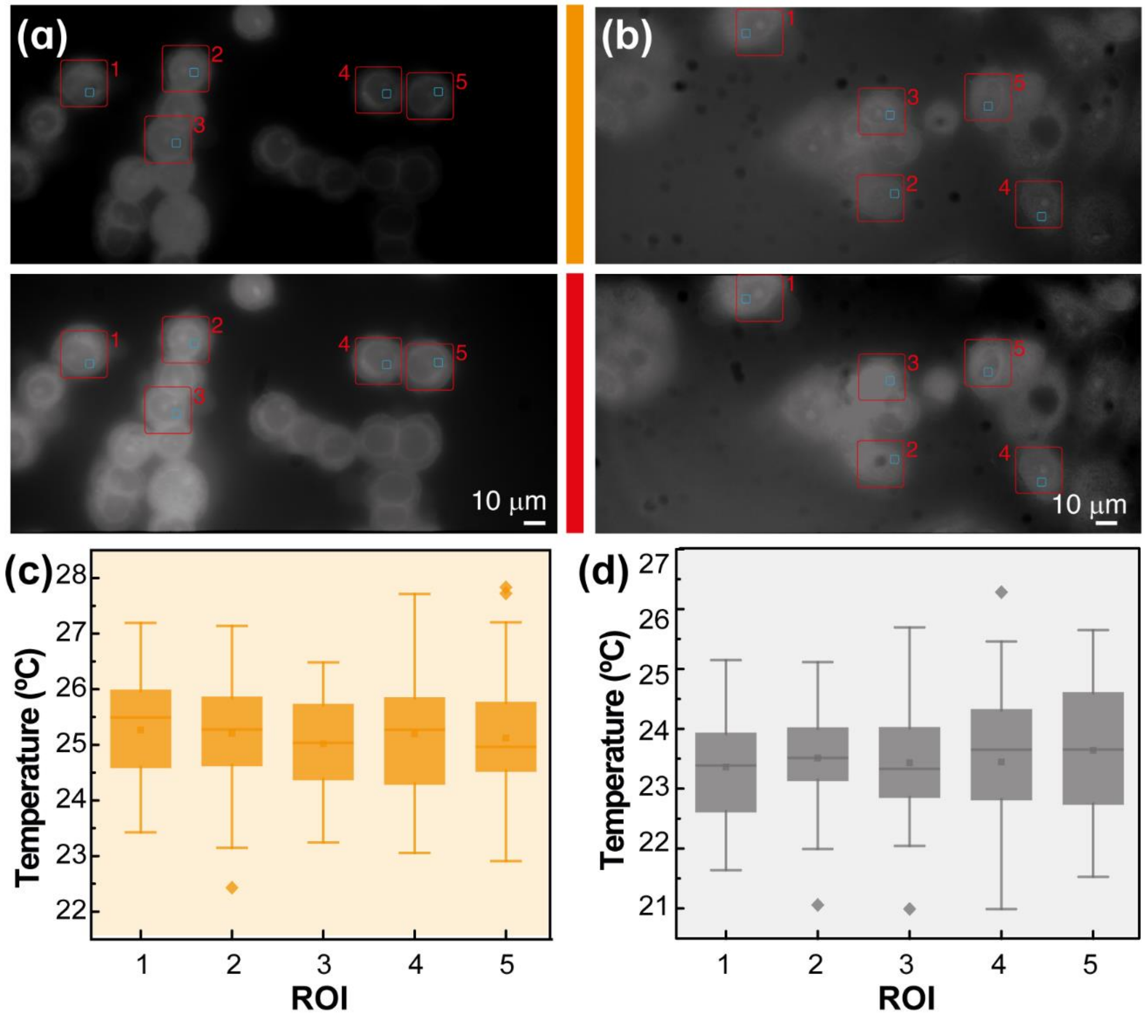

Figure S40. Illustrative intensity images of MDA-MB-468 cells internalized with the thermometric probes recorded in the Sm^{3+} (orange bar) and Eu^{3+} (red bar) channels for **(a)** experiment II and **(b)** experiment III, after 3 min of turned on an external AMF (Hf=2.4x10^9 A·m^{-1}·s^{-1}). The big squares identify the 5 analyzed cells and the small ones delimit the chosen ROI in each cell. The corresponding boxplots for n=75 points in the small squares are presented in **(c)** and **(d)**, respectively.

References

1. Rabin, Y. Is Intracellular Hyperthermia Superior to Extracellular Hyperthermia in the Thermal Sense? *Int. J. Hyperther.* **2002,** *18* (3), 194-202.
2. Keblinski, P.; Cahill, D. G.; Bodapati, A.; Sullivan, C. R.; Taton, T. A. Limits of Localized Heating by Electromagnetically Excited Nanoparticles. *J. Appl. Phys.* **2006,** *100* (5), 054305.
3. Ziabari, A.; Torres, P.; Vermeersch, B.; Xuan, Y.; Cartoixa, X.; Torello, A.; Bahk, J. H.; Koh, Y. R.; Parsa, M.; Ye, P. D.; Alvarez, F. X.; Shakouri, A. Full-Field Thermal Imaging of Quasiballistic Crosstalk Reduction in Nanoscale Devices. *Nat. Commun.* **2018,** *9*, 255.
4. Kozissnik, B.; Bohorquez, A. C.; Dobson, J.; Rinaldi, C. Magnetic Fluid Hyperthermia: Advances, Challenges, and Opportunity. *Int. J. Hyperther.* **2013,** *29* (8), 706-714.
5. Chiu-Lam, A.; Rinaldi, C. Nanoscale Thermal Phenomena in the Vicinity of Magnetic Nanoparticles in Alternating Magnetic Fields. *Adv. Funct. Mater.* **2016,** *26* (22), 3933-3941.
6. Gupta, A.; Kane, R. S.; Borca-Tasciuc, D.-A. Local Temperature Measurement in the Vicinity of Electromagnetically Heated Magnetite and Gold Nanoparticles. *J. Appl. Phys.* **2010,** *108* (6), 064901.
7. Faure, S.; Mille, N.; Kale, S. S.; Asensio, J. M.; Marbaix, J.; Farger, P.; Stoian, D.; van Beek, W.; Fazzini, P. F.; Soulantica, K.; Chaudret, B.; Carrey, J. Internal Temperature Measurements by X-Ray Diffraction on Magnetic Nanoparticles Heated by a High-Frequency Magnetic Field. *J. Phys. Chem. C* **2020,** *124* (40), 22259-22265.
8. Diaz-Puerto, Z. J.; Raya-Baron, A.; van Leeuwen, P. W. N. M.; Asensio, J. M.; Chaudret, B. Determination of the Surface Temperature of Magnetically Heated Nanoparticles Using a Catalytic Approach. *Nanoscale* **2021,** *13* (29), 12438-12442.
9. Davis, H. C.; Kang, S.; Lee, J. H.; Shin, T. H.; Putterman, H.; Cheon, J.; Shapiro, M. G. Nanoscale Heat Transfer from Magnetic Nanoparticles and Ferritin in an Alternating Magnetic Field. *Biophys. J.* **2020,** *118* (6), 1502-1510.
10. Dhar, A.; Kundu, A.; Kundu, A. Anomalous Heat Transport in One Dimensional Systems: A Description Using Non-Local Fractional-Type Diffusion Equation. *Front. Phys.* **2019,** *7*.
11. Cahill, D. G.; Braun, P. V.; Chen, G.; Clarke, D. R.; Fan, S. H.; Goodson, K. E.; Keblinski, P.; King, W. P.; Mahan, G. D.; Majumdar, A.; Maris, H. J.; Phillpot, S. R.; Pop, E.; Shi, L. Nanoscale Thermal Transport. Ii. 2003-2012. *Appl. Phys. Rev.* **2014,** *1* (1), 011305.
12. Polo-Corrales, L.; Rinaldi, C. Monitoring Iron Oxide Nanoparticle Surface Temperature in an Alternating Magnetic Field Using Thermoresponsive Fluorescent Polymers. *J. Appl. Phys.* **2012,** *111* (7), 07B334.
13. Riedinger, A.; Guardia, P.; Curcio, A.; Garcia, M. A.; Cingolani, R.; Manna, L.; Pellegrino, T. Subnanometer Local Temperature Probing and Remotely Controlled Drug Release Based on Azo-Functionalized Iron Oxide Nanoparticles. *Nano Lett.* **2013,** *13* (6), 2399-2406.
14. Dias, J. T.; Moros, M.; del Pino, P.; Rivera, S.; Grazu, V.; de la Fuente, J. M. DNA as a Molecular Local Thermal Probe for the Analysis of Magnetic Hyperthermia. *Angew. Chem. Int. Edit.* **2013,** *52* (44), 11526-11529.
15. Shah, R. R.; Dombrowsky, A. R.; Paulson, A. L.; Johnson, M. P.; Nikles, D. E.; Brazel, C. S. Determining Iron Oxide Nanoparticle Heating Efficiency and Elucidating Local Nanoparticle Temperature for Application in Agarose Gel-Based Tumor Model. *Mater. Sci. Eng. C Mater. Biol. Appl.* **2016,** *68*, 18-29.
16. Brites, C. D. S.; Balabhadra, S.; Carlos, L. D. Lanthanide-Based Thermometers: At the Cutting-Edge of Luminescence Thermometry. *Adv. Opt. Mater.* **2019,** *7* (5), 1801239.
17. Freddi, S.; Sironi, L.; D'Antuono, R.; Morone, D.; Dona, A.; Cabrini, E.; D'Alfonso, L.; Collini, M.; Pallavicini, P.; Baldi, G.; Maggioni, D.; Chirico, G. A Molecular Thermometer for Nanoparticles for Optical Hyperthermia. *Nano Lett.* **2013,** *13* (5), 2004-2010.

18. Silva, P. L.; Savchuk, O. A.; Gallo, J.; Garcia-Hevia, L.; Banobre-Lopez, M.; Nieder, J. B. Mapping Intracellular Thermal Response of Cancer Cells to Magnetic Hyperthermia Treatment. *Nanoscale* **2020,** *12* (42), 21647-21656.
19. Dong, J.; Zink, J. I. Taking the Temperature of the Interiors of Magnetically Heated Nanoparticles. *Acs Nano* **2014,** *8* (5), 5199-5207.
20. Huang, H.; Delikanli, S.; Zeng, H.; Ferkey, D. M.; Pralle, A. Remote Control of Ion Channels and Neurons through Magnetic-Field Heating of Nanoparticles. *Nat. Nanotechnol.* **2010,** *5* (8), 602-606.
21. Piñol, R.; Brites, C. D. S.; Bustamante, R.; Martínez, A.; Silva, N. J. O.; Murillo, J. L.; Cases, R.; Carrey, J.; Estepa, C.; Sosa, C.; Palacio, F.; Carlos, L. D.; Millán, A. Joining Time-Resolved Thermometry and Magnetic-Induced Heating in a Single Nanoparticle Unveils Intriguing Thermal Properties. *Acs Nano* **2015,** *9* (3), 3134-3142.
22. Clerc, P.; Jeanjean, P.; Hallali, N.; Gougeon, M.; Pipy, B.; Carrey, J.; Fourmy, D.; Gigoux, V. Targeted Magnetic Intra-Lysosomal Hyperthermia Produces Lysosomal Reactive Oxygen Species and Causes Caspase-1 Dependent Cell Death. *J. Control. Release* **2018,** *270*, 120-134.
23. Tong, S.; Quinto, C. A.; Zhang, L. L.; Mohindra, P.; Bao, G. Size-Dependent Heating of Magnetic Iron Oxide Nanoparticles. *Acs Nano* **2017,** *11* (7), 6808-6816.
24. Hara, K.; Sugihara, H.; Singh, L. P.; Islam, A.; Katoh, R.; Yanagida, M.; Sayama, K.; Murata, S.; Arakawa, H. New Ru(Ii) Phenanthroline Complex Photo Sensitizers Having Different Number of Carboxyl Groups for Dye-Sensitized Solar Cells. *J. Photoch. Photobio. A* **2001,** *145* (1-2), 117-122.
25. Bossmann, S. H.; Ghatlia, N. D.; Ottaviani, M. F.; Turro, C.; Durr, H.; Turro, N. J. Synthesis and Characterization of Nitroxide-Linked Ruthenium Complexes as Molecular Probes for Microheterogeneous Environments. *Synthesis* **1996,** *1996* (11), 1313-1319.
26. Agudelo, B. C.; Ochoa-Puentes, C.; Rodriguez-Córdoba, W.; Reiber, A.; Sierra, C. A. Synthesis, Characterization, X-Ray Crystal Structure and Dft Calculations of 4-([2, 2’: 6’, 2’’-Terpyridin]-4’-Yl) Phenol. *Rev. Colomb. de Quimica* **2018,** *47* (1), 77-85.
27. Hwang, J.-Y.; Seo, D.-S. Electro-Optical Characteristics for the Photoaligned Vertical Alignment Cell Using Photopolymer Surfaces Containing Chalconyl and Cholesterly Groups. *Liq. Cryst.* **2001,** *28* (7), 1065-1069.
28. Bonvin, D.; Hofmann, H.; Ebersold, M. M. Optimisation of Aqueous Synthesis of Iron Oxide Nanoparticles for Biomedical Applications. *J. Nanopart. Res.* **2016,** *18* (12), 1-16.
29. Piñol, R.; Zeler, J.; Brites, C. D. S.; Gu, Y. Y.; Tellez, P.; Neto, A. N. C.; da Silva, T. E.; Moreno-Loshuertos, R.; Fernandez-Silva, P.; Gallego, A. I.; Martinez-Lostao, L.; Martinez, A.; Carlos, L. D.; Millán, A. Real-Time Intracellular Temperature Imaging Using Lanthanide Bearing Polymeric Micelles. *Nano Lett.* **2020,** *20* (9), 6466-6472.
30. Connord, V.; Clerc, P.; Hallali, N.; El Hajj Diab, D.; Fourmy, D.; Gigoux, V.; Carrey, J. Real-Time Analysis of Magnetic Hyperthermia Experiments on Living Cells under a Confocal Microscope. *Small* **2015,** *11* (20), 2437-45.
31. Brites, C. D. S.; Millán, A.; Carlos, L. D. Lanthanides in Luminescent Thermometry. In *Handbook on the Physics and Chemistry of Rare Earths*, Bünzli, J.-C. G.; Pecharsky, V. K., Eds. Elsevier Science, B. V.: Amsterdam, 2016; Vol. 49, pp 339-427.
32. Gu, Y.; Yoshikiyo, M.; Namai, A.; Bonvin, D.; Martinez, A.; Pinol, R.; Téllez, P.; Silva, N. J.; Ahrentorp, F.; Johansson, C. Magnetic Hyperthermia with ε-Fe_2O_3 Nanoparticles. *RSC Adv.* **2020,** *10* (48), 28786-28797.
